# Effects of herbal medicines and their phytoconstituents on vagus nerve stimulation: a systematic review of *in vivo* studies

**DOI:** 10.3389/fphar.2026.1866222

**Published:** 2026-08-12

**Authors:** Lin Ang, Boram Lee, Yixian Quah, Eunhye Song, Chan-Young Kwon

**Affiliations:** 1 Department of Preventive Medicine, College of Korean Medicine, Gachon University, Seongnam, Republic of Korea; 2 KM Science Research Division, Korea Institute of Oriental Medicine, Daejeon, Republic of Korea; 3 Developmental and Reproductive Toxicology Research Group, Korea Institute of Toxicology, Daejeon, Republic of Korea; 4 Global Cooperation Center, Korea Institute of Oriental Medicine, Daejeon, Republic of Korea; 5 Department of Oriental Neuropsychiatry, College of Korean Medicine, Dong-eui University, Busan, Republic of Korea

**Keywords:** cholinergic anti-inflammatory pathway, herbal medicine, *in vivo*, phytoconstituents, vagal outcomes

## Abstract

**Background:**

The vagus nerve regulates autonomic, inflammatory, metabolic, and neurobehavioral functions. Although invasive vagus nerve stimulation (VNS) is gaining clinical relevance, herbal medicines may represent accessible, non-invasive modulators of vagal activity. However, the extent and nature of *in vivo* evidence supporting these effects remain unclear. This systematic review evaluates herbal medicines and phytoconstituents that modulate vagus nerve-related outcomes in animal models.

**Methods:**

A systematic search was conducted in PubMed, Embase, Cochrane Library, ScienceDirect, China National Knowledge Infrastructure (CNKI), Wanfang, Citation Information by National Institute of Informatics (CiNii), Ichushi Web, Research Information Service System (RISS), and Oriental Medicine Advanced Searching Integrated System (OASIS) databases for *in vivo* studies evaluating the impact of herbal medicines, extracts or phytoconstituents on vagus nerve activity, vagal tone, or vagus-mediated physiological functions. Inclusion criteria included studies using intact animal models with direct assessments of vagally mediated outcomes. Risk of bias was evaluated using the Systematic Review Centre for Laboratory Animal Experimentation (SYRCLE) tool.

**Results:**

Out of 10,342 studies screened, 75 studies met inclusion criteria. Interventions included multi-herb prescriptions (n = 22), single-herb extracts (n = 15), phytoconstituents (n = 29), phytoconstituent derivatives or formulations (n = 6), and essential oils (n = 3). Across cardiovascular, neurological, inflammatory, metabolic, and gastrointestinal models, interventions frequently increased acetylcholine (ACh) levels, choline acetyltransferase (ChAT) activity, α7 nicotinic acetylcholine receptor (α7 nAChR) expression, and heart rate variability (HRV) indices, while decreasing acetylcholinesterase (AChE) activity and pro-inflammatory cytokines (p < 0.05–0.001). Proposed mechanisms predominantly involved enhancement of cholinergic neurotransmission, suggesting the involvement of the cholinergic anti-inflammatory pathway.

**Conclusion:**

Evidence from preclinical studies indicates that certain herbal medicines and phytoconstituents can modulate vagus nerve activity, primarily through anti-inflammatory and autonomic regulation pathways. Rigorous mechanistic studies and translational research are needed to determine the clinical relevance of herbal-induced vagal modulation, particularly given that many preclinical studies employed parenteral administration routes that may not reflect the bioavailability and first-pass metabolism associated with oral herbal use in humans.

## 1 Introduction

The vagus nerve is a central component of the parasympathetic branch of the autonomic nervous system and plays a crucial role in maintaining physiological homeostasis (Prescott and Liberles, 2022). It innervates multiple organs, including the heart, lungs, gastrointestinal tract, and immune-related tissues, and governs essential functions such as heart rate regulation, digestion, inflammatory control, and bidirectional communication between the gut and brain (Yuan and Silberstein, 2016). Through its role in the cholinergic anti-inflammatory pathway, the vagus nerve is also critically involved in the modulation of systemic immune responses and neuroinflammation (Pavlov and Tracey, 2012). Consequently, vagal dysfunction has been implicated in a wide range of pathological conditions, including inflammatory bowel disease, neurodegenerative disorders, and metabolic diseases (Liu et al., 2025).

In recent decades, vagus nerve stimulation (VNS) has emerged as a promising therapeutic strategy for modulating autonomic and inflammatory pathways (Chan and Mani, 2025). Implantable VNS devices have demonstrated clinical efficacy in conditions such as epilepsy, depression, and inflammatory disorders (Courties et al., 2021; Fang et al., 2023; Shao et al., 2023). However, the invasive nature of these devices, along with their cost, limited accessibility, and adverse effects, has motivated the exploration of alternative, non-invasive approaches to vagal modulation (Clancy et al., 2014; Liu et al., 2024).

Among these alternatives, herbal medicines and their bioactive constituents have attracted increasing attention for their potential to influence vagal tone and vagus-mediated physiological processes (Zou et al., 2017; Qi et al., 2019; Bai et al., 2025). Traditional medical systems, including Ayurveda, traditional Chinese medicine, traditional Korean medicine, and Western herbalism, have long employed herbal interventions to regulate autonomic balance, alleviate inflammation, modulate the hypothalamic–pituitary–adrenal axis, and support gastrointestinal function (Manabe et al., 2010; Wang X. H. et al., 2022; Challal et al., 2023). These physiological effects intersect closely with vagus nerve activity, suggesting a plausible mechanistic basis for herbal-induced vagal modulation.

Despite growing interest in this area, a comprehensive synthesis of *in vivo* evidence examining the effects of herbal medicines and phytoconstituents on vagus nerve function remains lacking. Animal models provide a valuable platform for investigating both direct neural effects and indirect markers of vagal activity, including electrophysiological measures, autonomic indices, neurochemical signaling, and immune modulation (Mukherjee et al., 2022; Chang and Grieder, 2024). Systematic evaluation of this preclinical literature is essential for identifying promising herbal candidates, clarifying mechanisms of action, and informing translational research. Therefore, this systematic review aimed to critically evaluate *in vivo* studies investigating the effects of herbal medicines and their phytoconstituents on vagus nerve stimulation and vagus-mediated physiological outcomes.

## 2 Methods

This systematic review was reported in accordance with the Preferred Reporting Items for Systematic Reviews and Meta-Analyses (PRISMA) guidelines (Page et al., 2021). The review protocol was prospectively registered with the International Prospective Register of Systematic Reviews (PROSPERO) under the registration number CRD420251060757.

### 2.1 Eligibility criteria

#### 2.1.1 Population

Healthy animals or animals subjected to disease models (e.g., rats, mice, rabbits) were eligible for inclusion. No restrictions were placed on sex, strain, or age.

#### 2.1.2 Intervention

Eligible interventions included herbal medicines (multi-herb formulas, crude extracts, and single-herb preparations) and phytoconstituents (purified bioactive compounds or their formulations/derivatives) derived from medicinal plants and administered via any route (oral, topical, or injection). Synthetic compounds not originating from plant sources were excluded.

#### 2.1.3 Comparator

Comparator groups included vehicle controls, sham-treated animals, positive controls, or other non-herbal comparators (conventional pharmacological agents or baseline conditions), depending on study design.

#### 2.1.4 Outcomes

Studies were required to report at least one outcome reflecting vagus nerve stimulation or vagus-mediated physiological function. Eligible outcomes included electrophysiological recordings of vagal activity; heart rate variability indices indicative of vagal tone; neurochemical markers of cholinergic signaling (e.g., acetylcholine, choline acetyltransferase); and inflammatory mediators associated with the cholinergic anti-inflammatory pathway (e.g., TNF-α, IL-1β, IL-6). Studies that did not report any relevant vagal or vagus-mediated outcome were excluded.

#### 2.1.5 Study design

Experimental *in vivo* animal studies evaluating herbal medicines or phytoconstituents and reporting vagal outcomes were included. Reviews, meta-analyses, clinical trials, *in vitro*, *ex vivo*, or *in silico* studies, conference abstracts, editorials, and case reports were excluded.

### 2.2 Information sources and search strategy

A comprehensive literature search was conducted across multiple databases from inception to August 2025. These databases included PubMed, Embase, Cochrane Library, ScienceDirect, Oriental Medicine Advanced Searching Integrated System (OASIS), Research Information Service System (RISS), China National Knowledge Infrastructure (CNKI), Wanfang Data, and CiNii (Citation Information by NII). To maximize inclusivity and minimize language bias, no language restrictions were applied. The search strategy was developed using combinations of keywords and controlled vocabulary. The terms used spanned three main thematic domains: animal models (e.g., “rat”, “mouse”, “murine”, “rabbit”), vagus/autonomic nervous system (e.g., “vagus nerve”, “parasympathetic”, “autonomic nervous system”), and herbal medicine (e.g., “herbal medicine”, “plant extract”, “phytotherapy”). In addition to electronic searches, reference lists of included studies and relevant reviews were manually screened to identify additional eligible studies. The full search strategy for each database was provided in Supplementary Table S1.

### 2.3 Study selection

All retrieved records were imported into EndNote 21 software, and duplicate entries were removed. Two reviewers (LA and BL) independently screened titles and abstracts according to predefined eligibility criteria. Full-text articles were subsequently assessed for inclusion. Disagreements were resolved through discussion, with arbitration by a third reviewer (CYK) when necessary.

### 2.4 Data extraction

Data extraction was performed independently by two reviewers (LA and BL) using a standardized, pilot-tested extraction form. Any discrepancies between reviewers were resolved through consensus or adjudication by a third reviewer (CYK). Extracted data included study design characteristics (randomization, blinding, control groups, and number of experimental groups), animal characteristics (species, strain, sex, age, weight, sample size, and disease model), intervention details (herbal agent or compound, dosage, route of administration, frequency, and treatment duration), and vagal-related outcomes (primary results and statistical significance). Bibliographic information (authors, year, journal, and country of origin) was also recorded. When data were missing or unclear, corresponding authors were contacted for clarification.

### 2.5 Risk of bias assessment

Risk of bias was independently assessed by two reviewers (LA and ES) using the Systematic Review Centre for Laboratory Animal Experimentation (SYRCLE) Risk of Bias tool, which is specifically designed for animal intervention studies (Hooijmans et al., 2014). The tool evaluates ten domains, including sequence generation, baseline characteristics, allocation concealment, random housing, blinding of caregivers, random outcome assessment, blinding of outcome assessors, incomplete outcome data, selective outcome reporting, and other potential sources of bias. Each domain was rated as having a “low,” “high,” or “unclear” risk of bias. Disagreements were resolved by consensus.

### 2.6 Data synthesis

Due to substantial heterogeneity in animal species, disease models, interventions, and outcome measures, quantitative meta-analysis was not considered appropriate, and findings were synthesized narratively. Findings were presented based on the type of herbal medicine or constituent, the species of animal used, and the specific outcome measures reported. Interventions were classified into four main categories: herbal medicines (including multi-herb prescriptions and single-herb extracts), phytoconstituents, phytoconstituent derivatives or formulations, and essential oils. This classification facilitated differentiation between effects attributable to whole-herb synergism and those driven by isolated bioactive compounds or optimized formulations.

## 3 Results

### 3.1 Study selection

**FIGURE 1:**
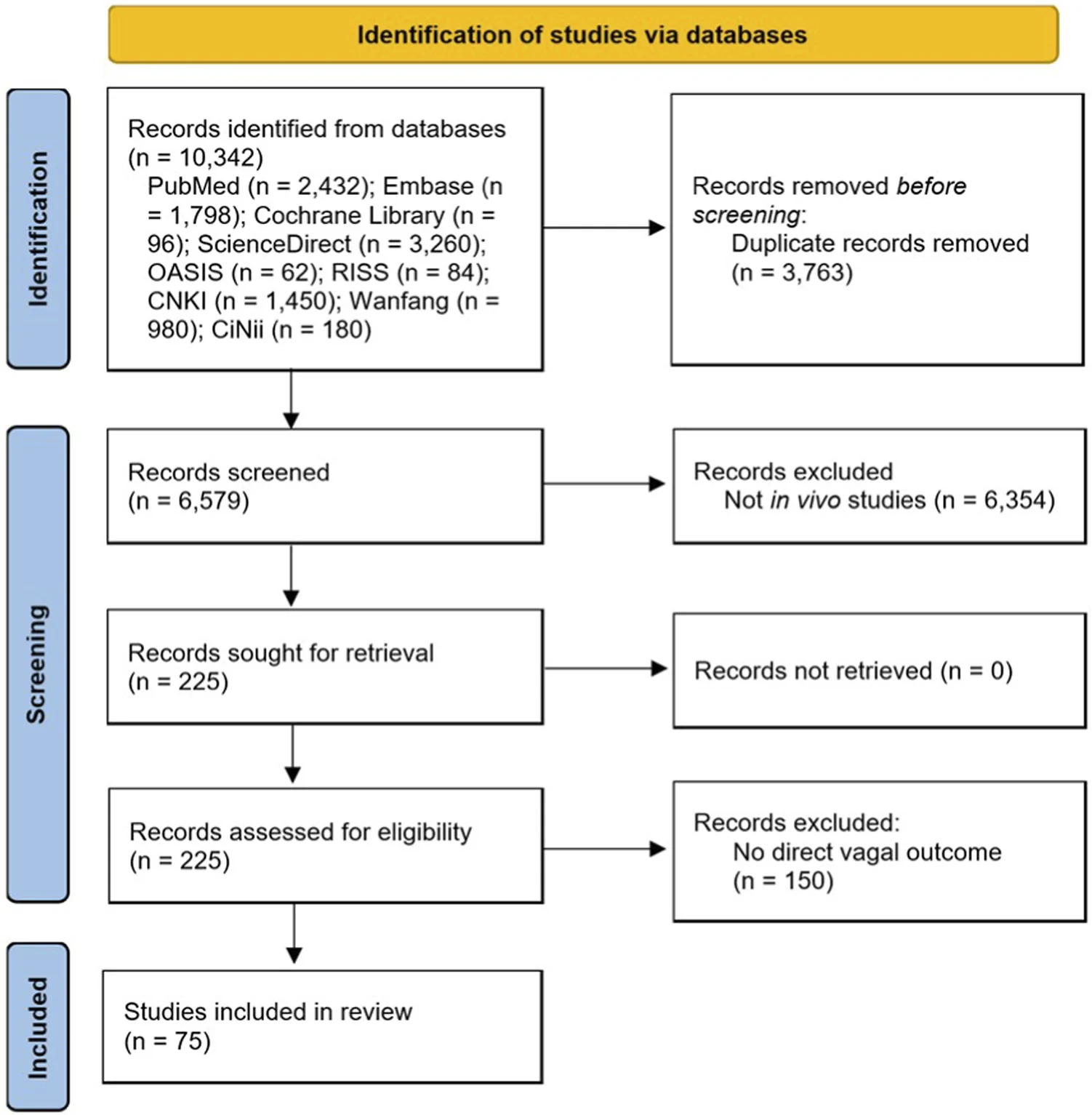
Study selection process.

The electronic database search and manual screening yielded a total of 10,342 records. After removal of duplicates and sequential screening of titles, abstracts, and full texts according to the predefined eligibility criteria, 75 *in vivo* studies met the inclusion criteria and were included in the review. The study selection process is summarized in the PRISMA flow diagram (Figure 1).

### 3.2 Study characteristics

All included studies were experimental *in vivo* animal studies evaluating the effects of herbal medicines, phytoconstituents, or their derivatives on markers of vagus nerve activity or vagus-mediated physiological outcomes. The studies were published between 1983 and 2025. The majority originated from China (50/75), with a smaller number conducted in Egypt, Japan, and Korea. Most studies were described as randomized animal experiments (69/75), while six were non-randomized. Commonly used animal models included Wistar rats (25/75) and Sprague–Dawley rats (21/75). A minority of studies employed cats, dogs, guinea pigs, rabbits, zebrafish, or hypertensive rat substrains.

Disease models represented a wide spectrum of physiological systems, with concentrations in neurodegenerative and neurological models (16/75), followed by cardiovascular or hypertensive models (11/75) and ischemic or cerebrovascular models (7/75). These were followed in frequency by metabolic or diabetic models, stress or autonomic dysregulation models, and sepsis or systemic inflammatory models, among others. In addition, seven studies investigated physiological (non-disease) conditions, focusing on vagal function without a pathological trigger. Out of the 75 included studies, most studies (60/75) reported total sample sizes explicitly, while the remaining 15 provided incomplete or unreported data. Most experiments used 30 to 60 animals in total, corresponding to 5 to 10 animals per group across 4 to 6 experimental groups.

Across studies, vagal modulation was evaluated using biochemical, electrophysiological, and functional measures. The most common outcomes were acetylcholine (ACh; 42/75), acetylcholinesterase (AChE/TChE; 26/75), and choline acetyltransferase (ChAT; 19/75), representing the primary markers of cholinergic activity. Gene or protein expression related to cholinergic signaling was reported in 21 studies, and α7 nicotinic acetylcholine receptor (α7 nAChR) expression in five studies. Heart rate variability (HRV) appeared in five studies, while cytokines associated with the cholinergic anti-inflammatory pathway (TNF-α, IL-6, IL-1β, NF-κB) were reported in five studies. A few investigations assessed reflex responses (4/75) or vagal discharge (3/75). Overall, most evidence derived from biochemical markers, with fewer studies employing direct physiological or reflex-based assessments of vagal activity.

Interventions were classified into four main categories: herbal medicines (37/75), phytoconstituents (29/75), phytoconstituent derivatives or formulations (6/75), and essential oils (3/75). Among herbal medicines, multi-herb traditional prescriptions were most common (22/75), including formulas such as Guizhi Decoction, Sini Decoction, and Liu Wei Dihuang Decoction. Single-herb extracts (15/75) involved extracts from Pinellia ternata, Scutellaria baicalensis, Drynaria fortunei, and Zingiber officinale. Within phytoconstituent studies, curcumin was the most frequently investigated compound, while others included Angelica sinensis polysaccharides, puerarin, menthol, and capsaicin. Phytoconstituent derivatives or formulations included nanocurcumin and curcumin nanoparticles while essential oils included geranium and lavender.

### 3.3 Risk of bias

**FIGURE 2:**
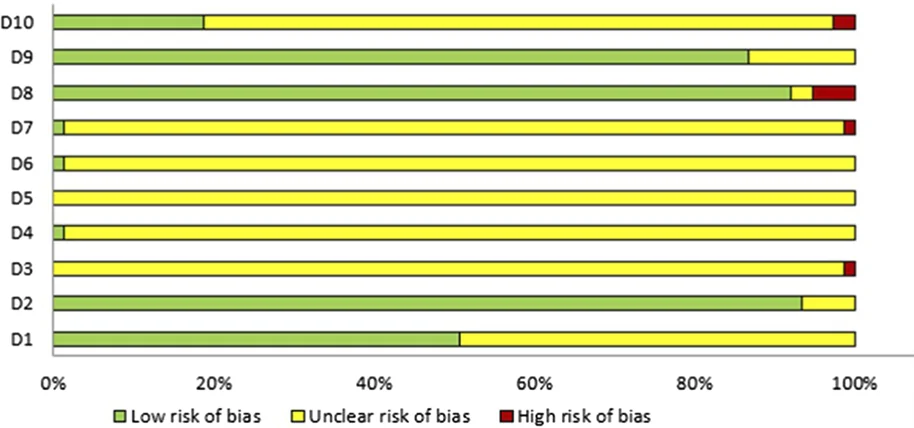
Risk of bias assessment of included studies using the SYRCLE tool.

Figure 2 provides the risk of bias assessment to each study in each of the SYRCLE items. Sequence generation was low risk in 38 studies (51%) and unclear in 37 (49%). Baseline comparability was well maintained, rated low in 68 (91%) studies, while allocation concealment was unclear in 74 (99%). Random housing was seldom described, with 73 (97%) unclear, and caregiver blinding was reported in fewer than five studies (7%). Outcome assessor blinding was low in six (8%) and unclear in 69 (92%). Attrition bias was low in 61 studies (81%), and selective outcome reporting was low in 64 (85%). Other potential biases, such as funding or design-related issues, were unclear in 70 (93%). Overall, baseline comparability, data completeness, and outcome reporting were generally robust, while allocation concealment and blinding were poorly documented. The high prevalence of “unclear” risk judgments, particularly in critical domains such as allocation concealment (99%) and random housing (97%), highlights substantial deficiencies in reporting quality. Because inadequate reporting precludes reliable assessment of study conduct, the possibility of underlying methodological limitations cannot be excluded. Detailed assessments for individual studies are provided in Supplementary Table S2.

### 3.4 Effects of herbal medicines and phytoconstituents on vagal modulation

A total of 75 *in vivo* studies examined the effects of herbal medicines, phytoconstituents, or their derivatives on vagus nerve–related outcomes across diverse disease models. Despite substantial heterogeneity in species, experimental design, intervention type, and outcome measures, the collective evidence demonstrated a consistent enhancement of vagal activity, cholinergic signaling, and vagus-mediated anti-inflammatory responses. A complete list of included interventions and their experimental characteristics is presented in Supplementary Tables S3–S7.

#### 3.4.1 Multi-herb prescriptions

Twenty-two preclinical studies (Zhang Y. P. et al., 2005; Zhang Z. et al., 2005; Du et al., 2006; Du et al., 2007; Wang et al., 2011; Zhang et al., 2011; Jiang et al., 2013; Li et al., 2014; Qi, 2014; Li et al., 2015; Lu et al., 2015; Wu et al., 2015; Xie et al., 2015; Jiang et al., 2016; Ma and Zhang, 2016; Tan et al., 2017; Bai et al., 2019; Wu et al., 2019; Sun et al., 2022; Gan et al., 2023; Nakyam et al., 2023; Sun et al., 2025) evaluated the effects of multi-herb prescriptions on vagal and cholinergic regulation across cardiovascular, neurological, gastrointestinal, respiratory, and metabolic disease models (Table 1). Most interventions significantly increased acetylcholine (ACh) levels or choline acetyltransferase (ChAT) activity and decreased acetylcholinesterase (AChE), tyrosine hydroxylase (TH), or norepinephrine (NE) (p < 0.05–0.001), indicating enhanced parasympathetic activity and attenuation of sympathetic overactivation. Mechanistic outcomes included improvements in autonomic balance, neuroimmune modulation, neurotrophic signaling, and gastrointestinal neural integrity. Several formulations directly enhanced vagal efferent discharge or baroreflex sensitivity, while others normalized hypothalamic–vagal excitability or reduced vagal-mediated hypersensitivity responses. Notably, this modulation was context-dependent, while most formulas enhanced vagal tone, certain prescriptions such as Banxia Xiexin Decoction protected the gastric mucosa by downregulating pathologically excessive vagal excitability via reduced ChAT expression. Similarly, Yunpi Prescription normalized central satiety feedback in anorexia models by reducing vagal–hypothalamic hyperexcitability. Furthermore, prescriptions like Guizhi Decoction demonstrated a rebalancing effect on the sympathetic–vagal tone by concurrently increasing ChAT and reducing TH expression in cardiac models. Detailed study characteristics are provided in Supplementary Table S3.

**TABLE 1:** Summary of studies on vagal modulation by multi-herb prescriptions.

Study (ref)	Disease model	Intervention (dose, period)	Main vagal outcomes	Summary of findings
Wang et al. (2011)	Spontaneously hypertensive rats	Banxia baizhu tianma decoction 4.32 g/kg/day for 18–32 weeks	1. ACh-dependent relaxation significantly increased (p < 0.01) 2. NO concentration significantly decreased (p < 0.01) 3. Total antioxidant capacity significantly increased (p < 0.01) 4. IL-1 and IL-6 expression significantly decreased (p < 0.05)	Improved ACh-mediated endothelial relaxation by reducing iNOS and IL-1 and enhancing antioxidant regulation
Lu et al. (2015)	NOD mouse Sjögren’s syndrome	Banxia qinlian decoction 60 g/kg/day for 12 weeks	1. IL-17 and IL-6 expression significantly decreased (p < 0.01) 2. M3R expression significantly decreased (p < 0.05) 3. AQP5 expression significantly decreased (p < 0.05)	Downregulated IL-17/IL-6–M3R axis, reducing cholinergic-mediated glandular inflammation
Zhang et al. (2005b)	Stress-induced gastric ulcer rats	Banxia xiexin decoction 3 mL twice daily for 3 days	1. ChAT expression in brain significantly increased in model rats (p < 0.01) 2. ChAT expression in brain and gastric tissue significantly decreased after treatment (p < 0.01)	Decreased excessive vagal excitability by downregulating ChAT expression, protecting gastric mucosa
Du et al. (2007)	Juvenile anorexia rats	Baobaole oral liquid once daily for 3 weeks	1. Lateral hypothalamic neuron excitatory response to vagus stimulation significantly normalized (p < 0.01) 2. Ventromedial nucleus inhibitory neuron response significantly restored (p < 0.01) 3. Glucose-sensitive neuron distribution significantly normalized (p < 0.01)	Restored vagal–hypothalamic excitability balance and appetite regulation
Xie et al. (2015)	Multiple organ dysfunction syndrome rats	Dachengqi decoction 2 mL/rat (1 g/mL) twice daily for 2 days	1. ACh-positive nerve fibers significantly increased (p < 0.01) 2. SP-positive nerve fibers significantly increased (p < 0.01) 3. Vasoactive intestinal peptide-positive fibers significantly increased (p < 0.01) 4. Nitric oxide synthase-positive fibers significantly increased (p < 0.01)	Protected cholinergic and inhibitory enteric neurons, restoring gastrointestinal neural integrity
Zhang et al. (2005a)	Low-protein diet anorexia rats	Er’bao granule 3.76 g/100 g/day for 3 weeks	1. Food intake significantly increased (p < 0.05) 2. Glucose-sensitive neuron proportion in LHA significantly increased (p < 0.05)	Restored glucose-sensitive hypothalamic vagal neurons to normalize ingestion behavior
Jiang et al. (2016)	6-OHDA cardiac denervation rats	Guizhi decoction 4–5.5 g/kg/day for 10 days	1. NE and TH levels significantly increased in model rats (p < 0.01) and significantly decreased after treatment (p < 0.01) 2. ChAT expression significantly increased (p < 0.01) 3. TH/ChAT ratio significantly decreased (p < 0.01)	Rebalanced sympathetic–vagal tone by increasing ChAT and reducing TH expression
Li et al. (2014)	Diabetic cardiac autonomic neuropathy rats	Guizhi decoction 4.0 g/kg/day for 4 weeks	1. HRV indices (SDNN, RMSSD, HF) significantly increased (p < 0.05) 2. TH expression significantly decreased (p < 0.05) 3. ChAT expression significantly increased (p < 0.05)	Enhanced vagal tone and reduced sympathetic overactivity, improving cardiac autonomic regulation
Li et al. (2015)	Diabetic cardiac autonomic neuropathy rats	Guizhi decoction 4.0–5.5 g/kg/day (1:1, 2:1, 1:2 ratios) for 4 weeks	1. TH and NGF expression significantly decreased (p < 0.05) 2. ChAT and CNTF expression significantly increased (p < 0.01)	The 1:1 guizhi–Shaoyao ratio most effectively restored cardiac autonomic balance by enhancing cholinergic markers
Sun et al. (2022)	OVA + cigarette smoke–induced asthma mice	Houpo-mahuang decoction 6–12 g/kg/day for 14 days	1. Airway hyperresponsiveness significantly decreased (p < 0.01) 2. TRPA1 expression significantly decreased (p < 0.01) 3. Zonula occludens-1 and occludin expression significantly increased (p < 0.01) 4. Claudin-2 expression significantly decreased (p < 0.01)	Reduced neurogenic airway hypersensitivity via TRPA1 inhibition and restoration of epithelial integrity
Sun et al. (2025)	Intracerebral hemorrhage rats	Liangxue tongyu prescription 10–20 g/kg/day for 3 days	1. Vagus nerve discharge frequency significantly increased (p < 0.01) 2. Brain neurotransmitters (NA, DA, GLU) significantly increased (p < 0.01) 3. BDNF and NGF expression significantly increased (p < 0.05) 4. Intestinal peptide levels normalized (p < 0.05)	Enhanced vagal activity and neurotrophic signaling via microbiota–gut–brain axis modulation
Zhang et al. (2011)	D-galactose-induced brain aging rats	Liu Wei Dihuang 0.01 mL/g/day for 6 weeks	1. ACh level in visual cortex significantly increased (p < 0.001) 2. ChAT activity significantly increased (p < 0.001) 3. AChE activity significantly decreased (p < 0.001) 4. Neuronal density in hippocampus and visual cortex significantly increased (p < 0.001)	Restored cholinergic transmission and cognitive function by enhancing ACh synthesis and reducing AChE activity
Bai et al. (2019)	Bilateral carotid occlusion ischemia rats	Pei Yuan tong Nao 0.486 g/kg/day for 8 weeks	1. Escape latency significantly decreased (p < 0.05) 2. Hippocampal ACh level significantly increased (p < 0.05) 3. α7 nAChR expression significantly increased (p < 0.01)	Enhanced cognitive function by up-regulating ACh and α7 nAChR, improving cholinergic neurotransmission
Nakyam et al. (2023)	Metabolic syndrome + ischemic stroke rats	GCJ polyherbal 100–300 mg/kg/day for 42 days	1. AChE activity significantly decreased (p < 0.001) 2. eNOS expression significantly increased (p < 0.01) 3. BDNF and pERK/ERK ratio significantly increased (p < 0.001) 4. Neuronal density in hippocampus significantly increased (p < 0.01)	Improved memory and neural plasticity by suppressing AChE and enhancing BDNF-eNOS-ERK signaling
Jiang et al. (2013)	OVA + LPS-induced severe asthma mice	Qi’ao decoction 6.7–26.8 g/kg/day for 7 days	1. ACh-induced airway resistance significantly decreased (p < 0.01) 2. IL-4 level significantly decreased (p < 0.05) 3. Interferon-γ and IL-12 levels significantly increased (p < 0.05, p < 0.01)	Reduced ACh-mediated bronchoconstriction and restored Th1/Th2 cytokine balance in severe asthma
Gan et al. (2023)	Post-infectious cough mice and Guinea pigs	Shegan Zhike capsule 26–104 mg/kg/day for 7–14 days	1. Vagus-nerve-induced cough frequency significantly decreased (p < 0.01) 2. COX-2 level significantly decreased (p < 0.01) 3. PGE_2_ and thromboxane A_2_ (TXA_2_) levels significantly decreased (p < 0.01)	Inhibited vagal-mediated cough by suppressing COX-2/PGE_2_/TXA_2_ pathway and reducing airway inflammation
Wu et al. (2019)	Scopolamine-induced amnesia rats	Shenmayizhi decoction 3.3–16.5 g/kg/day for 4 weeks	1. AChE expression significantly decreased (p < 0.01) 2. ChAT expression significantly increased (p < 0.01) 3. Muscarinic M1 receptor expression significantly increased (p < 0.01)	Ameliorated memory impairment by enhancing hippocampal cholinergic function
Wu et al. (2015)	Abdominal aortic coarctation hypertension rats	Sini decoction 0.81 g/kg/day for 8 weeks	1. Systolic blood pressure significantly decreased (p < 0.001) 2. Baroreflex sensitivity significantly increased (p < 0.001)	Lowered arterial pressure and enhanced vagal baroreflex responsiveness to improve autonomic regulation
Qi (2014)	Clonidine-induced bradyarrhythmia rats	WenYangTongzhi 400–800 mg/kg (single dose)	1. Heart rate significantly increased (p < 0.05, p < 0.01) 2. Heart rate variability coefficient significantly decreased (p < 0.05) 3. Atrioventricular block incidence significantly decreased (p < 0.05)	Improved cardiac autonomic balance by increasing heart rate and reducing vagal over-excitation
Ma and Zhang (2016)	Coronary-ligation-induced heart failure rats	Yiqihuoxue compound + exercise training for 6 weeks	1. Plasma NE level significantly decreased (p < 0.01) 2. ACh level significantly increased (p < 0.01) 3. TH expression significantly decreased (p < 0.05) 4. ChAT expression significantly increased (p < 0.01)	Restored autonomic balance by reducing sympathetic activity and enhancing vagal tone
Du et al. (2006)	Low-protein forage anorexia rats	Yunpi prescription 37.6 g/kg/day for 3 weeks	1. Food intake significantly increased (p < 0.05) 2. Ventromedial hypothalamic neuron excitability significantly decreased (p < 0.05) 3. Required vagal stimulation intensity significantly increased (p < 0.05)	Reduced vagal–hypothalamic hyperexcitability and normalized central satiety feedback in anorexia
Tan et al. (2017)	Spleen-deficiency model rats	Fructus Aurantii decoction 0.104–0.416 g/mL for 7 days	1. ACh, GAS, MTL, and SP levels significantly increased (p < 0.01) 2. VIP level significantly decreased (p < 0.05)	Promoted gastrointestinal motility by enhancing excitatory peptides (ACh, GAS, MTL, SP) and reducing VIP.

ACh, acetylcholine; AChE, acetylcholinesterase; AQP5, aquaporin-5; BDNF, brain-derived neurotrophic factor; ChAT, choline acetyltransferase; CNTF, ciliary neurotrophic factor; COX-2, cyclooxygenase-2; eNOS, endothelial nitric oxide synthase; GAS, gastrin; HF, high-frequency power; HRV, heart rate variability; IL, interleukin; iNOS, inducible nitric oxide synthase; MAP, mean arterial pressure; M3R, muscarinic acetylcholine receptor 3; MTL, motilin; NE, norepinephrine; NGF, nerve growth factor; NO, nitric oxide; PGE_2_, prostaglandin E_2_; RMSSD, root mean square of successive differences; SDNN, standard deviation of NN, intervals; SP, substance P; TH, tyrosine hydroxylase; TRPA1, transient receptor potential ankyrin 1; VIP, vasoactive intestinal peptide; α7 nAChR, α7 nicotinic acetylcholine receptor.

#### 3.4.2 Single-herb extracts

Table 2 summarizes fifteen animal studies (Lu et al., 1984; Luo et al., 1996; Niijima et al., 1998; Medeiros et al., 2006; Shih et al., 2008; Zhao et al., 2010; Cui et al., 2012; Fu et al., 2013; El-Akabawy and El-Kholy, 2014; Wang et al., 2021; Zhong et al., 2022; Kim et al., 2023; Li et al., 2023; Asuku et al., 2024; Zhao et al., 2024) investigating single-herb preparations that modulate vagal or cholinergic function across cardiovascular, gastrointestinal, respiratory, neurological, and autonomic systems. Interventions included extracts of Zingiber officinale, Scutellaria baicalensis, Monochasma savatieri, Rhodiola sacra, and others. Across diverse disease models, these preparations enhanced parasympathetic or cholinergic signaling, as reflected by increased ACh and ChAT activity, reduced AChE, and upregulation of α7 nAChR, while suppressing sympathetic, serotonergic, or inflammatory mediators such as TH, 5-HT receptors, TNF-α, and IL-6. Cardiovascular and autonomic studies demonstrated muscarinic receptor–dependent hypotension and vagal bradycardia, whereas gastrointestinal models showed improved motility and mucosal protection via enhanced ACh-dependent secretion. Neurological and toxicological models exhibited restoration of cholinergic neurotransmission, reduced oxidative stress, and improved cognitive performance. Respiratory and systemic inflammatory models frequently activated the α7 nAChR–mediated cholinergic anti-inflammatory pathway. The regulatory effects were highly specific to the pathological context, for example, while extracts from Scutellaria baicalensis and Monochasma savatieri focused on suppressing lung inflammation via the α7 nAChR pathway, Zingiber officinale demonstrated a distinct role in gastrointestinal regulation by reducing vagal serotonergic signaling (5-HT3A/4 receptors) to alleviate chemotherapy-induced anorexia or normalizing cholinergic overactivation in motion sickness. Detailed study characteristics are provided in Supplementary Table S4.

**TABLE 2:** Summary of studies on vagal modulation by single herb extracts.

Study (Year)	Disease model	Intervention (dose, period)	Main vagal outcomes	Summary of findings
Niijima et al. (1998)	Normal anesthetized rats	Taste stimulation with *Pinellia*, *Zingiberis*, or 5:1 mixture (50 mg/mL, 10 min)	1. *Pinellia* significantly decreased vagal efferent activity (p < 0.05). 2. *Zingiberis* significantly increased activity (p < 0.01). 3. Glossopharyngeal section abolished effects	*Pinellia* suppressed and *Zingiberis* enhanced gastric vagal output via glossopharyngeal afferents
Lu et al. (1984)	Stress gastric ulcer rats	Raw or wine-stewed *Rheum palmatum* (0.075 g/100 g BW, 3 days pre-stress)	1. Stress increased cAMP/cGMP ratio (p < 0.001) 2. Wine-stewed *Rheum* normalized ratio (p < 0.01)	Wine-stewed *Rheum* restored autonomic balance via cyclic nucleotide regulation
Luo et al. (1996)	Normal anesthetized rats	*Medicago sativa* extract (0.05%, 1 mL, i.v. or i.c.v.)	1. Vagal discharge significantly increased peripherally and centrally (p < 0.001)	Alfalfa extract enhanced vagal efferent firing through dual central–peripheral mechanisms
Fu et al. (2013)	Stress gastric ulcer rats	Aloe extract (12 mL/kg, gavage × 7 days)	1. Gastric AChE-positive fibers decreased (p < 0.05) 2. Mucosal integrity preserved	Aloe reduced vagal cholinergic activity and protected gastric mucosa under stress
Zhao et al. (2010)	Scopolamine-induced amnesia mice	*Alpinia officinarum* extract (1.67–6.66 mg/kg, 10 days)	1. Brain ChAT significantly increased (p < 0.01) 2. TChE significantly decreased (p < 0.01)	*Alpinia* enhanced central cholinergic transmission and improved memory
Li et al. (2023)	Gastric cold syndrome mice	Galangal + ginger extracts (2.7–10.8 + 5 g/kg, 30 days)	1. Gastric ACh, GAS, MTL increased (p < 0.01) 2. IL-6, MDA decreased (p < 0.01)	Combination enhanced cholinergic and motility mediators while reducing inflammation
El-Akabawy and El-Kholy (2014)	Streptozotocin-induced diabetes rats	Ginger extract (500 mg/kg/day, 4–8 weeks)	1. AChE, TNF-α, iNOS, and caspase-3 decreased (p < 0.001) 2. Ki67 neurogenesis increased (p < 0.001)	Ginger restored cholinergic tone and reduced neuroinflammation in diabetic brain
Zhong et al. (2022)	Motion sickness mice	Ginger extract (100–500 mg/kg, single)	1. Plasma ACh decreased (−41%, p < 0.01) 2. Histamine decreased (−42%, p < 0.01)	Ginger normalized cholinergic and histaminergic overactivation in motion sickness
Kim et al. (2023)	Cisplatin-induced anorexia rats	Ginger extract (100/500 mg/kg, single + cisplatin 6 mg/kg)	1. Nodose 5-HT_3_A/5-HT_4_ receptor expression decreased (p < 0.001). 2. Food intake restored (p < 0.01)	Ginger reduced vagal serotonergic signaling to relieve cisplatin-induced anorexia
Zhao et al. (2024)	Lipopolysaccharide -induced lung injury mice	*Monochasma savatieri* extract (200–800 mg/kg, 7 days)	1. α7 nAChR upregulated, TLR4/NF-κB suppressed (p < 0.01) 2. Lung ACh increased (p < 0.01)	Extract activated α7 nAChR-mediated cholinergic anti-inflammatory pathway
Shih et al. (2008)	Normal anesthetized rats	*Rhodiola sacra* water fraction (10–75 mg/kg i.v.)	1. MAP decreased; HR increased (p < 0.05) 2. Effects blocked by hexamethonium/reserpine	*Rhodiola* induced hypotension via sympathetic inhibition and vagal activation
Cui et al. (2012)	Lipopolysaccharide-induced lung injury rats	*Scutellaria baicalensis* extract (1.5–3.0 g/kg, 7 days)	1. Serum ACh increased (p < 0.01) 2. TNF-α and NO decreased (p < 0.01)	Extract activated cholinergic anti-inflammatory pathway reducing TNF-α/NO.
Medeiros et al. (2006)	Normal normotensive rats	*Sida cordifolia* fraction (5–40 mg/kg i.v.)	1. MAP decreased 6%–33%, HR reduced 0%–80% (p < 0.001) 2. Effects blocked by atropine and vagotomy	*Sida* elicited muscarinic NO-dependent vasorelaxation and vagal bradycardia
Wang et al. (2021)	Scopolamine-induced amnesia mice	*Drynaria fortunei* (0.975 g/kg × 14 days) ± ER antagonist	1. Hippocampal ACh and ChAT increased, AChE decreased (p < 0.01) 2. ERβ activation essential	*Drynaria* improved cognition via ERβ-dependent cholinergic enhancement
Asuku et al. (2024)	HgCl_2_-induced neurotoxicity rats	6-Gingerol-rich *Zingiber officinale* (100/200 mg/kg × 14 days)	1. AChE, NO, TNF-α, NF-κB decreased (p < 0.05) 2. SOD, CAT, GPx increased (p < 0.05)	6-GIRIFZO restored cholinergic–antioxidant balance and prevented neuroinflammation

ACh, acetylcholine; AChE, acetylcholinesterase; CAT, catalase; ChAT, choline acetyltransferase; GPx, glutathione peroxidase; HR, heart rate; HRV, heart rate variability; IL, interleukin; iNOS, inducible nitric oxide synthase; MAP, mean arterial pressure; MDA, malondialdehyde; NF-κB, nuclear factor-κB; NO, nitric oxide; SOD, superoxide dismutase; TNF-α, tumor necrosis factor-α; α7 nAChR, α7 nicotinic acetylcholine receptor.

#### 3.4.3 Phytoconstituents

Twenty-nine preclinical studies (Jancsó and Such, 1983; Blackshaw et al., 2000; Wang et al., 2009; Ren et al., 2012; Wang et al., 2012; Zhang et al., 2012; Ai et al., 2013; Mei et al., 2013; Abu-Taweel, 2016; Song et al., 2016; Wu et al., 2017; Abdel-Diam et al., 2018; Ding et al., 2018; Dou et al., 2018; Lan et al., 2018; Lu et al., 2018; Xu et al., 2018; Akintunde et al., 2019; Dou et al., 2019; Bai et al., 2020; Keshk et al., 2020; Zaki et al., 2020; Fikry et al., 2022; Wang L. et al., 2022; Lim et al., 2023; Yang et al., 2023; Kaboutari et al., 2024; Öz and Erdal, 2024; Mulla et al., 2025) examined purified phytoconstituents targeting the vagal–cholinergic axis across neurological, ischemic, cardiovascular, respiratory, hepatic, inflammatory, metabolic, and toxicological models. Compounds such as curcumin, puerarin, breviscapine, trans-anethole, menthol, madecassoside, and Angelica sinensis polysaccharides consistently increased central or peripheral ACh, ChAT, vesicular ACh transporter expression, muscarinic receptor transcripts, or α7 nAChR, while decreasing AChE activity (p < 0.05–0.001). These biochemical changes were accompanied by improvements in cognitive and motor behavior, endothelial ACh-dependent vasodilation, lung mechanics, myocardial and cerebral injury indices, antioxidant capacity, and neurotrophic signaling, as well as reductions in lipid peroxidation, apoptosis, and inflammatory cytokines including TNF-α, IL-1β, IL-6, IL-17A, and IFN-γ. Mechanistic studies demonstrated that muscarinic or nicotinic receptor antagonism, α7 nAChR blockade, or cervical vagotomy abolished the observed biochemical and functional improvements, confirming dependence on vagal–cholinergic signaling. Foundational studies of capsaicin-sensitive vagal afferents further elucidated the sensory pathways through which several phytoconstituents exert their effects. Detailed experimental parameters are provided in Supplementary Table S5.

#### 3.4.4 Phytoconstituent derivatives and formulations

Six studies (Guo et al., 2018; Mohammed et al., 2020; Khadrawy et al., 2021; Noor et al., 2022; Shabbir et al., 2022; Ma et al., 2024) investigated chemically modified or nanoformulated phytoconstituents, including nanocurcumin, curcumin nanoparticles, tetrahydrocurcumin, and the mono-carbonyl curcumin analog COP-22. Across multiple disease models, these formulations produced more pronounced biochemical effects than their parent compounds, significantly decreasing AChE and increasing ACh and ChAT (p < 0.05–0.001). These changes were accompanied by normalization of Na^+^/K^+^-ATPase activity, restoration of monoaminergic balance, reductions in oxidative stress and inflammatory markers, and improvements in electrocardiographic parameters and spatial learning. Collectively, these findings suggest that optimized delivery systems enhance bioavailability and tissue distribution, thereby strengthening vagal cholinergic activation. Detailed results are provided in Supplementary Table S6.

#### 3.4.5 Essential oils

Three studies (Zhang et al., 2008; Komiya et al., 2009; Satou et al., 2022) evaluated essential oils derived from Atractylodes lancea, Pelargonium graveolens, and Lavandula angustifolia. Across rodent and canine models, essential oil administration consistently enhanced parasympathetic-vagal activity. Atractylodes lancea oil improved stress-induced delayed gastric emptying through vagal-dependent mechanisms involving motilin, gastrin, somatostatin, and hypothalamic corticotropin-releasing factor. Inhalation of Pelargonium graveolens oil reduced sympathetic activity and increased high-frequency parasympathetic power in awake mice. Topical application of Lavandula angustifolia oil produced modest reductions in heart rate and increases in high-frequency power in dogs, indicating transient vagal activation. Detailed study characteristics are provided in Supplementary Table S7.

## 4 Discussion

This systematic review provides the first comprehensive synthesis of experimental evidence examining the effects of herbal medicines and their phytoconstituents on vagus nerve activity. Across seventy-five *in vivo* studies, interventions ranging from multi-herb prescriptions and single-herb extracts to purified compounds, modified formulations, and essential oils consistently demonstrated modulation of vagal-related outcomes. Despite heterogeneity in study design, disease models, and outcome measures, the findings converge on a shared mechanistic framework involving enhancement of cholinergic signaling, activation of vagus-mediated anti-inflammatory pathways, and restoration of autonomic balance.

### 4.1 Summary of findings

The evidence indicates that both complex herbal formulations and isolated bioactive compounds can influence vagal function through multiple physiological and molecular processes. Multi-herb traditional prescriptions showed the broadest range of effects, encompassing improvements in ACh and ChAT, reduction of AChE, enhancement of HRV, and direct stimulation of vagal firing. Single-herb extracts and purified phytoconstituents yielded more targeted biochemical changes, particularly in cholinergic and inflammatory markers. Derivative formulations such as nanocurcumin and curcumin analogs demonstrated enhanced potency, likely due to improved pharmacokinetics and tissue distribution. Essential oils primarily modulated parasympathetic activity through sensory-autonomic integration, as evidenced by improvements in HRV. In summary, these results suggest that herbal-derived compounds can engage both peripheral and central components of the vagal network. The consistency of findings across independent studies and intervention types underscores the biological plausibility of herbal-induced vagal modulation.

Although the overall evidence is consistent, methodological quality was moderate. Risk-of-bias assessment revealed that half of the studies adequately described randomization, but allocation concealment and blinding were rarely reported. Most domains were rated as ‘unclear’ rather than ‘high’ risk, reflecting insufficient reporting and limiting reliable assessment of methodological quality. Studies measuring objective biochemical markers were less susceptible to bias compared with those using behavioral or histological endpoints. Recent publications showed gradual improvement in transparency, suggesting ongoing advancement in experimental rigor.

### 4.2 Mechanistic insights

Several mechanistic pathways emerged from this synthesis. At the biochemical level, herbal interventions commonly increased ACh availability by enhancing ChAT and inhibiting AChE. These effects collectively strengthen cholinergic neurotransmission and parasympathetic tone. At the molecular level, upregulation of the α7 nAChR and suppression of pro-inflammatory cytokines, including TNF-α, IL-6, and NF-κB, are consistent with the activation of the cholinergic anti-inflammatory pathway.

Electrophysiological and functional evidence further substantiated these biochemical findings. Several multi-herb prescriptions directly increased vagal efferent firing or improved baroreflex sensitivity, while essential oils shifted HRV toward parasympathetic dominance. In studies employing pharmacological blockade or cervical vagotomy, abolition of therapeutic effects confirmed dependence on intact vagal-cholinergic signaling. Beyond classical cholinergic pathways, emerging evidence suggests that autonomic regulation also involves non-canonical GPCR signaling and neuroimmune pathways implicated in vascular remodeling and tissue repair. These broader mechanisms may contribute to the cardiovascular effects of herbal interventions and represent promising targets for future mechanistic studies (Zhong et al., 2025; Yao et al., 2026).

Despite considerable heterogeneity in intervention type, many studies demonstrated convergence on common downstream cholinergic pathways involving acetylcholine signaling, acetylcholinesterase inhibition, α7 nicotinic acetylcholine receptor activation, and modulation of inflammatory mediators. Representative examples of active compounds identified in the included studies included polyphenols (e.g., curcumin), vanilloids (e.g., capsaicin), terpenoids (e.g., borneol and menthol), flavonoids (e.g., baicalin), and polysaccharides (e.g., Angelica sinensis polysaccharides). Despite these diverse chemical structures, the mechanisms by which chemically distinct compounds modulate vagal and cholinergic pathways remain incompletely understood. Indirect neuroimmune modulation, synergistic effects, off-target interactions, and pathway convergence may all contribute to the observed findings. Consequently, direct structure-activity relationships cannot be established based on the currently available evidence and warrant further investigation in future mechanistic studies.

An additional consideration when interpreting these mechanistic findings is the bioavailability and tissue distribution of herbal compounds. Curcumin, one of the most extensively investigated phytochemicals in the included studies, exhibits low oral bioavailability because of poor aqueous solubility and extensive first-pass metabolism. Consequently, observed *in vivo* effects may reflect the activity of metabolites, local gastrointestinal effects, modulation of gut barrier function, or indirect neuroimmune interactions rather than direct cholinergic activation alone. Similarly, large molecules such as *Angelica sinensis* polysaccharides have limited blood–brain barrier permeability, suggesting that reported central nervous system effects may arise from peripheral immune modulation and subsequent vagal signaling rather than direct central target engagement. These pharmacokinetic considerations highlight the need for future studies integrating pharmacodynamic and pharmacokinetic approaches to better understand the mechanisms and translational potential of herbal vagal modulators.

### 4.3 Implications for research and practice

The vagus nerve represents a central regulatory hub linking neural, immune, and metabolic systems. The demonstration that herbal medicines can influence this axis expands the mechanistic foundation for traditional therapies in managing inflammatory, cardiovascular, gastrointestinal, and neurodegenerative conditions. The consistent modulation of acetylcholine-related pathways aligns with clinical observations that herbal interventions often improve stress resilience, heart-rate variability, and systemic inflammation.

These findings also complement the growing field of bioelectronic medicine by highlighting pharmacological alternatives to invasive vagus nerve stimulation. Herbal compounds with proven vagal-enhancing effects, such as curcumin, puerarin, and ginger constituents, may provide a pharmacological basis for developing non-invasive strategies that could potentially complement or, in specific preclinical contexts, offer an alternative approach to invasive VNS. Nevertheless, vagal activation is not universally beneficial, and excessive cholinergic stimulation may theoretically result in adverse physiological effects including bradycardia, bronchoconstriction, hypotension, or gastrointestinal hypermotility. Given that several interventions in the present review increased ACh levels and reduced AChE activity, future translational studies should carefully evaluate dose-response relationships, therapeutic windows, and potential adverse effects associated with excessive vagal activation.

Another important consideration is the level of phytochemical definition of the investigated interventions. While several studies evaluated purified phytoconstituents or structurally characterized compounds such as curcumin, capsaicin, puerarin, madecassoside, and menthol, most studies employed crude herbal extracts or multi-herb formulations containing numerous potentially active constituents. Although this approach aligns closely with traditional medicine practice, it limits identification of the specific chemical mediators responsible for the observed biological effects and precludes differentiation between synergistic, additive, or off-target mechanisms. Future studies should prioritize the investigation of isolated, structurally characterized compounds alongside standardized extracts to facilitate mechanistic elucidation, improve reproducibility, and support translational drug development.

Future studies should adopt standardized experimental designs with clear randomization, allocation concealment, and blinding protocols. Integration of electrophysiological, biochemical, and functional outcomes within the same experimental model would enable stronger causal inference. Dose–response analyses, phytochemical characterization, pharmacokinetic profiling, and comparison of herbal combinations versus isolated compounds are needed to clarify synergistic mechanisms. Furthermore, translation of these preclinical findings into human trials will be essential to confirm whether the observed vagal modulation translates into therapeutic benefit across clinical populations.

### 4.4 Limitations

Several limitations of this systematic review should be considered. First, heterogeneity among *in vivo* models including differences in species, disease models, experimental protocols, outcome measures, and intervention durations limited direct comparison across studies and precluded quantitative meta-analysis. Although several studies demonstrated similar directional effects, such as increased ACh levels or reduced AChE activity, these findings should not be interpreted as indicating comparable effect sizes or magnitudes of vagal modulation across different experimental models, interventions, or outcome measures. Furthermore, since these findings are derived from animal models, significant interspecies differences in vagal anatomy and neuro-inflammatory responses must be acknowledged, which may limit the direct extrapolation of these results to human clinical settings.

Second, the overall quality of the included studies was variable. Many *in vivo* VNS studies employed small sample sizes, short stimulation or follow-up periods, and incomplete reporting of randomization, blinding, or allocation concealment. In addition, several studies relied on surrogate biochemical or molecular markers to infer vagal or cholinergic activity rather than direct functional assessments of vagal nerve engagement, which may affect the strength of mechanistic inferences. It should also be noted that many studies utilized invasive administration routes, such as intraperitoneal or intravenous injections, which may bypass natural physiological barriers and differ from the pharmacological profiles of oral herbal intake.

Third, although comprehensive searches were conducted using multiple databases and predefined keywords, relevant studies may have been missed due to inconsistent terminology or incomplete indexing. VNS studies that did not explicitly reference “vagal nerve stimulation,” “VNS,” or related cholinergic or inflammatory pathways in titles or abstracts may not have been captured. Additionally, variability in reporting stimulation parameters and outcomes may have limited study identification. Moreover, the potential for publication bias cannot be ruled out, as studies with positive vagal outcomes are more likely to be published than those showing no significant effect.

Another important limitation relates to the incomplete and heterogeneous reporting of phytochemical characterization across included interventions. Herbal preparations varied substantially with respect to botanical source, plant part used, extraction procedures, constituent composition, and analytical characterization. Although some studies employed chromatographic methods such as high-performance liquid chromatography, liquid chromatography-mass spectrometry, or gas chromatography-mass spectrometry to quantify major constituents, many studies reported only crude drug doses or intervention names without detailed compositional information. Such variability limits reproducibility, complicates dose comparisons across studies, and reduces confidence in mechanistic interpretation of vagal modulation and cholinergic signaling. Detailed information regarding botanical source, extraction methods, phytochemical characterization, and analytical procedures is provided in Supplementary Tables S8–S12 according to intervention type. Accordingly, the mechanistic conclusions of the present review should be considered preliminary pending further chemical characterization and validation using structurally identified compounds.

Another source of uncertainty relates to the chemical stability of herbal preparations during storage and administration. Although some studies reported storage conditions such as refrigeration, freeze-drying, desiccator storage, or protection from light, none reported post-storage verification of phytochemical composition or formal stability testing before administration. Consequently, degradation of active constituents during storage may have contributed to variability in the effective dose delivered across studies and may partially explain differences in reported efficacy.

The limited reporting of botanical authentication represents an additional challenge in interpreting the current evidence base. Although some studies reported voucher specimens, expert morphological identification, pharmacognostic verification, or chromatographic fingerprinting, botanical authentication procedures were reported in only 15 of 75 included studies (20.0%). The remaining studies did not provide sufficient information regarding independent verification of plant identity, creating the potential for species misidentification, substitution, or adulteration and contributing to variability in phytochemical composition and biological activity. This issue may be particularly relevant for studies utilizing commercially sourced plant materials without independent verification. Detailed information regarding botanical authentication procedures is provided in Supplementary Tables S8–S12.

An additional challenge relates to the lack of standardized dose reporting across studies. While purified phytoconstituents were generally reported as individual compound doses, many studies employed crude extracts or multi-herb formulations for which the concentrations of biologically active constituents were incompletely characterized or unknown. Consequently, direct dose comparisons across intervention types were often not possible, limiting interpretation of dose-response relationships and reducing comparability across studies. Future studies would benefit from standardized reporting of both administered dose and quantified active constituent content to improve reproducibility and facilitate quantitative synthesis.

Despite these limitations, this systematic review provides a comprehensive synthesis of available *in vivo* evidence and identifies consistent patterns supporting the role of vagal nerve stimulation in modulating physiological and inflammatory responses, while highlighting important gaps for future standardized and translational research.

## 5 Conclusion

This review provides convergent preclinical evidence that herbal medicines and their phytoconstituents can enhance vagal activity through cholinergic and anti-inflammatory mechanisms. Although methodological limitations and the inherent gaps between animal models and human physiology persist, the overall consistency of the findings highlights the vagus nerve as a key mediator of herbal therapeutic effects. Given these findings, further well-designed experimental and clinical studies are required to confirm these mechanisms in human subjects and to evaluate the clinical feasibility of herbal-induced vagal modulation as a potential complementary therapeutic strategy for autonomic and inflammatory disorders. Particular attention should be paid to differences in administration routes, as oral bioavailability and first-pass metabolism may substantially alter the vagal effects observed following parenteral administration in animal models. The current evidence should therefore be viewed primarily as a preclinical foundation for future mechanistic and clinical investigations rather than direct evidence supporting clinical efficacy.
