# Supplementary material for: Effects of herbal medicines and their phytoconstituents on vagus nerve stimulation: a systematic review of in vivo studies
Source: Front Pharmacol. 2026 Aug 12;17:1866222. doi: 10.3389/fphar.2026.1866222 (PMC13507635; doi:10.3389/fphar.2026.1866222)
Supplement: Supplementary file 1 [file Table1.pdf]

Supplementary Table 1. Search strategies for each database

Databases	Search strategy
**PubMed**	("Herbal Medicine"[Mesh] OR "Herbal Drugs"[Mesh] OR "Phytotherapy"[Mesh] OR herb*[tiab] OR phytochemical*[tiab] OR "plant extract*"[tiab] OR "medicinal plant*"[tiab] OR "natural product*"[tiab] OR botanical*[tiab]) AND ("Vagus Nerve Stimulation"[Mesh] OR "vagal nerve stimulation"[tiab] OR "vagus nerve"[tiab] OR "vagal stimulation"[tiab] OR VNS[tiab] OR "vagal modulation"[tiab] OR "vagal tone"[tiab] OR "vagal reflex"[tiab] OR "parasympathetic stimulation"[tiab] OR "parasympathetic activation"[tiab] OR “autonomic nervous system” [tiab] OR “autonomic” [tiab] OR "cholinergic anti-inflammatory pathway"[tiab] OR "acetylcholine"[tiab] OR "choline acetyltransferase"[tiab] OR "acetylcholinesterase"[tiab] OR "baroreflex"[tiab] OR "baroreflex sensitivity"[tiab] OR "heart rate variability"[tiab]) AND ("Animal Experimentation"[Mesh] OR "Animals"[Mesh] OR "animal model*"[tiab] OR "animal stud*"[tiab] OR "in vivo"[tiab] OR preclinical[tiab] OR "pre-clinical"[tiab] OR rat*[tiab] OR mouse[tiab] OR mice[tiab] OR rabbit*[tiab] OR "guinea pig*"[tiab] OR cat*[tiab] OR dog*[tiab] OR rodent*[tiab])
**Embase**	(‘herbal medicine’/exp OR ‘herbal drug’/exp OR ‘phytotherapy’/exp OR herb*:ti,ab OR phytochemical*:ti,ab OR ‘plant extract*’:ti,ab OR ‘medicinal plant*’:ti,ab OR ‘natural product*’:ti,ab OR botanical*:ti,ab) AND (‘vagus nerve stimulation’/exp OR ‘vagus nerve’:ti,ab OR ‘vagal stimulation’:ti,ab OR ‘vagal nerve stimulation’:ti,ab OR VNS:ti,ab OR ‘vagal modulation’:ti,ab OR ‘vagal tone’:ti,ab OR ‘vagal reflex’:ti,ab OR ‘parasympathetic stimulation’:ti,ab OR ‘parasympathetic activation’:ti,ab OR ‘autonomic nervous system’:ti,ab OR ‘autonomic’ :ti,ab OR ‘cholinergic anti-inflammatory pathway’:ti,ab OR ‘acetylcholine’:ti,ab OR ‘choline acetyltransferase’:ti,ab OR ‘acetylcholinesterase’:ti,ab OR ‘baroreflex’:ti,ab OR ‘baroreflex sensitivity’:ti,ab OR ‘heart rate variability’:ti,ab) AND (‘animal experiment’/exp OR ‘animal model’/exp OR ‘preclinical study’/exp OR ‘in vivo study’/exp OR ‘animal model*’:ti,ab OR ‘animal stud*’:ti,ab OR ‘in vivo’:ti,ab OR preclinical:ti,ab OR ‘pre-clinical’:ti,ab OR rat*:ti,ab OR mouse:ti,ab OR mice:ti,ab OR rabbit*:ti,ab OR ‘guinea pig*’:ti,ab OR cat*:ti,ab OR dog*:ti,ab OR rodent*:ti,ab)
**Cochrane Library**	#1 ("herbal medicine" OR "herbal drug" OR "phytotherapy" OR herb* OR phytochemical* OR "plant extract*" OR "medicinal plant*" OR "natural product*" OR botanical*)<br>#2 ("vagus nerve stimulation" OR "vagal nerve stimulation" OR "vagus nerve" OR "vagal stimulation" OR "vagal tone" OR "vagal modulation" OR "vagal reflex" OR VNS OR "parasympathetic stimulation" OR "parasympathetic activation" OR “autonomic nervous system” OR “autonomic” OR "cholinergic anti-inflammatory pathway" OR "acetylcholine" OR "choline acetyltransferase" OR "acetylcholinesterase" OR "baroreflex" OR "baroreflex sensitivity" OR "heart rate variability")<br>#3 ("animal model*" OR "animal stud*" OR "in vivo" OR "preclinical" OR "pre-clinical" OR rat* OR mouse OR mice OR rabbit* OR "guinea pig*" OR cat* OR dog* OR rodent*)<br>#4 #1 AND #2 AND #3
**ScienceDirect**	(herb* OR phytochemical* OR "plant extract*" OR "medicinal plant*" OR "natural product*" OR botanical*) AND ("vagal nerve stimulation" OR "vagus nerve" OR "vagal stimulation" OR VNS OR "vagal modulation" OR "vagal tone" OR "vagal reflex" OR "parasympathetic stimulation" OR "parasympathetic activation" OR “autonomic nervous system” OR “autonomic” OR "cholinergic anti-inflammatory pathway" OR "acetylcholine" OR "choline acetyltransferase" OR "acetylcholinesterase" OR "baroreflex" OR "baroreflex sensitivity" OR "heart rate variability") AND ("animal model*" OR "animal stud*" OR "in vivo" OR preclinical OR "pre-clinical" OR rat* OR mouse OR mice OR rabbit* OR "guinea pig*" OR cat* OR dog* OR rodent*)
**OASIS**	(허브 의약품 OR 식물 추출물 OR 약용 식물 OR 자연 제품 OR 식물 화합물 OR 식물성 OR 한약 OR 한약재 OR 한약 추출물 OR 한방 치료 OR 한의약 OR 약초 OR 본초 OR 한방 제제) AND (미주 신경 자극 OR 미주 신경 OR 미주 신경 자극 효과 OR 미주 신경 조절 OR 부교감 신경 자극 OR 부교감 신경 활성화) AND (동물 모델 OR 동물 실험 OR 생체 실험 OR 전임상 연구 OR 쥐 OR 마우스 OR 토끼 OR 기니피그 OR 설치류)
**CNKI Wanfang**	(中药 + 中草药 + 草药 + 植物提取物 + 药用植物 + 天然产物 + 植物化学成分) AND (迷走神经刺激 + 迷走神经调节 +VNS+ 迷走神经活动 + 副交感神经激活 + 迷走神经反射 + 第十脑神经) AND (动物实验 + 动物模型 + 体内研究 + 前临床研究 + 大鼠 + 小鼠 + 兔子 + 豚鼠 + 猫 + 狗 + 啮齿动物)
**CiNii**	(ハーブ OR 薬用植物 OR 生薬 OR 漢方薬 OR 植物抽出物 OR 天然物) AND (迷走神経刺激 OR 迷走神経 OR VNS OR 副交感神経刺激) AND (動物実験 OR 動物モデル OR in vivo OR 前臨床研究 OR ラット OR マウス OR 兎 OR モルモット)

Supplementary Table 2. Risk of bias assessment to each study in each of the SYRCLE items

Study	D1	D2	D3	D4	D5	D6	D7	D8	D9	D10
Abdel-Diam 2018	Unclear	Low	Unclear	Unclear	Unclear	Unclear	Unclear	Low	Low	Unclear
Abu-Taweel 2016	Unclear	Low	Unclear	Unclear	Unclear	Unclear	Unclear	Low	Low	Unclear
Ai 2013	Unclear	Unclear	Unclear	Unclear	Unclear	Unclear	Unclear	Unclear	Low	High
Akintunde 2019	Unclear	Low	Unclear	Unclear	Unclear	Unclear	Unclear	Low	Low	Unclear
Asuku 2024	Unclear	Low	Unclear	Unclear	Unclear	Unclear	Unclear	Low	Low	Unclear
Bai 2019	Low	Low	Unclear	Unclear	Unclear	Unclear	Unclear	Low	Low	High
Bai 2020	Unclear	Low	Unclear	Unclear	Unclear	Unclear	Unclear	Low	Low	Low
Blackshaw 2000	Unclear	Unclear	Unclear	Unclear	Unclear	Unclear	Unclear	Low	Unclear	Unclear
Cui 2012	Unclear	Low	High	Unclear	Unclear	Unclear	Unclear	Low	Low	Unclear
Ding 2018	Unclear	Low	Unclear	Unclear	Unclear	Unclear	Unclear	Low	Low	Unclear
Dou 2018	Low	Low	Unclear	Unclear	Unclear	Unclear	Low	Low	Low	Low
Dou 2019	Unclear	Low	Unclear	Unclear	Unclear	Unclear	Unclear	Low	Low	Unclear
Du 2006	Unclear	Low	Unclear	Unclear	Unclear	Unclear	Unclear	Low	Low	Unclear
Du 2007	Unclear	Low	Unclear	Unclear	Unclear	Unclear	Unclear	Low	Low	Unclear
El-Akabawy 2014	Unclear	Low	Unclear	Unclear	Unclear	Unclear	Unclear	Low	Low	Unclear
Fikry 2022	Low	Low	Unclear	Unclear	Unclear	Unclear	Unclear	Low	Low	Unclear
Fu 2013	Unclear	Low	Unclear	Unclear	Unclear	Unclear	Unclear	Low	Low	Unclear
Gan 2023	Unclear	Low	Unclear	Unclear	Unclear	Unclear	Unclear	Low	Low	Unclear
Guo 2018	Low	Low	Unclear	Unclear	Unclear	Unclear	Unclear	Low	Low	Unclear
Jansco 1983	Unclear	Low	Unclear	Unclear	Unclear	Unclear	Unclear	Low	Unclear	Unclear
Jiang 2013	Low	Low	Unclear	Unclear	Unclear	Unclear	Unclear	Low	Low	Unclear
Jiang 2016	Low	Low	Unclear	Unclear	Unclear	Unclear	Unclear	High	Low	Unclear
Kaboutari 2024	Low	Low	Unclear	Unclear	Unclear	Unclear	Unclear	Low	Low	Unclear
Keshk 2020	Unclear	Low	Unclear	Unclear	Unclear	Unclear	Unclear	Low	Low	Unclear
Khadrawy 2021	Unclear	Low	Unclear	Unclear	Unclear	Unclear	Unclear	Low	Low	Unclear
Kim 2023	Low	Low	Unclear	Unclear	Unclear	Unclear	Unclear	Low	Low	Unclear
Komiya 2009	Low	Low	Unclear	Low	Unclear	Low	Unclear	Low	Low	Low
Lan 2018	Unclear	Low	Unclear	Unclear	Unclear	Unclear	Unclear	Low	Low	Unclear
Li 2014	Low	Low	Unclear	Unclear	Unclear	Unclear	Unclear	Low	Low	Unclear
Li 2015	Low	Low	Unclear	Unclear	Unclear	Unclear	Unclear	Low	Low	Unclear
Li 2023	Low	Low	Unclear	Unclear	Unclear	Unclear	Unclear	Low	Low	Unclear
Lim 2023	Low	Low	Unclear	Unclear	Unclear	Unclear	Unclear	Low	Low	Unclear
Lu 1984	Unclear	Low	Unclear	Unclear	Unclear	Unclear	Unclear	Low	Low	Unclear
Lu 2015	Low	Low	Unclear	Unclear	Unclear	Unclear	Unclear	Low	Low	Unclear
Lu 2018	Low	Low	Unclear	Unclear	Unclear	Unclear	Unclear	Low	Low	Unclear
Luo 1996	Unclear	Unclear	Unclear	Unclear	Unclear	Unclear	Unclear	Unclear	Unclear	Unclear
Ma 2016	Low	Low	Unclear	Unclear	Unclear	Unclear	Unclear	Low	Low	Unclear
Ma 2024	Low	Low	Unclear	Unclear	Unclear	Unclear	Unclear	Low	Low	Low
Medeiros 2006	Unclear	Low	Unclear	Unclear	Unclear	Unclear	Unclear	Low	Low	Unclear
Mei 2013	Unclear	Low	Unclear	Unclear	Unclear	Unclear	Unclear	Low	Low	Unclear
Mohammed 2020	Low	Low	Unclear	Unclear	Unclear	Unclear	Unclear	Low	Low	Low
Mulla 2025	Low	Low	Unclear	Unclear	Unclear	Unclear	Unclear	Low	Low	Low
Nakyam 2023	Low	Low	Unclear	Unclear	Unclear	Unclear	Unclear	Low	Low	Low
Niijima 1998	Unclear	Low	Unclear	Unclear	Unclear	Unclear	Unclear	Low	Low	Unclear

Noor 2022	Low	Low	Unclear	Unclear	Unclear	Unclear	Unclear	Low	Low	Low
Oz 2024	Low	Low	Unclear	Unclear	Unclear	Unclear	Unclear	Low	Low	Low
Qi 2014	Unclear	Low	Unclear	Unclear	Unclear	Unclear	Unclear	Low	Low	Unclear
Ren 2012	Low	Low	Unclear	Unclear	Unclear	Unclear	Unclear	Low	Low	Unclear
Satou 2022	Low	Low	Unclear	Unclear	Unclear	Unclear	Unclear	Low	Low	Low
Shabbir 2022	Low	Low	Unclear	Unclear	Unclear	Unclear	Unclear	Low	Low	Low
Shih 2008	Unclear	Low	Unclear	Unclear	Unclear	Unclear	Unclear	Low	Low	Unclear
Song 2016	Low	Low	Unclear	Unclear	Unclear	Unclear	Unclear	Low	Low	Low
Sun 2022	Low	Low	Unclear	Unclear	Unclear	Unclear	Unclear	Low	Low	Low
Sun 2025	Low	Low	Unclear	Unclear	Unclear	Unclear	Unclear	Low	Low	Unclear
Tan 2017	Unclear	Low	Unclear	Unclear	Unclear	Unclear	Unclear	Low	Low	Unclear
Wang 2009	Unclear	Low	Unclear	Unclear	Unclear	Unclear	Unclear	Low	Low	Unclear
Wang 2011	Low	Low	Unclear	Unclear	Unclear	Unclear	Unclear	Low	Low	Low
Wang 2012	Unclear	Unclear	Unclear	Unclear	Unclear	Unclear	High	High	Unclear	Unclear
Wang 2021	Low	Low	Unclear	Unclear	Unclear	Unclear	Unclear	Low	Low	Unclear
Wang 2022	Unclear	Unclear	Unclear	Unclear	Unclear	Unclear	Unclear	High	Unclear	Unclear
Wu 2015	Unclear	Low	Unclear	Unclear	Unclear	Unclear	Unclear	High	Unclear	Unclear
Wu 2017	Low	Low	Unclear	Unclear	Unclear	Unclear	Unclear	Low	Low	Unclear
Wu 2019	Low	Low	Unclear	Unclear	Unclear	Unclear	Unclear	Low	Low	Unclear
Xie 2015	Low	Low	Unclear	Unclear	Unclear	Unclear	Unclear	Low	Low	Unclear
Xu 2018	Low	Low	Unclear	Unclear	Unclear	Unclear	Unclear	Low	Low	Unclear
Yang 2023	Low	Low	Unclear	Unclear	Unclear	Unclear	Unclear	Low	Low	Unclear
Zaki 2020	Unclear	Low	Unclear	Unclear	Unclear	Unclear	Unclear	Low	Low	Unclear
Zhang 2005	Unclear	Low	Unclear	Unclear	Unclear	Unclear	Unclear	Low	Unclear	Unclear
Zhang 2005b	Unclear	Low	Unclear	Unclear	Unclear	Unclear	Unclear	Low	Unclear	Unclear
Zhang 2008	Unclear	Low	Unclear	Unclear	Unclear	Unclear	Unclear	Low	Unclear	Unclear
Zhang 2011	Low	Low	Unclear	Unclear	Unclear	Unclear	Unclear	Low	Low	Unclear
Zhang 2012	Low	Low	Unclear	Unclear	Unclear	Unclear	Unclear	Low	Low	Unclear
Zhao 2010	Unclear	Low	Unclear	Unclear	Unclear	Unclear	Unclear	Low	Low	Unclear
Zhao 2024	Unclear	Low	Unclear	Unclear	Unclear	Unclear	Unclear	Low	Unclear	Unclear
Zhong 2022	Low	Low	Unclear	Unclear	Unclear	Unclear	Unclear	Low	Low	Unclear

Supplementary Table 3. Summary of studies on vagal modulation by multi-herb prescriptions

Study (Year)	Disease Model	Subjects	Intervention	Main Vagal Outcomes	Summary of findings
Wang 2011[1]	Spontaneously hypertensive rat (SHR) model of hypertension	54 male SHR (6 weeks old), 18 Wistar-Kyoto rats as controls	Banxia Baizhu Tianma Decoction (BBTD) 4.32 g/kg/day by gavage, 7 – 24 weeks; observed until 32 weeks. Captopril 3.375 g/kg/day as positive control.	1. Acetylcholine (ACh)-dependent relaxation of superior mesenteric artery significantly increased at 24 weeks ($P < 0.01$).<br>2. Serum nitric oxide (NO_2^-) concentration significantly decreased at 18 and 24 weeks ($P < 0.01$).<br>3. Total antioxidant capacity significantly increased at 18 weeks ($P < 0.01$).<br>4. Interleukin-1 (IL-1) mRNA expression significantly decreased at 18, 24, and 32 weeks ($P < 0.05$).<br>5. Interleukin-6 (IL-6) and inducible nitric oxide synthase (iNOS) mRNA expression significantly decreased at 32 weeks ($P < 0.01$).<br>6. Arterial pressure significantly decreased at 18 and 24 weeks ($P < 0.05$).	BBTD significantly improved acetylcholine-mediated endothelial relaxation and reduced oxidative and inflammatory markers by suppressing iNOS and IL-1 expression, enhancing endothelial nitric oxide regulation and antioxidant capacity.
Lu 2015[2]	NOD mouse model of primary Sjögren's syndrome (pSS)	18 female NOD/Ltj mice (8–9 weeks), plus 6 female BALB/c mice as normal controls	Banxia Qinlian Decoction (BQD) 60 g/kg/day by gavage for 12 weeks; positive control = Tripterygium glycosides 10 mg/kg/day	1. Interleukin-17 (IL-17) mRNA and protein expression significantly decreased in parotid and submandibular glands ($P < 0.01$).<br>2. Interleukin-6 (IL-6) mRNA and protein expression significantly decreased ($P < 0.01$).<br>3. Type 3 muscarinic acetylcholine receptor (M3R) mRNA and protein expression significantly decreased ($P < 0.05$, $P < 0.01$).<br>4. Aquaporin-5 (AQP5) mRNA and protein expression significantly decreased ($P < 0.05$, $P < 0.01$).<br>5. Compared with controls, untreated model mice showed significant up-regulation of IL-17, IL-6, M3R, and AQP5 ($P < 0.01$).	BQD significantly downregulated IL-17/IL-6 and normalized muscarinic receptor 3 (M3R) and aquaporin-5 (AQP5) expression, indicating suppression of Th17/IL-17-mediated immune inflammation and protection against cholinergic neurotoxicity in exocrine glands.
Zhang 2005[3]	Water-immersion restraint stress–induced gastric ulcer in rats	90 male Wistar rats (200–250 g) divided into 9 groups: normal, model, "Xin-kai" (acrid-opening), "Ku-jiang" (bitter-descending), "Gan-bu" (sweet-tonifying), combined subformulas, and full Banxia Xiexin Decoction (BXD)	Banxia Xiexin Decoction (BXD) and its subformulas, decocted and administered 3 mL/rat twice daily for 3 days (dose ≈ 10× clinical equivalent)	1. Choline acetyltransferase (ChAT) expression in brain significantly increased in model rats vs. normal ($P < 0.01$).<br>2. BXD and all component formula groups significantly decreased brain ChAT expression vs. model ($P < 0.01$).<br>3. Gastric mucosal ChAT expression significantly increased in model group ($P < 0.01$) and significantly decreased in BXD, Gan-bu, Ku-jiang, and combination groups ($P < 0.01$).<br>4. Gastric mucosal integrity markedly improved, with hemorrhage and necrosis reduced in treated rats.	BXD and its subformulas reduced excessive cholinergic (vagal) excitability by downregulating ChAT expression in brain and stomach, restoring autonomic balance and protecting gastric mucosa under stress.
Du 2007[4]	Juvenile anorexia model in rats (hypothalamic feeding regulation disorder)	51 juvenile rats (3 groups: normal, anorectic model, Baobaole-treated)	Baobaole oral liquid (compound spleen-activating formula) by gavage once daily for 3 weeks; compared to saline control	1. Lateral hypothalamic area (LHA) neuron excitatory response to gastric vagus stimulation significantly prolonged in model group ($P < 0.01$).<br>2. Ventromedial hypothalamic nucleus (VMN) inhibitory neuron response duration significantly increased in model group ($P < 0.01$).<br>3. Required stimulation intensity for vagal activation significantly reduced in model group ($P < 0.01$).<br>4. Baobaole treatment normalized LHA and VMN neuron excitability and response duration ($P < 0.01$).<br>5. Number of glucose-sensitive neurons in LHA decreased and in VMN increased in model group ($P < 0.01$); both restored to normal after treatment.	Baobaole oral liquid modulated vagal afferent–hypothalamic signaling by restoring balance between excitatory LHA and inhibitory VMN neuronal activity, normalizing glucose sensitivity and appetite regulation in anorectic rats.

Xie 2015[5]	Multiple organ dysfunction syndrome (MODS) induced by Escherichia coli peritonitis	50 Wistar rats (male and female, 200–250 g), divided into control (n = 10), MODS (n = 20), and Dachengqi Decoction (DCQD)–treated (n = 20) groups	Dachengqi Decoction (DCQD) 2 mL/rat, crude drug = 1 g/mL, gavage twice daily for 2 days before E. coli injection; observed 24 h post-injection	1. Acetylcholine (ACh) nerve fiber quantity and fluorescence integraloptical density (IOD) in intestinal plexus significantly decreased in MODS group (P < 0.01) and significantly increased after DCQD treatment (P < 0.01).<br>2. Substance P (SP) nerve fiber quantity and IOD significantly decreased in MODS group (P < 0.01) and significantly increased with DCQD (P < 0.01).<br>3. Vasoactive intestinal peptide (VIP) nerve fiber quantity and IOD significantly decreased in MODS group (P < 0.01) and significantly increased after DCQD (P < 0.01).<br>4. Nitric oxide (NO, measured via neuronal nitric oxide synthase [nNOS]) nerve fiber quantity and IOD significantly decreased in MODS group (P < 0.01) and significantly increased with DCQD (P < 0.01).<br>5. Enteric nerve network disruption in MODS rats was markedly restored by DCQD.	Dachengqi Decoction significantly protected and repaired cholinergic (ACh, SP) and inhibitory (VIP, NO) enteric neurons, restoring enteric neural network integrity and promoting gastrointestinal motility in MODS rats.
Zhang 2005[6]	Anorexia model induced by special low-protein forage in rats	36 SD rats (35–40 days old, 60 ± 10 g), divided into normal, model, and Er'bao-treated groups (n = 12 each)	Er'bao Granule 3.76 g/100 g/day (crude drug, 2× clinical equivalent), gavage for 3 weeks after 1 week of modeling	1. Food intake significantly decreased in model group (P < 0.01) and returned to normal after Er'bao treatment (P > 0.05).<br>2. Proportion of lateral hypothalamic area (LHA) neurons responding to gastric vagus stimulation not significantly altered among groups (P > 0.05).<br>3. Proportion of glucose-sensitive neurons among LHA neurons responding to vagal input significantly decreased in model rats (P < 0.01) and normalized after Er'bao treatment (P > 0.05).<br>4. No significant difference in non–vagal-responsive neurons among groups (P > 0.05).	Er'bao Granule significantly restored glucose sensitivity of LHA neurons integrating vagal afferent and glycemic signals, rebalancing central appetite regulation in anorectic rats.
Jiang 2016[7]	6-Hydroxydopamine (6-OHDA)–induced cardiac sympathetic denervation in rats	54 male Wistar rats (8 weeks old, 250–300 g), divided into control, model, methylcobalamin, and Guizhi Decoction (GZD) groups (ratios 2:1, 1:2, 1:1; n = 9 each)	Guizhi Decoction with Ramulus Cinnamomi (RC): Radix Paeoniae Alba (RPA) ratios of 2:1, 1:2, or 1:1 (4.0–5.5 g crude drug/kg/day), gavage for 10 days; methylcobalamin 0.15 mg/kg/day as control	1. Norepinephrine (NE) and tyrosine hydroxylase (TH) levels significantly decreased in model rats (P < 0.01) and significantly increased after GZD treatment (P < 0.01).<br>2. Choline acetyltransferase (ChAT) unchanged among groups (P > 0.05).<br>3. TH/ChAT ratio significantly increased in GZD 2:1 and 1:1 groups (P < 0.01), indicating improved sympathetic–vagal balance.<br>4. Nerve growth factor (NGF) significantly increased in model rats (P < 0.01) and significantly decreased after GZD 1:1 treatment (P < 0.05).<br>5. Growth-associated protein 43 (GAP-43) significantly decreased in model rats (P < 0.01) and significantly increased after GZD treatment (P < 0.01).<br>6. Ciliary neurotrophic factor (CNTF) unchanged across groups (P > 0.05).	Guizhi Decoction (especially RC:RPA = 1:1 and 2:1) significantly reversed sympathetic denervation by increasing NE, TH, and GAP-43 and reducing NGF, thus maintaining sympathetic–vagal balance and protecting cardiac autonomic nerves.
Li 2014[8]	Streptozotocin (STZ)-induced diabetic cardiac autonomic neuropathy	36 male Wistar rats (8 weeks old, 280–300 g), divided into normal, model, methylcobalamin, and Guizhi Decoction (GZD) groups (n = 9 each)	Guizhi Decoction 4.0 g crude drug/kg/day by gavage for 4 weeks; methylcobalamin 0.15 mg/kg/day as positive control	1. Heart rate variability (HRV) parameters SDNN, RMSSD, PNN50, LF, HF, and total power significantly decreased in model group (P < 0.05), and LF/HF significantly increased (P < 0.05).<br>2. Guizhi Decoction significantly increased SDNN, RMSSD, PNN50, LF, HF, and total power (P < 0.05) and decreased LF/HF (P < 0.05).<br>3. Tyrosine hydroxylase (TH) significantly increased and choline acetyltransferase (ChAT) significantly decreased in myocardium of model rats (P < 0.05); Guizhi Decoction significantly decreased TH and increased ChAT (P < 0.05).<br>4. Growth-associated protein 43 (GAP-43) significantly increased, nerve growth factor (NGF) significantly decreased, and ciliary neurotrophic factor (CNTF) significantly increased after Guizhi Decoction (P < 0.05).	Guizhi Decoction significantly enhanced vagal tone, reduced sympathetic overactivity, and improved neurotrophic balance (↑ChAT, ↑CNTF, ↓TH, ↓NGF), restoring cardiac autonomic regulation in diabetic rats.
Li 2015[9]	STZ-induced diabetic cardiac autonomic neuropathy (DCA	60 male Wistar rats (8 weeks old, 250–300 g), divided into control, model, methylc	Guizhi Decoction (RC:RPA ratios 1:1, 2:1, 1:2), 4.0–5.5 g crude drug/kg/day by ga	1. Tyrosine hydroxylase (TH), TH/ChAT ratio, and nerve growth factor (NGF) significantly increased in model group (P < 0.05, P < 0.01); ChAT and CNTF significantly decreased (P < 0.05, P < 0.01).	Guizhi Decoction (Guizhi:Shaoyao = 1:1) most effectively restored cardiac sympathetic–vag

	N)	obalamin, and Guizhi Decoction subgroups with Guizhi–Shaoyao ratios of 1:1, 2:1, and 1:2 (n = 10 each)	vage for 4 weeks; methylcobalamin 0.15 mg/kg/day as control	2. Guizhi Decoction (1:1) significantly decreased TH, TH/ChAT ratio, and NGF in left ventricle and septum ($P < 0.05$, $P < 0.01$).<br>3. Guizhi Decoction (1:1) significantly increased ChAT and CNTF in left ventricle and septum ($P < 0.05$, $P < 0.01$).<br>4. Guizhi Decoction (1:2) moderately increased ChAT and CNTF and decreased TH/ChAT ratio ($P < 0.05$, $P < 0.01$).<br>5. Guizhi Decoction (2:1) showed weaker effects, with higher TH and TH/ChAT ratio ($P < 0.05$, $P < 0.01$).	al balance by reducing TH and NGF and enhancing ChAT and CNTF, protecting vagal function in diabetic autonomic neuropathy.
Sun 2022[10]	Ovalbumin (OVA) + cigarette smoke (CS)–induced aggravated asthma	70 female C57BL/6 mice (8–10 weeks, 20 ± 2 g), divided into normal saline, model (OVA + CS), dexamethasone (0.1 mg/kg), high-dose HPMHD (12 g/kg), low-dose HPMHD (6 g/kg), n-butanol extract (0.5 g/kg), and ethyl acetate extract (0.4 g/kg) groups	Houpo-Mahuang Decoction (HPMHD) aqueous extract and active fractions by gavage daily from day 29 to 42 after OVA + CS sensitization and challenge	1. Airway hyperresponsiveness (Penh) significantly increased in OVA + CS group ($P < 0.01$) and significantly decreased after high-dose HPMHD and ethyl acetate extract treatment ($P < 0.01$).<br>2. Eosinophil and neutrophil counts in bronchoalveolar lavage fluid (BALF) significantly increased in OVA + CS group ($P < 0.01$) and significantly decreased after high-dose HPMHD, n-butanol, and ethyl acetate extract treatment ($P < 0.01$).<br>3. Interleukin (IL)-4, IL-5, and IL-13 levels in BALF significantly increased in model group ($P < 0.01$) and significantly decreased after HPMHD and extract treatments ($P < 0.01$).<br>4. Transient receptor potential ankyrin A1 (TRPA1) mRNA and protein expression significantly increased in OVA + CS group ($P < 0.01$) and significantly decreased after HPMHD and ethyl acetate extract treatments ($P < 0.01$).<br>5. Zonula occludens-1 (ZO-1) and occludin expression significantly decreased in OVA + CS group ($P < 0.01$) and significantly increased after HPMHD and extract treatments ($P < 0.01$).<br>6. Claudin-2 expression significantly increased in OVA + CS group ($P < 0.01$) and significantly decreased after HPMHD and extract treatments ($P < 0.01$).	HPMHD significantly reduced airway inflammation and hyperresponsiveness by downregulating TRPA1 and restoring tight junction proteins (ZO-1, occludin, claudin-2), thereby protecting epithelial barrier integrity and attenuating neurogenic airway hypersensitivity.
Sun 2025[11]	Acute intracerebral hemorrhage (AICH) induced by autologous blood injection into right caudate nucleus	Adult male Sprague–Dawley rats (220–250 g), divided into blank, sham, model, LTP-L (10 g/kg), LTP-H (20 g/kg), probiotics, and pseudo-germ-free (PGF) groups	Liangxue Tongyu Prescription (LTP) 10 or 20 g/kg by gavage daily for 3 days; probiotics 0.175 g/kg; antibiotics pretreatment for PGF group	1. Vagus nerve (VN) discharge frequency significantly decreased in model group ($P < 0.01$) and significantly increased after LTP-L treatment ($P < 0.01$).<br>2. Brain noradrenaline (NA), dopamine (DA), and glutamate (GLU) levels significantly decreased in model rats ($P < 0.01$) and significantly increased after LTP-L ($P < 0.01$).<br>3. Brain-derived neurotrophic factor (BDNF) and nerve growth factor (NGF) expression significantly decreased in model group ($P < 0.05$) and significantly increased after LTP-L ($P < 0.05$).<br>4. Brain gastrin (GAS) and motilin (MTL) levels significantly increased, while intestinal GAS, MTL, and ghrelin significantly decreased after AICH ($P < 0.05$); LTP-L normalized both brain and gut peptide levels ($P < 0.05$).<br>5. Intestinal motility and mucosal structure significantly improved after LTP-L compared to model ($P < 0.01$).<br>6. Removal of intestinal microbiota (PGF group) abolished LTP-induced increase of BDNF and NGF and reduction of neuronal apoptosis ($P > 0.05$).	LTP significantly enhanced vagal activity, increased neurotransmitters (NA, DA, GLU), normalized brain–gut peptide levels, and upregulated BDNF/NGF via modulation of the microbiota–gut–brain axis to protect neurons after intracerebral hemorrhage.
Zhang 2011[12]	D-galactose (D-gal)–induced brain aging model (cholinergic dysfunction)	64 Sprague–Dawley rats (male and female, 7 weeks old), divided into saline control, LWDH control, D-gal model, and D-gal + LWDH groups (n = 8 per sex per group)	D-gal 100 mg/kg/day subcutaneous for 6 weeks; Liu Wei Dihuang (LWDH) decoction 0.01 mL/g/day orally for 6 weeks starting from week 3	1. Escape latency in Morris water maze significantly increased in D-gal group ($P < 0.01$) and significantly decreased after LWDH ($P < 0.01$).<br>2. Swimming speed significantly decreased in D-gal group ($P < 0.01$) and significantly increased after LWDH ($P < 0.01$).<br>3. Acetylcholine (ACh) level in visual cortex significantly decreased in D-gal group ($P < 0.001$) and significantly increased after LWDH ($P < 0.001$).	LWDH significantly protected against D-gal-induced neurodegeneration by increasing acetylcholine synthesis (↑ACh, ↑ChAT) and reducing acetylcholinesterase activity, thereby res

				4. Choline acetyltransferase (ChAT) activity significantly decreased in D-gal group ($P < 0.001$) and significantly increased after LWDH ($P < 0.001$).<br>5. Acetylcholinesterase (AChE) activity significantly increased in D-gal group ($P < 0.001$) and significantly decreased after LWDH ($P < 0.001$).<br>6. ChAT- and AChE-positive cell numbers in visual cortex significantly decreased in D-gal group ($P < 0.001$) and significantly increased after LWDH ($P < 0.001$).<br>7. Neuronal density in hippocampus and visual cortex significantly decreased in D-gal group ($P < 0.001$) and significantly increased after LWDH ($P < 0.001$).	toring cholinergic transmission and cognitive performance.
Bai 2019[13]	Bilateral common carotid artery occlusion (BCCAo)–induced cerebral ischemia	32 male Sprague–Dawley rats (250 ± 10 g), divided into sham-operation, model-operation, nimodipine (8.1 mg/kg), and Pei Yuan Tong Nao (PYTN) groups (n = 8 each)	PYTN 0.486 g/kg/day by gavage for 8 weeks after BCCAo; nimodipine 8.1 mg/kg/day as positive control	1. Escape latency in Morris water maze significantly increased in model group ($P < 0.01$) and significantly decreased after PYTN and nimodipine ($P < 0.05$).<br>2. Standard uptake value (SUV) for F-FDG in PET-CT significantly decreased in model group ($P < 0.01$) and significantly increased after PYTN and nimodipine ($P < 0.05$).<br>3. Acetylcholine (ACh) level in hippocampus significantly decreased in model group ($P < 0.01$) and significantly increased after PYTN and nimodipine ($P < 0.05$).4. α7 nicotinic acetylcholine receptor (α7nAChR) protein expression in hippocampus significantly decreased in model group ($P < 0.01$) and significantly increased after PYTN ($P < 0.05$, $P < 0.01$).	PYTN significantly enhanced cognitive function and hippocampal metabolism by increasing acetylcholine content and α7 nicotinic acetylcholine receptor expression, indicating improved cholinergic transmission and neuroprotection in cerebral ischemia.
Nakyam 2023[14]	Metabolic syndrome (MetS) combined with ischemic stroke (MCAO/reperfusion injury)	48 male Wistar rats (180–220 g), divided into 8 groups (normal diet, MetS + sham, MetS + MCAO + vehicle, vitamin C 250 mg/kg, donepezil 3 mg/kg, GCJ 100 mg/kg, GCJ 200 mg/kg, GCJ 300 mg/kg)	Polyherbal formulation (GCJ) containing ginger + Chinese date + wood ear mushroom, given orally 21 days before and 21 days after MCAO	1. Escape latency in Morris water maze significantly increased in MetS + MCAO group ($P < 0.001$) and significantly decreased after GCJ 100–300 mg/kg ($P < 0.01$).<br>2. Retention time significantly decreased in MetS + MCAO group ($P < 0.001$) and significantly increased after GCJ 100–300 mg/kg ($P < 0.01$).<br>3. Novel object recognition (NOR) ratio significantly decreased in MetS + MCAO group ($P < 0.001$) and significantly increased after GCJ 100–300 mg/kg ($P < 0.01$).<br>4. Acetylcholinesterase (AChE) activity in prefrontal cortex and hippocampus significantly increased in MetS + MCAO group ($P < 0.001$) and significantly decreased after GCJ 100–300 mg/kg ($P < 0.001$).<br>5. Endothelial nitric oxide synthase (eNOS), brain-derived neurotrophic factor (BDNF), and phosphorylated extracellular signal-regulated kinase (pERK/ERK) expression significantly decreased in MetS + MCAO group ($P < 0.001$) and significantly increased after GCJ 100–300 mg/kg ($P < 0.01$, $P < 0.001$).<br>6. Neuronal density in prefrontal cortex and hippocampus (CA1–CA3, dentate gyrus) significantly decreased in MetS + MCAO group ($P < 0.001$) and significantly increased after GCJ 200–300 mg/kg ($P < 0.01$, $P < 0.001$).	GCJ significantly improved memory and neural plasticity by suppressing acetylcholinesterase and enhancing eNOS, BDNF, and pERK/ERK signaling, thereby increasing cholinergic activity and neuronal survival in ischemic–metabolic syndrome rats.
Jiang 2013[15]	Ovalbumin (OVA) sensitization + lipopolysaccharide (LPS)–enhanced severe asthma	120 female Balb/C mice (18–20 g, 6–8 weeks old), divided into 6 groups: normal, model, dexamethasone (0.0024 g/kg), and Qi'ao Decoction (QAD) low (6.7 g/kg), medium (13.4 g/kg), and high (26.8 g/kg)	Qi'ao Decoction by gavage once daily on days 28–34 after OVA + LPS challenge; dexamethasone 0.0024 g/kg/day for 7 days	1. Total cellular count, macrophages, lymphocytes, eosinophils, and neutrophils in bronchoalveolar lavage fluid (BALF) significantly increased in model group ($P < 0.01$) and significantly decreased after QAD (medium dose, $P < 0.01$) and dexamethasone ($P < 0.05$).<br>2. Airway resistance (RL) before and after acetylcholine (ACh, 5 mg/mL) stimulation significantly increased in model group ($P < 0.01$) and significantly decreased in all QAD dose groups ($P < 0.01$).<br>3. Lung histopathological score significantly increased in model group and significantly decreased after QAD-M and QAD-H treatment ($P < 0.01$).	QAD significantly inhibited airway inflammation and acetylcholine-induced hyperresponsiveness by restoring Th1/Th2 cytokine balance, indicating suppression of cholinergic-mediated bronchoconstriction and immune modulation in severe asthma.

				4. Interleukin-4 (IL-4) level significantly increased in model group (P < 0.05) and significantly decreased after QAD-M (P < 0.05).<br>5. Interferon-gamma (IFN-γ) and interleukin-12 (IL-12) levels significantly decr eased in model group (P < 0.05, P < 0.01) and significantly increased aft er QAD-M and QAD-H treatment (P < 0.05, P < 0.01).	
Gan 2023[16]	Post-infectious coug h (PIC) and chronic bronchitis induced by smoke exposure	Mice and guinea pigs (SPF-g rade); mice (18–22 g) for a mmonia-induced cough; guin ea pigs (300–400 g) for vag us nerve–stimulated cough a nd smoke-induced chronic b ronchitis	Shegan Zhike Capsule (SZC) extract at 26, 52, or 104 mg/kg; orally once daily fo r 7 days in vagus-stimulati on test and 2 weeks in s moke-induced model	1. Cough frequency in ammonia-induced mice significantly decreased at 43, 8 6, and 172 mg extract/kg (P < 0.05, P < 0.01).<br>2. Antitussive onset time at 43 mg/kg observed by day 5 and day 7 (P < 0. 05, P < 0.01).<br>3. Electrical stimulation of vagus nerve–induced cough in guinea pigs significa ntly suppressed at 26, 52, and 104 mg/kg (P < 0.05, P < 0.01).<br>4. Serum cyclooxygenase-2 (COX-2), prostaglandin E_2 (PGE_2), and thromboxan e A_2 (TXA_2) levels significantly increased in smoke-exposed controls (P < 0 .05, P < 0.01) and significantly decreased after SZC treatment at all doses (P < 0.01).	Shegan Zhike Capsule significa ntly inhibited vagus nerve–me diated cough and airway infla mmation by downregulating C OX-2 and reducing arachidoni c acid–derived mediators (PGE_2, TXA_2), indicating suppressio n of peripheral neurogenic an d inflammatory pathways in p ost-infectious cough.
Wu 2019[17]	Scopolamine-induce d amnesia (choliner gic dysfunction mo del of dementia)	50 male Sprague–Dawley rat s (10 weeks old, 300 ± 20 g), divided into control, mod el, and Shenmayizhi Decocti on (SMYZ) low (3.3 g/kg), m edium (6.6 g/kg), and high (16.5 g/kg) groups	Shenmayizhi Decoction (SM YZ) administered by gavage daily for 4 weeks; scopola mine 0.7 mg/kg intraperito neally for 5 days before b ehavioral testing	1. Escape latency in Morris water maze significantly increased in model grou p and significantly decreased after medium- and high-dose SMYZ (P<0.01).<br>2. Time and distance in target quadrant significantly decreased in model gro up (P < 0.05) and significantly increased after SMYZ-Medium and SMYZ-Hi gh (P < 0.05).<br>3. Acetylcholinesterase (AChE) expression and activity in hippocampus signific antly increased in model group (P < 0.01) and significantly decreased afte r SMYZ-Low, SMYZ-Medium, and SMYZ-High (P < 0.05, P < 0.01).<br>4. Choline acetyltransferase (ChAT) expression and activity significantly decrea sed in model group (P < 0.01) and significantly increased after SMYZ- Hig h (P < 0.05, P < 0.01).<br>5. Acetylcholine muscarinic M1 receptor expression significantly decreased in model group (P < 0.01) and significantly increased after SMYZ- High (P < 0.01); M2 receptor unchanged (P > 0.05).	Shenmayizhi Decoction signific antly ameliorated scopolamine -induced memory impairment by enhancing hippocampal ch olinergic transmission, indicati ng restoration of acetylcholin e metabolism and receptor si gnaling.
Wu 2015[18]	Hypertension induc ed by abdominal a ortic coarctation	50 male Wistar rats (170–22 0 g), divided into sham-oper ation, model, Sini Decoction (SND), SND + perindopril, an d perindopril groups (n = 1 0 each)	Sini Decoction 0.81 g/kg/da y by gavage for 8 weeks; perindopril 0.36 mg/kg/day; combined SND + perindop ril as combination group	1. Systolic blood pressure (SBP) significantly increased in model group after 4 weeks (P < 0.001) and significantly decreased after 4 weeks of SND, S ND + perindopril, and perindopril treatment (P < 0.001).<br>2. Diastolic blood pressure (DBP) and mean arterial pressure (MAP) slightly d ecreased after treatment compared with model (P < 0.05).<br>3. Baroreflex sensitivity (BRS) significantly decreased in model group (P < 0.0 01) and significantly increased after SND, SND + perindopril, and perindop ril treatment (P < 0.001).<br>4. SND + perindopril group exhibited higher BRS improvement compared wit h SND alone (P < 0.05).	Sini Decoction significantly lo wered systolic blood pressure and enhanced baroreflex sens itivity by improving vagal refl ex responsiveness, indicating modulation of autonomic bala nce and vascular reflex contr ol in hypertensive rats.
Qi 2014[19]	Clonidine (CLO)-ind uced bradyarrhyth mia	40 Sprague–Dawley rats (17 0–220 g, both sexes), divide d into control, model, low-d ose, and high-dose WenYang Tongzhi (WYT) groups, plus Xinbao positive control	WYT capsule 400 or 800 mg/kg by gavage; Xinbao c apsule 400 mg/kg as positi ve control; single daily dos e, 2 h observation post-ad ministration	1. Heart rate (HR) significantly decreased in model group (P < 0.05) and sig nificantly increased after WYT 400–800 mg/kg (P < 0.05, P < 0.01).<br>2. Heart rate variability (HRV) coefficient significantly decreased after WYT 4 00–800 mg/kg (P < 0.05, P < 0.01), with maximal effect at 1–1.5 h.<br>3. Atrioventricular block incidence significantly decreased after WYT 400–800 mg/kg compared with model group (P < 0.05, P < 0.01).<br>4. High-dose WYT exhibited stronger effects than Xinbao on HR increase and HRV reduction (P < 0.05).	WYT significantly alleviated cl onidine-induced bradyarrhyth mia by increasing heart rate, reducing heart rate variability, and lowering atrioventricular block incidence, indicating mo dulation of vagal excitability a nd improvement of cardiac a utonomic balance.

Ma 2016[20]	Chronic heart failure (CHF) induced by coronary artery ligation	70 male Sprague–Dawley rats (200–230 g), divided into sham, model, Yiqihuoxue (YQHX) compound, exercise, YQHX + exercise, lisinopril, and lisinopril + exercise groups (n = 10 each)	Yiqihuoxue compound decoction (YQHX) by gavage daily for 6 weeks; exercise training (swimming 50 min/day, 6 weeks); lisinopril 10 mg/kg/day as control	1. Plasma norepinephrine (NE) level significantly increased in model group (P < 0.01) and significantly decreased after YQHX, exercise, and combination treatments (P < 0.01, P < 0.05).<br>2. Plasma acetylcholine (ACh) level significantly decreased in model group (P < 0.01) and significantly increased after YQHX, exercise, and combination treatments (P < 0.01, P < 0.05).<br>3. Tyrosine hydroxylase (TH) expression in myocardium significantly increased in model group (P < 0.01) and significantly decreased after all treatments (P < 0.05, P < 0.01).<br>4. Choline acetyltransferase (ChAT) expression significantly decreased in model group (P < 0.01) and significantly increased after YQHX, exercise, and combination treatments (P < 0.05, P < 0.01).<br>5. The YQHX + exercise group exhibited the most pronounced improvement in NE, ACh, TH, and ChAT levels (P < 0.01).	Yiqihuoxue compound decoction combined with exercise significantly restored autonomic balance in heart failure rats by decreasing sympathetic marker TH and norepinephrine and increasing parasympathetic markers ChAT and acetylcholine, indicating improved vagal tone and reduced sympathetic overactivity.
Du 2006[21]	Anorexia induced by special low-protein forage (hypothalamic satiety imbalance)	45 Sprague–Dawley rats (35–40 days old, 60 ± 10 g), divided into control, model, and Yunpi-treated groups (n = 15 each)	Yunpi prescription (Erbao granule extract, 37.6g/kg/day, 2× clinical equivalent) by gavage for 3 weeks after 1 week of model induction	1. Daily food intake significantly decreased in model group (P < 0.05) and significantly increased after Yunpi treatment (P < 0.05).<br>2. Ventromedial hypothalamic nucleus (VMN) neuron excitatory response duration to gastric vagus stimulation significantly prolonged in model group (P < 0.05) and significantly shortened after Yunpi treatment (P < 0.05).<br>3. Required stimulation intensity for vagal activation significantly decreased in model group (P<0.05) and significantly increased after Yunpi treatment (P < 0.05).<br>4. Proportion of VMN neurons sensitive to cholecystokinin (CCK) significantly increased in model group (P<0.05) and significantly decreased after Yunpi treatment (P < 0.05).	Yunpi prescription significantly reduced VMN neuron sensitivity to gastric vagus and cholecystokinin signaling, normalizing central–vagal satiety feedback and enhancing appetite regulation in anorexic rats.
Tan 2017[22]	Spleen-deficiency model induced by bitter-cold purgation and irregular feeding	140 Sprague–Dawley rats (180 ± 20 g, male and female), divided into control, model, and 12 treatment groups (n = 10 each): Naringin (low/mid/high), Neohesperidin (low/mid/high), Naringin+Neohesperidin (low/mid/high), and Fructus Aurantii decoction (low/mid/high)	Fructus Aurantii decoction 0.104–0.416 g/mL (1×–4× clinical equivalent), or equivalent naringin and neohesperidin doses by gavage, 10 mL/kg/day for 7 days post-modeling	1. Plasma acetylcholine (ACh), serum gastrin (GAS), motilin (MTL), and substance P (SP) levels significantly decreased in model group (P < 0.01), while vasoactive intestinal peptide (VIP) significantly increased (P < 0.05).<br>2. Naringin mid/high-dose groups significantly increased ACh, GAS, MTL, and SP and significantly decreased VIP (P < 0.05, P < 0.01).<br>3. Neohesperidin mid/high-dose groups significantly increased ACh, GAS, MTL, and SP and significantly decreased VIP (P < 0.05, P < 0.01).<br>4. Naringin+Neohesperidin combinations significantly increased ACh, GAS, MTL, and SP and significantly decreased VIP in a dose-dependent manner (P < 0.05, P < 0.01).<br>5. Fructus Aurantii decoction high-dose group significantly increased GAS, MTL, and SP and significantly decreased VIP (P < 0.05, P < 0.01).	Fructus Aurantii and its flavonoid components (naringin, neohesperidin) significantly enhanced gastrointestinal motility by increasing acetylcholine and excitatory gut peptides (GAS, MTL, SP) and reducing inhibitory peptide VIP, indicating vagal–enteric modulation in spleen-deficiency rats.

Supplementary Table 4. Summary of studies on vagal modulation by single herb extracts

Study (Year)	Disease Model	Subjects	Intervention	Main Vagal Outcomes	Summary of findings
Niijima 1998[23]	Normal anesthetized rat model (taste-induced vagal reflex study)	30 male Wistar rats (≈300 g) divided into: Pinellia ternata, Zingiberis rhizoma, mixture (5: 1), lingual-nerve-sectioned + Pinellia, lingual-nerve-sectioned + Zingiberis, and glossopharyngeal-nerve-sectioned groups (n=5 each)	Taste stimulation on tongue with aqueous extract (50 mg/ml, 10 min) of (1) Pinellia ternata (tuber), (2) Zingiberis rhizoma (rhizome), or (3) mixture (5: 1); applied once under urethane anesthesia	1. Pinellia ternata significantly suppressed efferent activity of the gastric vagus nerve (−18 – 30 %, $P < 0.05$).<br>2. Zingiberis rhizoma significantly increased efferent vagal activity (+30 – 45 %, $P < 0.01$).<br>3. The mixture showed no significant change (n.s.).<br>4. Glossopharyngeal-nerve section abolished both excitatory and inhibitory responses, indicating glossopharyngeal afferent mediation.	Astringent taste of Pinellia ternata suppressed, while hot taste of Zingiberis rhizoma enhanced gastric vagal outflow; combined use neutralized suppression through glossopharyngeal-mediated reflex activation.
Lu 1984[24]	Stress-induced gastric ulcer (restraint + cold water immersion)	36 male Wistar rats (≈180 g) divided into six groups: normal control, raw Rheum palmatum, rice-wine-stewed rheum, stress control, stress + raw rheum, and stress + rice-wine-stewed rheum (n = 6 each)	Rheum palmatum powder (0.075 g/100 g body weight per dose, gavage twice daily for 3 days before stress); stewed preparation made with rice wine at same dose and schedule	1. Plasma cyclic adenosine monophosphate (cAMP) and cyclic guanosine monophosphate (cGMP) significantly decreased in stressed rats ($P < 0.01$).<br>2. cAMP/cGMP ratio significantly increased ($P < 0.001$).<br>3. Raw rheum had no effect on these changes.<br>4. Rice-wine-stewed rheum significantly reduced the elevated cAMP/cGMP ratio ($P < 0.01$), approaching normal levels.	Rice-wine-processed rheum restored autonomic balance in stress-ulcer rats by normalizing the plasma cAMP/cGMP ratio and re-establishing sympathetic–parasympathetic equilibrium.
Luo 1996[25]	Normal anesthetized rat model (vagal electrophysiological study)	28 Wistar rats (250–350 g, both sexes), randomly divided into two main sets (intravenous and intracerebroventricular experiments; n = 14 each), further divided into extract and control groups (n = 7 each)	Alfalfa (Medicago sativa) aqueous extract (0.05%, 1 mL via lingual vein or lateral ventricle; single administration under urethane anesthesia); controls received saline or artificial cerebrospinal fluid	1. Lingual vein injection significantly increased cervical vagus nerve discharge frequency from 298.6 ± 4.5 to 369.6 ± 6.1 impulses/min ($P < 0.001$). Increased discharge persisted for 40 ± 3.5 min.<br>2. Lateral ventricle injection also significantly increased discharge (10 ± 2 impulses/s → 48.1 ± 4.1 impulses/s; $P < 0.001$). Response lasted 43 ± 3.5 min and had a 3–5 min latency.	Alfalfa extract significantly increased efferent vagus nerve discharge via both peripheral and central routes, indicating excitation of nuclei regulating vagal output.
Fu 2013[26]	Stress-induced gastric ulcer (restraint + water immersion)	10 adult female Sprague–Dawley rats (220–250 g), divided into normal saline control and Aloe-treated groups (n = 5 each)	Aloe extract (1 mL containing 0.165 g crude drug, 12 mL/kg, gavage twice daily for 7 days); control received saline	1. Gastric juice secretion significantly increased ($P < 0.05$).<br>2. Gastric juice pH significantly increased ($P < 0.01$).<br>3. Gastric mucosal structure preserved with intact glands and reduced necrosis.<br>4. Acetylcholinesterase (AChE)-positive cholinergic nerve fibers in gastric wall significantly decreased ($P < 0.05$).	Aloe treatment protected against stress ulcer by reducing vagal cholinergic activity and acetylcholine (ACh)–mediated gastric acid secretion.
Zhao 2010[27]	Scopolamine-induced memory impairment (central cholinergic dysfunction model)	90 Kunming mice (18–22 g, both sexes), randomly divided into nine groups (n = 10 each): normal control, model, nimodipine (30 mg/kg), and six Alpinia officinarum extract groups (water and ethanol extracts at 6.66, 3.33, and 1.67 mg/kg)	Alpinia officinarum water or ethanol extract (gavage once daily for 10 days); 10 mg/kg scopolamine intraperitoneal injection 50 min after last dose	1. Water and ethanol extracts significantly prolonged step-down latency and reduced error frequency ($P < 0.05$, $P < 0.01$).<br>2. Escape latency in Morris water maze significantly shortened ($P < 0.05$).<br>3. Acetylcholine transferase (ChAT) activity significantly increased in brain tissue ($P < 0.05$, $P < 0.01$).<br>4. True cholinesterase (TChE) activity significantly decreased ($P < 0.05$, $P < 0.01$).<br>5. Hippocampal CA3 neurons preserved with reduced degeneration and necrosis.	Alpinia officinarum extracts enhanced central cholinergic function by increasing ChAT and decreasing TChE activity, improving memory via protection of hippocampal neurons.
Li 2023[28]	Gastric cold syndrome model (ice-water and NaOH gavage; hypothermic and hypomotility model)	90 Kunming mice (18–22 g, both sexes) randomly divided into 9 groups (n = 10 each): normal, model, motilium, Fuzi Lizhong pill, dried gi	Alpinia officinarum (galangal) and Zingiber officinale (dried ginger) decoction extracts; combination doses: 2.7 + 5, 5.4 + 5, and 10.8 + 5 g/kg	1. Gastrin (GAS), motilin (MTL), acetylcholine (ACh), and superoxide dismutase (SOD) levels in gastric tissue significantly increased ($P < 0.05$, $P < 0.01$).<br>2. Malondialdehyde (MDA) and interleukin-6 (IL-6) levels significantly decreased ($P < 0.05$, $P < 0.01$).<br>3. Gastric evacuation rate, intestinal propulsion, body temperature, and body	Combined galangal + dried ginger extract alleviated gastric cold by enhancing cholinergic and motility-related mediators (ACh, GAS, MTL)

		nger, galangal, and low-, medium-, and high-dose galangal + dried ginger combination groups	respectively; gavage once daily for 30 days	mass significantly increased ($P < 0.05$, $P < 0.01$).<br>4. Gastric mucosa exhibited restored epithelial integrity and reduced inflammation.	and reducing oxidative and inflammatory stress.
El-Akabawy 2014[29]	Streptozotocin (STZ)-induced diabetes (type I diabetic encephalopathy model)	96 male albino rats (8–10 weeks old, 150–200 g), divided into 4 groups: control, control + ginger, diabetic, and diabetic + ginger; each group further subdivided by duration (4, 6, 8 weeks; n = 8 each)	Zingiber officinale (ginger) extract 500 mg/kg/day, oral gavage for up to 8 weeks following STZ injection (60 mg/kg, i.p.)	1. Inducible nitric oxide synthase (iNOS) expression significantly decreased in frontal cortex, hippocampus, and cerebellum ($P < 0.001$).<br>2. Caspase-3–positive apoptotic neurons significantly decreased ($P < 0.001$).<br>3. Tumor necrosis factor-α (TNF-α) expression significantly decreased ($P < 0.001$).<br>4. Glial fibrillary acidic protein (GFAP)-positive astrocytes significantly decreased ($P < 0.001$).<br>5. Acetylcholinesterase (AChE) activity significantly decreased in all brain regions ($P < 0.001$).<br>6. Ki67-positive neurogenic cells in dentate gyrus significantly increased ($P < 0.001$).	Ginger treatment significantly reduced oxidative stress, inflammation, apoptosis, and AChE activity while enhancing neurogenesis, indicating restoration of cholinergic and neuronal function in diabetic brain.
Zhong 2022[30]	Motion sickness model (rotational stimulation–induced autonomic dysfunction)	48 male Kunming mice (22–25 g), divided into six groups (n = 8 each): control, motion sickness, diphenhydramine (20 mg/kg), and ginger extract (100, 300, 500 mg/kg)	Zingiber officinale (ginger) ethanol extract (100–500 mg/kg, gavage 30 min before rotation); single administration; reference: diphenhydramine 20 mg/kg	1. Plasma acetylcholine (ACh) level significantly increased in motion sickness group (+123.4%, $P < 0.01$).<br>2. Ginger extract (GE) significantly decreased plasma ACh level by 41.1% ($P < 0.01$).<br>3. Plasma histamine level significantly increased in motion sickness group (+88%, $P < 0.001$).<br>4. GE significantly reduced histamine level by 41.8% ($P < 0.01$).<br>5. Superoxide dismutase (SOD) activity significantly increased ($P<0.05$), while blood urea nitrogen (BUN) and lactic acid (LA) remained unchanged.<br>6. Balance and coordination performance (beam test) significantly improved ($P < 0.05$).	Ginger extract significantly decreased acetylcholine (ACh) and histamine release in vestibular system, restoring autonomic balance through cholinergic and anti-inflammatory modulation.
Kim 2023[31]	Cisplatin-induced acute anorexia (chemotherapy-associated vagal dysfunction)	60 male Sprague–Dawley rats (6 weeks, 180–200 g), divided into groups: control, cisplatin (6 mg/kg, i.p.), cisplatin + ginger extract (GE 100 mg/kg), cisplatin + GE 500 mg/kg, [6]-gingerol + [6]-shogaol, and 5-HT$_3$A/5-HT$_4$ receptor antagonist controls (n = 8–10 each)	Zingiber officinale (ginger) ethanol extract (100 or 500 mg/kg, oral, single dose, co-administered with cisplatin 6 mg/kg i.p.); antagonists: palonosetron 0.1 mg/kg i.p. (5-HT$_3$A R), piboserod 1 mg/kg i.p. (5-HT$_4$ R)	1. Cisplatin significantly increased serotonin (5-HT) level in serum and nodose ganglion ($P < 0.01$).<br>2. Ginger extract (GE) 100 and 500 mg/kg significantly decreased 5-HT in serum and nodose ganglion ($P < 0.01$).<br>3. Gene expression of 5-HT$_3$A and 5-HT$_4$ receptors in nodose ganglion significantly upregulated by cisplatin was downregulated by GE ($P<0.0001$).<br>4. Protein expression of 5-HT$_3$A and 5-HT$_4$ receptors significantly decreased after GE 100 mg/kg ($P < 0.001$).<br>5. Food intake and body weight significantly increased at 48 h after GE 100 mg/kg ($P < 0.01$).<br>6. Combined [6]-gingerol + [6]-shogaol significantly reversed anorexia, while each alone was ineffective.	Ginger extract alleviated cisplatin-induced anorexia by downregulating vagal nodose-ganglion 5-HT$_3$A and 5-HT$_4$ receptor activity, reducing serotonin signaling, and restoring feeding behavior.
Zhao 2024[32]	Lipopolysaccharide (LPS)-induced acute lung injury (ALI)	120 male BALB/c mice (8–10 weeks old, 22 ± 2 g), divided into 6 groups (n = 20 each): control, LPS, dexamethasone (4 mg/kg), and LRC extract (200, 400, 800 mg/kg); separate groups for α7nAChR inhibition assay (n = 8 each)	Monochasma savatieri (Lu Rong Cao, LRC) aqueous extract (200–800 mg/kg, oral gavage, 7 days before LPS 5 mg/kg i.p. challenge); α7 nicotinic acetylcholine receptor (α7nAChR) inhibitor MLA (5 mg/kg, i.v.) used to block CAP signaling	1. Lung injury score, wet/dry weight ratio, and myeloperoxidase (MPO) activity significantly decreased ($P < 0.01$).<br>2. Inducible nitric oxide synthase (iNOS) and cyclooxygenase-2 (COX2) activity significantly decreased ($P < 0.05$).<br>3. Apoptotic cell number (TUNEL-positive) significantly decreased, while Bcl-2 expression significantly increased ($P < 0.01$).<br>4. Acetylcholine (ACh) and glutamine levels in lung tissue significantly increased ($P < 0.01$).<br>5. α7nAChR expression significantly increased, while Toll-like receptor 4 (TLR4) and phosphorylated nuclear factor-κB p65 (p-NF-κB p65) expression significa	Monochasma savatieri extract alleviated acute lung injury by activating α7nAChR-mediated cholinergic anti-inflammatory pathway (CAP) and suppressing the TLR4/NF-κB p65 inflammatory cascade.

				ntly decreased (P < 0.01).<br>6. Levels of tumor necrosis factor-α (TNF-α), interleukin-1β (IL-1β), and interle ukin-6 (IL-6) in bronchoalveolar lavage fluid and serum significantly decreas ed (P < 0.01).<br>7. The α7nAChR inhibitor MLA abolished LRC-induced suppression of p-NF-κB p65 and increased apoptosis (P < 0.05).	
Shih 2008[33]	Normal anesthetized rat model (autonomic cardiovascular regula tion study)	103 male Sprague–Dawley r ats (250–300 g) divided into groups for dose–response a nd antagonist studies (n = 5 –6 per group)	Rhodiola sacra radix extracts : water-soluble fraction (WtF , 10–75 mg/kg, i.v.) and n-b utanol-soluble fraction (BtF, 10–75 mg/kg, i.v.); pretreat ments: hexamethonium 30 mg/kg, reserpine 5 mg/kg, c aptopril 2.5 mg/kg, methylat ropine 1 mg/kg, NG-nitro-L-a rginine methyl ester (L-NAM E) 20 mg/kg	1. WtF significantly decreased systolic, diastolic, and mean systemic arterial pr essure in a dose-dependent manner (P < 0.05).<br>2. Heart rate and maximal rate of left ventricular pressure development (dP/ dtmax) significantly increased (P < 0.05).<br>3. Power density of vasomotor sympathetic components significantly decrease d, indicating reduced sympathetic tone (P < 0.05).<br>4. Hypotensive, chronotropic, and inotropic effects were abolished by hexamet honium or reserpine.<br>5. Captopril significantly potentiated hypotension (P < 0.05).<br>6. Methylatropine significantly reversed positive inotropic and chronotropic eff ects (P < 0.05).<br>7. L-NAME had no significant effect (n.s.).	Water-soluble extract of Rh odiola sacra induced hypot ension by suppressing symp athetic vasomotor tone and renin–angiotensin activity, while increasing heart rate and contractility through va gal cholinergic inhibition.
Cui 2012[34]	Lipopolysaccharide (LP S)-induced acute lung injury (ALI)	108 male Sprague–Dawley r ats (220 ± 20 g) randomly divided into six groups (n = 18 each): control, model, Sc utellaria baicalensis extract (SBWE) control, SBWE high-d ose + LPS, SBWE low-dose + LPS, and galantamine posi tive control	Scutellaria baicalensis water extract (SBWE) 3.0 g/kg (hig h) or 1.5 g/kg (low), gavage once daily for 7 days befor e LPS 5 mg/kg i.v.; galanta mine 5 mg/kg i.p. on day o f experiment	1. Lung wet/dry weight ratio significantly decreased at 2 h after LPS (P<0.05, P < 0.01).<br>2. Rectal temperature drop after LPS significantly reduced in SBWE high-dose group (P < 0.05).<br>3. Serum tumor necrosis factor-α (TNF-α) and nitric oxide (NO) levels significa ntly decreased at 2–4 h after LPS (P < 0.05, P < 0.01).<br>4. Serum acetylcholine (ACh) level significantly increased at 2h after LPS (P < 0.05, P < 0.01).<br>5. Choline acetyltransferase (ChAT) activity in lung tissue increased but not si gnificantly; acetylcholinesterase (AChE) activity slightly increased at 1 & 4h (P < 0.05).<br>6. Galantamine group similarly increased ACh and reduced AChE activity (P < 0.01).	Scutellaria baicalensis extrac t alleviated LPS-induced lun g injury by activating the c holinergic anti-inflammatory pathway, increasing acetylc holine (ACh) release, and r educing TNF-α and NO leve ls.
Medeiros 20 06[35]	Normal normotensive rat model (autonomic cardiovascular regula tion)	72 male Wistar rats (200–30 0 g) divided into groups: co ntrol, atropine (2 mg/kg), h examethonium (20 mg/kg), L -NAME (20 mg/kg), and bila teral vagotomy (n = 6 per g roup)	Sida cordifolia aqueous fracti on of hydroalcoholic leaf ext ract (AFSC; 5–40 mg/kg, i.v. bolus); additional groups pre treated with atropine, hexa methonium, or L-NAME	1. AFSC (5–40 mg/kg) significantly decreased mean arterial pressure by 6–33% and heart rate by 0–80% (P < 0.05–0.001).<br>2. Atropine pretreatment completely abolished hypotension and bradycardia (P < 0.001).<br>3. Hexamethonium pretreatment significantly potentiated hypotension (+12–32 %, P < 0.05) and attenuated bradycardia (−90%, P < 0.05).<br>4. Combined hexamethonium + L-NAME pretreatment significantly attenuated hypotension (P < 0.05), indicating nitric oxide (NO) involvement.<br>5. Bilateral vagotomy markedly reduced bradycardia (−70–90%, P < 0.01) but only slightly attenuated hypotension.<br>6. AFSC 40 mg/kg induced sinoatrial blockade, abolished by atropine pretreat ment.	Sida cordifolia extract induc ed hypotension through en dothelial muscarinic recepto r–mediated nitric oxide rele ase and vagus-dependent b radycardia via cardiac musc arinic (M_2) activation.
Wang 2021[36]	Scopolamine-induced l earning and memory impairment (central c holinergic dysfunction model)	90 male Kunming mice (25 ± 2 g) randomly divided int o six groups (n = 15 each): control, scopolamine model, donepezil (0.65 mg/kg), anti-	Drynaria fortunei water extr act (0.975 g/kg/day, gavage for 14 days); ER antagonist ICI182780 (0.072 mg/kg, i.p.) given before D. fortunei; sc	1. Acetylcholine (ACh) level in hippocampus significantly increased (P < 0.01).<br>2. Choline acetyltransferase (ChAT) activity significantly increased (P < 0.01).<br>3. Acetylcholinesterase (AChE) activity significantly decreased (P < 0.01).<br>4. Expression of estrogen receptor β (ERβ), ERα, follicle-stimulating hormone r eceptor (FSHR), and luteinizing hormone receptor (LHR) in hippocampus sig	Drynaria fortunei improved cognitive function by activa ting estrogen receptor β (E Rβ)–mediated cholinergic si gnaling, enhancing acetylch

		brain-failure capsule (585 mg/kg), Drynaria fortunei (0.975 g/kg), and D. fortunei + ER antagonist ICI182780 (0.072 mg/kg)	opolamine 3 mg/kg i.p. daily to induce model	nificantly increased ($P < 0.01$).<br>5. ER antagonist ICI182780 reversed all effects ($P < 0.05$–0.01).<br>6. Learning and memory performance in Morris water maze and novel object recognition tests significantly improved ($P < 0.01$).	oline synthesis (↑ChAT, ↓AChE) and hippocampal neurotransmission.
Asuku 2024[37]	Mercury chloride ($HgCl_2$)–induced neurotoxicity and cognitive impairment	35 male Wistar rats (150–200 g) randomly divided into five groups (n = 7 each): control, $HgCl_2$ (5 mg/kg), N-acetylcysteine + $HgCl_2$, 6-GIRIFZO (100 mg/kg) + $HgCl_2$, and 6-GIRIFZO (200 mg/kg) + $HgCl_2$	6-gingerol-rich fraction of Zingiber officinale (6-GIRIFZO; 100 or 200 mg/kg, oral, 14 days) co-administered with $HgCl_2$ (5 mg/kg, oral); reference: N-acetylcysteine (50 mg/kg)	1. Acetylcholinesterase (AChE) activity in brain significantly increased after $HgCl_2$ exposure ($P < 0.05$).<br>2. 6-GIRIFZO 100–200 mg/kg significantly decreased AChE activity toward control level ($P < 0.05$).<br>3. Brain nitric oxide (NO) and malondialdehyde (MDA) levels significantly increased after $HgCl_2$ but significantly decreased with 6-GIRIFZO ($P < 0.05$).<br>4. Glutathione (GSH), superoxide dismutase (SOD), catalase (CAT), and glutathione peroxidase (GPx) levels significantly increased in 6-GIRIFZO groups ($P < 0.05$).<br>5. Tumor necrosis factor-α (TNF-α), interleukin-1β (IL-1β), interleukin-6 (IL-6), and nuclear factor-κB (NF-κB) levels significantly decreased in 6-GIRIFZO groups ($P < 0.05$).<br>6. Escape latency in Morris water maze significantly decreased ($P < 0.05$) and recognition ratio in novel object test significantly increased ($P < 0.05$) in 6-GIRIFZO groups.<br>7. Hippocampal neurons preserved with intact CA3 structure and reduced inflammation (H&E, ×400).	6-gingerol-rich Zingiber officinale fraction ameliorated mercury-induced neurotoxicity by suppressing acetylcholinesterase and inflammatory cytokines while enhancing antioxidant defenses and hippocampal cholinergic function.

Supplementary Table 5. Summary of studies on vagal modulation by phytoconstituents

Study (Year)	Disease Model	Subjects	Intervention	Main Vagal Outcomes	Summary of findings
Wang 2022[38]	Acute myocardial infarction (AMI) model by left anterior descending (LAD) coronary artery ligation	Male Sprague-Dawley rats (230–250 g, n = 130; 10 per group)	L-Borneolum (0.2, 0.1, 0.05 g·kg⁻¹), Natural Borneolum (0.6, 0.3, 0.15 g·kg⁻¹), Synthetic Borneolum (0.2, 0.1, 0.05 g·kg⁻¹), gavage 10 mL·kg⁻¹, 3 days pretreatment + 3 days post-surgery	1. Heart rate significantly decreased (*P < 0.05–0.01*).<br>2. ST segment and Q wave amplitudes significantly reduced (*P < 0.05–0.01*).<br>3. Heart-rate variability parameters SDNN and SD1 significantly increased (*P < 0.05*).<br>4. Low-frequency/high-frequency (LF/HF) ratio significantly decreased (*P < 0.05–0.01*), indicating improved autonomic balance.<br>5. Interleukin-1β (IL-1β) and Interleukin-6 (IL-6) mRNA levels significantly down-regulated (*P < 0.01*).<br>6. Hypoxia-inducible factor-1α (HIF-1α), vascular endothelial growth factor receptor-1 (VEGFR1), and CD34 protein expression significantly increased (*P < 0.05–0.01*).	Borneolum isomers improved HRV and electrophysiology via enhanced parasympathetic tone and anti-inflammatory HIF-1α/VEGF-mediated angiogenic pathway; L-Borneolum most effective, Synthetic least.
Zaki 2020[39]	Deltamethrin-induced hippocampal neurotoxicity	Adult male Wistar rats (n = 32)	Deltamethrin 15 mg·kg⁻¹ + Curcumin or Nano-curcumin (2.5 mg·kg⁻¹, oral gavage, 30 days)	1. Acetylcholinesterase (AChE) activity significantly decreased by 50 % with deltamethrin (P < 0.01); restored by curcumin (+50 %) and nano-curcumin (+75 %).<br>2. Malondialdehyde (MDA) increased by 354 % (P < 0.001), reduced 38–60 % by curcumin and nano-curcumin.<br>3. Glutathione (GSH), superoxide dismutase (SOD), and catalase (CAT) decreased 61 %, 61 %, 57 %, respectively; increased 69–125 %, 61–135 %, 80–118 % by curcumin/nano-curcumin.<br>4. Nuclear factor erythroid 2-related factor 2 (Nrf2) expression increased 200 % (P < 0.01); reduced 24–55 % after treatment.<br>5. Caspase-3 and glial fibrillary acidic protein (GFAP) expression increased 176 % (P < 0.01); normalized by nano-curcumin.<br>6. Cognitive function and hippocampal pyramidal cell density significantly improved (P < 0.001).	Nano-curcumin ameliorated oxidative and cholinergic damage more effectively than curcumin, restoring AChE activity and autonomic balance via antioxidant and anti-apoptotic mechanisms mediated by Nrf2/GCS-HS/LS pathway.
Ren 2012[40]	Alzheimer's disease (APP/PS1 double transgenic mice)	APP/PS1 transgenic mice (3 months old, n = 60) + C57BL/6J controls (n = 12)	Curcumin 400, 200, 100 mg·kg⁻¹; Rosiglitazone 10 mg·kg⁻¹; oral gavage 1 ×/day for 3 months	1. Escape latency in platform test significantly shortened in model group (P < 0.01); curcumin (all doses) significantly prolonged latency (P < 0.05–0.01).<br>2. Choline acetyltransferase (ChAT)-positive neurons in hippocampal CA1 significantly decreased (P < 0.01), restored in curcumin groups (P < 0.05–0.01).<br>3. Western blot: ChAT protein expression decreased by 53 % in model (P < 0.01), increased by 35–60 % after curcumin (P < 0.05–0.01).<br>4. Average gray value of ChAT immunostaining increased by 63 % in model (P < 0.01) and reduced after treatment (P < 0.05–0.01).<br>5. Behavioral improvement correlated with ChAT restoration.	Curcumin enhanced hippocampal cholinergic activity by increasing ChAT expression and acetylcholine synthesis, improving learning and memory in APP/PS1 mice via restoration of central cholinergic transmission.
Zhang 2012[41]	Alzheimer's disease ($A\beta_{25-35}$ + D-galactose-induced model)	Adult male Wistar rats (n = 90; 200–250 g)	Curcumin 40, 60, 100 mg·kg⁻¹ (low, medium, high doses); intraperitoneal injection daily for 4 wee	1. Escape latency in Morris water maze significantly increased in AD model (*P < 0.01*); significantly decreased after curcumin treatment (*P < 0.01*).<br>2. Platform quadrant swim distance percentage significantly decrease	Curcumin improved spatial learning and memory in AD rats by enhancing hippocampal and basa

			ks after modeling	d in AD group (*P* < *0.01*); significantly increased by curcumin, most at high dose (*P* < *0.05–0.01*).<br>3. Choline acetyltransferase (ChAT)-positive neurons in hippocampus and nucleus basalis of Meynert significantly decreased in AD group (*P* < *0.01*); significantly increased by curcumin in a dose-dependent manner (*P* < *0.05–0.01*).<br>4. Nitric oxide (NO) and malondialdehyde (MDA) levels significantly increased in AD model (*P* < *0.01*); significantly decreased by curcumin (*P* < *0.05–0.01*).<br>5. Superoxide dismutase (SOD) activity significantly decreased in AD group (*P* < *0.05*); significantly increased after curcumin (*P* < *0.05–0.01*).	l forebrain ChAT expression and reducing oxidative stress (NO, MDA) through antioxidant and cholinergic neuroprotective mechanisms.
Wang 2009[42]	Alzheimer's disease ($A\beta_{25-35}$-induced hippocampal injury model)	Sprague-Dawley rats (n = 50; 220–280 g; male and female, 8–12 weeks)	Curcumin 100 $mg\cdot kg^{-1}$ (oral, 1×/day), Indomethacin 0.3 $mg\cdot mL^{-1}$ (oral, 2×/day), and combined treatment; 14 days post-injection	1. Escape latency in Morris water maze significantly increased in Aβ model group (*P* < *0.05*); significantly decreased after curcumin (*P* < *0.05*) and further after curcumin + indomethacin (*P* < *0.05*).<br>2. Hippocampal choline acetyltransferase (ChAT) activity significantly decreased after Aβ injection (*P* < *0.05*); significantly increased by curcumin (*P* < *0.05*) and more by combination therapy (*P* < *0.05*).<br>3. Acetylcholinesterase (AChE) activity significantly increased in Aβ group (*P* < *0.05*); significantly reduced by curcumin and further reduced by curcumin + indomethacin (*P* < *0.05*).<br>4. Combined treatment normalized both ChAT and AChE activities to near-control levels (*P* < *0.05*).	Curcumin combined with indomethacin enhanced hippocampal cholinergic function by increasing ChAT activity and inhibiting AChE-mediated acetylcholine degradation, thereby improving spatial learning and memory in Alzheimer's model rats.
Abu-Taweel 2016[43]	Cadmium-induced neurotoxicity and behavioral dysfunction	Swiss-Webster mice (male & female, n = 120; 10 per group)	Curcumin 150 $mg\cdot kg^{-1}$ or 300 $mg\cdot kg^{-1}$ + cadmium 100 $mg\cdot kg^{-1}$; oral 1× daily for 2 weeks (curcumin given 1 h before Cd)	1. Acetylcholinesterase (AChE) activity in brain significantly decreased in Cd group (*P* < *0.001*); significantly increased after curcumin treatment dose-dependently (*P* < *0.01–0.001*).<br>2. Social investigation, attack time, and naso-nasal contacts significantly decreased in males (*P* < *0.001*); significantly increased with curcumin (*P* < *0.05–0.01*).<br>3. Latency to threat and latency to first bite significantly increased in Cd group (*P* < *0.05–0.001*); significantly decreased with curcumin (*P* < *0.05–0.01*).<br>4. Number of fights and bites significantly decreased (*P* < *0.01–0.001*); restored by curcumin dose-dependently.<br>5. Testosterone (males) and progesterone (females) significantly decreased by Cd (*P* < *0.01–0.001*); significantly increased after curcumin (*P* < *0.05–0.001*).<br>6. Red blood cells, hemoglobin, and platelets significantly decreased in Cd group (*P* < *0.05–0.01*); significantly increased after curcumin (*P* < *0.05–0.001*).	Curcumin ameliorated cadmium-induced neurobehavioral, hormonal, and cholinergic deficits by restoring brain AChE activity and autonomic balance through antioxidant and anti-inflammatory mechanisms.
Xu 2018[44]	Cerebral ischemia/reperfusion injury (middle cerebral artery occlu	Adult male Sprague-Dawley rats (n = 220; 250–300 g)	Vagus nerve stimulation (VNS: 0.5 mA, 20 Hz, 30 ms trains for 60 min) ± Curcumin 50 $mg\cdot kg^{-1}$ i.	1. Escape latency in Morris water maze significantly increased in CI/RI group (*P* < *0.01*); significantly decreased by VNS (*P* < *0.05*) and further by VNS + curcumin (*P* < *0.01*).<br>2. Correct rate in shuttle box test significantly decreased in CI/RI gr	Combined curcumin and vagus nerve stimulation synergistically restored cognitive and motor func

	sion, MCAO)		p. daily × 5 days before MCAO	oup ($P < 0.001$); significantly increased by combination treatment ($P < 0.05–0.01$).<br>3. Motor retention time in rota-rod test significantly reduced in CI/RI group ($P < 0.01$); significantly increased by VNS + curcumin ($P < 0.05–0.01$).<br>4. Neurological deficit score and infarct volume significantly decreased after combined treatment ($P < 0.05$).<br>5. Neural apoptosis (TUNEL-positive cells) significantly decreased ($P < 0.05$); Bcl-2 significantly increased and Bax significantly decreased ($P < 0.05$).<br>6. Interleukin-6 (IL-6), interleukin-1β (IL-1β), and tumor necrosis factor-α (TNF-α) levels significantly decreased ($P < 0.05–0.01$).<br>7. Phosphorylated AKT and ERK2 levels significantly increased in brain tissue ($P < 0.05$).	tion by reducing apoptosis and inflammatory cytokines and activating the AKT/ERK2 neuroprotective pathway.
Mulla 2025[45]	Chromium (Cr) and ammonia (NH_3)-induced neurotoxicity and oxidative stress	*Pangasianodon hypophthalmus* (n = 120; 10 per group; 40-day exposure)	Curcumin 0.1% or 0.2% dietary supplementation; concurrent exposure to Cr (2.5 $mg \cdot L^{-1}$) and NH_3 (2.0 $mg \cdot L^{-1}$); twice daily feeding for 40 days	1. Cortisol level significantly increased in Cr+NH_3 group ($P < 0.01$); significantly decreased by 0.1% and 0.2% curcumin ($P < 0.01$).<br>2. Heat shock protein 70 (HSP70) and inducible nitric oxide synthase (iNOS) expression significantly increased in Cr+NH_3 group ($P < 0.01$); significantly downregulated by curcumin ($P < 0.01$).<br>3. Acetylcholinesterase (AChE) activity in brain significantly decreased under Cr+NH_3 exposure ($P < 0.01$); significantly restored by curcumin, highest at 0.1% ($P < 0.01$).<br>4. Catalase (CAT), superoxide dismutase (SOD), and glutathione-S-transferase (GST) activities significantly increased in liver and kidney under Cr+NH_3 stress ($P < 0.01$); significantly normalized by 0.1% curcumin ($P < 0.05–0.01$).<br>5. Cytochrome P450 (CYP450) and caspase-3a (Cas3a) expression significantly upregulated in Cr+NH_3 group ($P < 0.01$); significantly downregulated by curcumin ($P < 0.01$).<br>6. Tumor necrosis factor-α (TNF-α) expression significantly decreased by curcumin ($P < 0.01$); total immunoglobulin (Ig) expression significantly increased ($P < 0.01$).<br>7. Growth hormone (GH) and growth hormone receptor 1 (GHR1) expression significantly increased ($P < 0.01$); myostatin (MYST) significantly decreased ($P < 0.01$).<br>8. Chromium bioaccumulation in brain, liver, and kidney significantly reduced by curcumin ($P < 0.01$).	Dietary curcumin (0.1%) mitigated chromium and ammonia neurotoxicity by restoring acetylcholinesterase (AChE), reducing oxidative and inflammatory markers (HSP70, iNOS, TNF-α), and enhancing growth and immune gene expression through antioxidant and cholinergic regulation.
Dou 2018[46]	Collagen-induced arthritis (CIA)	Female Wistar rats (6–8 weeks, 150 ± 20 g, n = 6 per group)	Curcumin 100 $mg \cdot kg^{-1}$, oral daily × 14 days; comparison with leflunomide 2 $mg \cdot kg^{-1}$ and nicotine 300 $\mu g \cdot kg^{-1}$; with/without vagotomy (VGX) or nicotinic receptor antagonis	1. Heart rate variability (HRV) SDNN and RMSSD significantly increased ($P<0.01$); low-frequency/high-frequency (LF/HF) ratio significantly decreased ($P<0.01$), indicating enhanced parasympathetic tone.<br>2. Choline acetyltransferase (ChAT) activity in synovium and serum significantly increased ($P < 0.01$).<br>3. Vesicular acetylcholine transporter (VAChT) expression in gut, brain, and synovium significantly increased ($P < 0.01$).<br>4. Acetylcholinesterase (AChE) activity unchanged ($P > 0.05$).<br>5. Serum TNF-α, IL-1β, IL-6, and IL-17A significantly decreased ($P <$	Curcumin attenuated arthritis by activating the vagus nerve and enhancing acetylcholine (ACh) synthesis and transport via α7 nAChR-dependent cholinergic anti-inflammatory signaling along the gut–brain axis.

			ts	0.01); IL-10 and TGF-β significantly increased ($P < 0.05–0.01$).<br>6. Vagotomy abolished the increase in ChAT and VAChT and reverse d curcumin's anti-inflammatory effects.<br>7. Curcumin (10–30 μM) significantly increased neuronal excitability i n vagal nodose ganglion neurons ($P < 0.05–0.01$).<br>8. α7 nicotinic acetylcholine receptor (α7 nAChR) antagonists (α-bun garotoxin, methyllycaconitine) completely blocked these effects ($P < 0.01$).	
Lim 2023[47]	Scopolamine-indu ced cognitive im pairment in spon taneously hypert ensive rats (SHR/ Izm)	Male SHR/Izm rats, 6 we eks old (n = 40, 8/group)	Curcumin 100 or 200 m g·kg^{-1} orally, daily × 4 weeks; with scopolamine 1 mg·kg^{-1} i.p. (days 15–29); compared to tacrine 10 mg·kg^{-1} positive con trol	1. Systolic and diastolic blood pressure significantly decreased in cur cumin 200 mg·kg^{-1} group on day 29 ($P < 0.05$).<br>2. Y-maze alternation behavior significantly increased by curcumin (1 00–200 mg·kg^{-1}) compared to scopolamine group ($P < 0.05$).<br>3. Passive avoidance latency time significantly increased in curcumin-treated groups ($P < 0.05$).<br>4. Angiotensin II concentration in brain significantly decreased in cur cumin 200 mg·kg^{-1} group ($P < 0.05$).<br>5. mRNA expression of angiotensin-converting enzyme (ACE) and ang iotensin II receptor type 1 (AT1) significantly decreased in brain ($P < 0.05$).<br>6. Acetylcholine (ACh) concentration significantly increased in curcum in 200 mg·kg^{-1} group ($P < 0.05$).<br>7. mRNA expression of muscarinic acetylcholine receptor subtype 1 (m1AChR) and subtype 2 (m2AChR) significantly increased in curcu min-treated groups ($P < 0.05$).	Curcumin improved bloo d pressure and cognitiv e performance by suppr essing brain renin–angio tensin system (RAS) acti vity and enhancing cent ral cholinergic signaling through upregulation of acetylcholine (ACh) and muscarinic receptor expr ession.
Öz 2024[48]	Lipopolysaccharid e (LPS)-induced neuroinflammatio n and cognitive i mpairment (sepsi s model)	Male Swiss Albino mice (10–12 weeks old, n = 4 0, 8/group)	Adalimumab (ADA) 10 m g·kg^{-1} i.p., 3 doses ever y 5 days (pre- and co-tr eatment with LPS); LPS 0.5 mg·kg^{-1} i.p. for 7 da ys; Curcumin 50 mg·kg^{-1} orally for last 2 days (positive control)	1. Anxiety-like behaviors (open-field central zone time and rearing) s ignificantly decreased with LPS ($P < 0.001$); significantly increased by ADA and curcumin ($P < 0.001$).<br>2. Depression-like behavior significantly increased by LPS ($P < 0.001$); significantly reduced by ADA and curcumin ($P < 0.001$).<br>3. Cognitive performance (novel object discrimination index) significa ntly decreased by LPS ($P<0.001$); restored by ADA and curcumin ($P < 0.001$).<br>4. Serum and hippocampal tumor necrosis factor-α (TNF-α) and nitri c oxide (NO) levels significantly increased with LPS ($P < 0.001$); n ormalized by ADA ($P < 0.001$).<br>5. Brain-derived neurotrophic factor (BDNF) and cyclic AMP response element-binding protein (CREB) levels significantly decreased by L PS ($P < 0.001$); significantly increased by ADA and curcumin ($P < 0.001$).<br>6. Hippocampal acetylcholinesterase (AChE) activity significantly increa sed after LPS ($P < 0.001$); significantly decreased by ADA and cur cumin ($P < 0.001$).<br>7. Acetylcholine (ACh) content significantly decreased after LPS ($P < 0.001$); significantly increased by ADA ($P < 0.001$).	Adalimumab prevented LPS-induced neuroinflam mation and cognitive im pairment by inhibiting T NF-α and nitric oxide, r estoring brain-derived n eurotrophic factor (BDN F) and acetylcholine (AC h) homeostasis, and nor malizing cholinergic and neurotrophic signaling.

Yang 2023[49]	Bisphenol AF (BPAF)-induced neurodevelopmental toxicity	Zebrafish larvae (Danio rerio, AB strain; 60 embryos per group)	BPAF 200 μg·L⁻¹ ± Curcumin (200, 600, or 1,200 μg·L⁻¹) co-exposure from 5 to 96 h post-fertilization (hpf)	1. Locomotor activity (total distance and average speed) significantly decreased by BPAF (*P < 0.01*); significantly increased by curcumin 600–1,200 μg·L⁻¹ (*P < 0.05–0.01*).<br>2. Ballooning degeneration, dilated ventricles, and reduced brain tissue mass significantly improved with curcumin co-treatment.<br>3. Expression of neurogenesis-related genes elavl3, zn5, α-tubulin, syn2a, and mbp significantly decreased by BPAF (*P < 0.001–0.01*); significantly increased by curcumin (*P < 0.001–0.01*).<br>4. Growth-associated protein gap43 significantly upregulated by BPAF (*P<0.0001*); significantly downregulated by curcumin (*P < 0.0001*).<br>5. Acetylcholinesterase (AChE) activity significantly decreased by BPAF (0.49-fold, *P < 0.01*); significantly increased by curcumin (1.97-fold, *P < 0.05*).<br>6. Glutathione (GSH), superoxide dismutase (SOD), catalase (CAT), glutathione peroxidase (GPx), and glutathione S-transferase (GST) activities significantly decreased by BPAF (*P < 0.05–0.01*); significantly increased by curcumin (*P < 0.05–0.001*).<br>7. Malondialdehyde level significantly increased by BPAF (1.57-fold, *P <0.001*); significantly decreased by curcumin (0.42-fold, *P<0.05*).<br>8. Pro-apoptotic genes bax, caspase-9, and caspase-3 significantly increased (*P < 0.0001–0.01*); anti-apoptotic bcl-2 significantly decreased (*P < 0.0001*); all normalized by curcumin (*P < 0.001–0.01*).<br>9. Pro-inflammatory cytokines IL-6, IL-8, IL-1β, and TNF-α significantly increased by BPAF (*P < 0.001–0.0001*); significantly decreased by curcumin (*P < 0.01–0.0001*).	Curcumin protected against BPAF-induced neurotoxicity by restoring acetylcholinesterase (AChE) activity, enhancing antioxidant defense, and suppressing apoptosis and inflammation to maintain normal neurodevelopment.
Wu 2017[50]	Surgery-induced postoperative cognitive dysfunction (POCD)	Aged male ICR mice (12 months old; 30–40 g; n= 10/group)	Curcumin 50, 100, or 200 mg/kg/day p.o. for 32 days; exploratory laparotomy under fentanyl–droperidol anesthesia	1. Choline acetyltransferase (ChAT)-positive area significantly decreased after surgery (P < 0.01) and significantly increased by curcumin 100–200 mg/kg (P < 0.01).<br>2. Brain-derived neurotrophic factor (BDNF)-positive area significantly decreased after surgery (P < 0.01) and significantly increased by curcumin 50–200 mg/kg (P < 0.01).<br>3. Phosphorylated Akt (pAkt) expression significantly decreased after surgery (P < 0.05) and significantly increased by curcumin 50–200 mg/kg (P < 0.01).<br>4. Glutathione peroxidase activity significantly decreased after surgery (P < 0.05) and significantly increased by curcumin 200 mg/kg (P < 0.05).<br>5. Superoxide dismutase (SOD) activity significantly decreased after surgery (P < 0.05) and significantly increased by curcumin 100–200 mg/kg (P < 0.01).<br>6. Catalase (CAT) activity significantly decreased after surgery (P < 0.05) and significantly increased by curcumin 100–200 mg/kg (P < 0.05–0.01).	
Fikry 2021[51]	Parkinson's disease (PD) model in	Adult male albino rats (150–200 g; n = 40, 10/g	Rotenone 3 mg·kg⁻¹ i.p.	1. Rotarod performance time significantly decreased in rotenone group (*P<0.0001*); significantly increased with curcumin co-treatment	Curcumin prevented rotenone-induced Parkinson

	duced by rotenone neurotoxicity	roup)	daily × 60 days ± Curcumin 30 mg·kg⁻¹ i.p. daily × 60 days; Curcumin-only and vehicle controls	($P < 0.0001$).<br>2. Acetylcholinesterase (AChE) activity in cerebellum significantly decreased after rotenone ($P < 0.0001$); significantly increased after curcumin ($P < 0.0001$), restored to control level.<br>3. Malondialdehyde (MDA) level significantly increased after rotenone ($P < 0.0001$); significantly decreased after curcumin ($P < 0.0001$).<br>4. Glutathione (GSH) and superoxide dismutase (SOD) levels significantly decreased in rotenone group ($P < 0.0001$); significantly increased with curcumin ($P < 0.0001$).<br>5. Number of tyrosine hydroxylase (TH)-positive dopaminergic neurons in substantia nigra significantly decreased after rotenone ($P < 0.0001$); significantly increased by curcumin ($P < 0.0001$).<br>6. Number of glial fibrillary acidic protein (GFAP)-positive astrocytes significantly increased after rotenone ($P < 0.0001$); significantly decreased after curcumin ($P < 0.0001$).<br>7. Number of degenerated Purkinje cells in cerebellum significantly increased in rotenone group ($P < 0.0001$); significantly decreased after curcumin ($P < 0.0001$).	ian neurotoxicity by restoring cerebellar acetylcholinesterase (AChE) activity, reducing oxidative stress, and attenuating gliosis and Purkinje cell degeneration through antioxidant and cholinergic neuroprotective mechanisms.
Keshk 2020[52]	Streptozotocin (STZ)-induced type I diabetes–associated hippocampal neurodegeneration	Male Wistar rats (140–180 g; n = 28, 7/group)	STZ 65 mg·kg⁻¹ i.p. single dose; Curcumin 100 mg·kg⁻¹ orally, daily × 8 weeks post-STZ	1. Blood glucose level significantly increased in diabetic rats ($P < 0.05$); significantly decreased after curcumin ($P < 0.05$).<br>2. Malondialdehyde (MDA) and protein carbonyl contents significantly increased in diabetic group ($P < 0.05$); significantly decreased with curcumin ($P < 0.001$).<br>3. Total antioxidant capacity (TAC) significantly decreased in diabetic group ($P<0.05$); significantly increased after curcumin ($P < 0.001$).<br>4. Interferon-γ level significantly increased in hippocampus of diabetic rats ($P<0.05$); significantly decreased with curcumin ($P < 0.001$).<br>5. Heat shock protein 70 (HSP70) significantly decreased in diabetic hippocampus ($P < 0.05$); significantly increased after curcumin ($P < 0.01$).<br>6. Glucose-regulated protein 78 (GRP78), activating transcription factor 4 (ATF4), and CCAAT-enhancer-binding protein homologous protein (CHOP) levels significantly increased in diabetic hippocampus ($P < 0.01$); significantly decreased by curcumin ($P < 0.001$).<br>7. Bcl-2-associated X protein (Bax) significantly increased in diabetic rats ($P < 0.001$); significantly decreased by curcumin ($P < 0.001$).<br>8. B-cell lymphoma 2 (Bcl-2) immunoreactivity significantly decreased ($P < 0.001$); significantly increased after curcumin ($P < 0.001$).<br>9. Choline acetyltransferase (ChAT) immunoreactivity significantly decreased in diabetic group ($P < 0.001$); significantly increased by curcumin to near-control level ($P < 0.001$).<br>10. Histopathology: hippocampal pyramidal cell necrosis and shrinkage significantly improved after curcumin ($P = 0.0008$).	Curcumin mitigated diabetes-induced hippocampal injury by reducing oxidative and ER stress, inhibiting apoptosis, and restoring choline acetyltransferase (ChAT) expression, thus preserving cholinergic function and neuroprotection.
Lan 2018[53]	Stroke-prone spo	Male SHRsp rats (7 wee	Curcumin 100 mg·kg⁻¹·da	1. Onset of stroke significantly delayed ($P < 0.05$) and survival time	Curcumin prevented stro

	ntaneously hypertensive rats (SHRsp) – model of hypertension-induced stroke	ks old; n = 20, 10/group)	y^{-1} (oral gavage) for 4 weeks with 4% NaCl diet to induce stroke	significantly increased (*P < 0.05*).<br>2. Endothelium-dependent vasorelaxation to acetylcholine (ACh) in carotid artery significantly increased at 10^{-7}–10^{-5} M (*P<0.05–0.01*).<br>3. Endothelium-independent relaxation to sodium nitroprusside (SNP) significantly increased at 10^{-7}–10^{-6} M (*P < 0.05*).<br>4. Plasma nitrate/nitrite concentration significantly increased (*P<0.05*).<br>5. Plasma superoxide dismutase (SOD) activity significantly increased (*P < 0.05*).<br>6. Plasma malondialdehyde level significantly decreased (*P < 0.05*).<br>7. Reactive oxygen species (ROS) level in basilar artery significantly decreased (*P < 0.05*).<br>8. Nitric oxide (NO) fluorescence intensity in basilar artery significantly increased (*P < 0.05*).<br>9. Uncoupling protein 2 (UCP2) mRNA and protein expression in carotid artery significantly increased (*P < 0.01*).<br>10. Inhibition of UCP2 with genipin blocked curcumin-induced NO increase and ROS reduction (*P < 0.01*).	ke onset and improved endothelial function by enhancing acetylcholine (ACh)-mediated vasodilation, increasing nitric oxide (NO) bioavailability, and reducing oxidative stress through UCP2-dependent antioxidant signaling.
Abdel-Diam 2018[54]	Diazinon (DZN)-induced neurohepatic oxidative and inflammatory toxicity	Adult male albino rats (180 ± 20 g; n = 40, 8/group)	DZN 20 $mg·kg^{-1}$ orally × 28 days ± Curcumin 5 $mL·kg^{-1}$ and/or Quercetin 100 $mg·kg^{-1}$ orally, 1 h before DZN	1. Acetylcholinesterase (AChE) activity significantly decreased in serum (0.53-fold, *P < 0.05*) after DZN; significantly increased by curcumin (1.76-fold) and quercetin (1.59-fold), fully restored by combined treatment (*P < 0.05*).<br>2. Serum tumor necrosis factor-α (TNF-α) significantly increased (3.72-fold, *P < 0.05*) after DZN; significantly decreased by curcumin (0.34-fold) and quercetin (0.39-fold), normalized by combination (*P < 0.05*).<br>3. Serum liver enzymes (ALT, AST, ALP, LDH, γ-GT) significantly increased (*P < 0.05*) after DZN; significantly decreased by curcumin or quercetin, restored to control levels by combination (*P < 0.05*). 4<br>4. Malondialdehyde (MDA) level significantly increased in liver (2.38-fold) and brain (2.58-fold) (*P < 0.05*); significantly decreased by curcumin (0.40-fold) and quercetin (0.30-fold).<br>5. Glutathione (GSH), superoxide dismutase (SOD), glutathione peroxidase (GPx), and catalase (CAT) significantly decreased in liver and brain (*P < 0.05*); significantly increased after curcumin or quercetin (*P < 0.05*), maximally restored by combined treatment.	Combined curcumin and quercetin synergistically ameliorated diazinon-induced neurohepatic toxicity by restoring acetylcholinesterase (AChE) activity, reducing oxidative stress, and suppressing TNF-α–mediated inflammation via antioxidant and cholinergic pathways.
Song 2016[55]	Parkinson's disease (PD) model induced by 6-hydroxydopamine (6-OHDA) lesion of substantia nigra	Male Sprague-Dawley rats (8 weeks, 200 ± 10 g; n = 50, 10/group)	Curcumin 5, 10, or 20 $mg·kg^{-1}$ orally (intragastric), daily × 30 days after 6-OHDA lesion	1. Morris water maze and rotarod performance significantly improved by curcumin 10–20 $mg·kg^{-1}$ (*P < 0.05*).<br>2. Malondialdehyde (MDA) content in substantia nigra significantly decreased (*P < 0.05*).<br>3. Superoxide dismutase (SOD) and glutathione peroxidase (GSH-Px) activities significantly increased (*P < 0.05*).<br>4. Dopamine (DA) concentration significantly increased (*P < 0.05*).<br>5. Acetylcholine (ACh) level significantly increased (*P < 0.05*).<br>6. Neuronal necrosis in substantia nigra significantly reduced(*P<0.05*).	Curcumin improved motor and cognitive function in Parkinsonian rats by enhancing acetylcholine (ACh) and dopamine synthesis, increasing antioxidant enzyme activity, and promoting bFGF/NGF/TrkA/HSP70-mediated neurotrophic signaling in

				7. Basic fibroblast growth factor (bFGF), nerve growth factor (NGF), and tyrosine receptor kinase A (TrkA) expressions significantly increased ($P < 0.05$).<br>8. Heat shock protein 70 (HSP70) expression significantly increased ($P < 0.05$).	substantia nigra.
Akintunde 2019[56]	Bisphenol-A (BPA)-induced neuro-testicular dysfunction and Parkinson's disease-like neurotoxicity	Adult male Wistar rats (220–240 g; n = 40, 8/group)	BPA 50 mg·kg⁻¹ oral × 14 days ± Curcumin 50 or 100 mg·kg⁻¹ oral × 14 days	1. Number of rearings (locomotor activity) significantly decreased after BPA ($P < 0.05$); significantly increased after curcumin 50–100 mg·kg⁻¹ ($P < 0.05$).<br>2. Acetylcholinesterase (AChE) activity in brain significantly decreased after BPA ($P < 0.05$); significantly increased after curcumin 50–100 mg·kg⁻¹ ($P < 0.05$).<br>3. Nitric oxide (NO) concentration in brain significantly increased after BPA ($P < 0.05$); significantly decreased after curcumin ($P<0.05$).<br>4. Serum luteinizing hormone (LH), follicle-stimulating hormone (FSH), and testosterone significantly decreased after BPA ($P < 0.05$); significantly increased after curcumin 100 mg·kg⁻¹ ($P < 0.05$).<br>5. Daily sperm production, sperm motility, and sperm count significantly decreased after BPA ($P < 0.05$); significantly increased after curcumin 50–100 mg·kg⁻¹ ($P < 0.05$).<br>6. Total sperm deformity significantly increased after BPA ($P < 0.05$); significantly decreased after curcumin ($P < 0.05$).<br>7. Malondialdehyde (MDA) level in brain significantly increased after BPA ($P < 0.05$); significantly decreased after curcumin ($P < 0.05$).<br>8. Superoxide dismutase (SOD) and catalase (CAT) activities significantly decreased after BPA ($P < 0.05$); significantly increased after curcumin ($P < 0.05$).<br>9. Histopathology: BPA caused striatal congestion and testicular germ-cell loss; curcumin restored normal striatal and seminiferous architecture.	Curcumin prevented Bisphenol-A–induced Parkinsonian and reproductive dysfunction by restoring acetylcholinesterase (AChE) activity, reducing nitric oxide (NO) and oxidative stress, and normalizing hypothalamic–pituitary–gonadal hormone balance through antioxidant and cholinergic neuroendocrine regulation.
Blackshaw 2000[57]	Gastrointestinal vagal afferent activation model (normal physiology)	Adult ferrets (male and female, 0.5–1.5 kg; n = 63 fibres from 29 animals)	Acute capsaicin 1–3.25 mM topical or intra-arterial (32–164 nmol) application in vivo and in vitro	1. Capsaicin (3.25 mM intraluminal or 32–65 nmol intra-arterial) significantly increased firing frequency of 30% of gastrointestinal vagal afferents (P < 0.05) independent of motility changes.<br>2. In oesophagus, 2/10 tension and 1/1 mucosal receptors significantly increased discharge after capsaicin.<br>3. In stomach and duodenum, 5/14 tension and 2/4 mucosal receptors significantly increased firing (P < 0.05).<br>4. In vitro, 3/5 tension, 4/21 mucosal, and 2/8 tension/mucosal receptors significantly increased discharge (P < 0.05).<br>5. Capsaicin-induced excitation desensitized 37% of responsive afferents upon repeated exposure.<br>6. Cross-desensitization significantly reduced mechanical sensitivity in both responsive and non-responsive fibres (P < 0.05).<br>7. Blood pressure transiently increased by 8.5 ± 2.3 mmHg after oesophageal capsaicin infusion (P < 0.01).	Acute capsaicin directly activated approximately one-third of gastrointestinal vagal afferents, inducing excitation and desensitization independent of fibre type, suggesting heterogeneous vanilloid receptor expression mediating vagal sensory modulation.

Jancsó 1983[58]	Capsaicin-induced vagal afferent modulation of cardiovascular and respiratory reflexes	Adult cats (urethane or pentobarbital anesthesia; $n = 30$)	Perineural capsaicin (0.001–1.0%, 30 min) applied to cervical vagus nerves; acute and chronic experiments (3–5 days post-treatment)	1. Mean arterial blood pressure significantly decreased from 103 ± 8 to 69 ± 6 mmHg after capsaicin ($P < 0.01$).<br>2. Heart rate significantly decreased in 71% of experiments; magnitude correlated inversely with baseline rate ($r = -0.66$, $P < 0.001$).<br>3. Respiratory amplitude transiently decreased for 10–15 sec post-application; frequency variably increased (39%) or decreased (56%) ($P < 0.05$).<br>4. Intravenous capsaicin, phenyldiguanidine, and veratrine induced reflex bradycardia, hypotension, and apnoea; these reflexes were completely abolished by perineural capsaicin ($P < 0.01$).<br>5. Recovery of chemo-reflexes occurred 60–120min after removal of capsaicin cuffs.<br>6. In chronic experiments (3–5 days), reflex effects of PDG and veratrine remained intact, but capsaicin-induced bradycardia, hypotension, and apnoea were significantly reduced or abolished ($P<0.01$).<br>7. Capsaicin-treated animals displayed a secondary pressor response to intravenous capsaicin, indicating desensitization of capsaicin-sensitive vagal afferents.	Perineural capsaicin selectively desensitized non-myelinated vagal afferents mediating capsaicin-induced cardiovascular and respiratory chemo-reflexes, revealing distinct peptide-containing vagal fibre populations involved in autonomic regulation.
Mei 2013[59]	Cerebral ischemia–reperfusion injury (MCAO model)	Adult male Sprague-Dawley rats (250–300 g; $n = 60$, 12/group)	Puerarin 18 $mg \cdot kg^{-1}$ i.v. daily × 5 days; Nicotine 200 $\mu g \cdot kg^{-1}$ i.v. daily × 5 days; α-Bungarotoxin (α-BGT, 1 $\mu g \cdot kg^{-1}$) injected i.v. at reperfusion onset; ischemia for 2 h followed by 24 h reperfusion	1. Cerebral infarct area significantly decreased in puerarin ($P < 0.01$) and nicotine ($P < 0.05$) groups versus model; significantly increased in α-BGT group ($P < 0.05$).<br>2. mRNA expression of α7 nicotinic acetylcholine receptor (α7nAChR) significantly increased in puerarin ($P < 0.01$), α-BGT ($P < 0.05$), and nicotine ($P < 0.05$) groups versus model.<br>3. mRNA expression of signal transducer and activator of transcription 3 (STAT3) significantly increased in puerarin ($P < 0.01$) and nicotine ($P < 0.01$) groups; significantly decreased by α-BGT versus puerarin ($P < 0.01$).<br>4. mRNA expression of NF-κBp65 significantly decreased in puerarin ($P<0.01$) and nicotine ($P<0.05$) groups versus model; significantly increased in α-BGT versus puerarin ($P < 0.01$).	Puerarin reduced cerebral infarction and neuroinflammation by upregulating α7 nicotinic acetylcholine receptor (α7nAChR) and STAT3 and downregulating NF-κB p65, indicating activation of the vagal cholinergic anti-inflammatory pathway.
Wang 2012[60]	Cerebral ischemia–reperfusion injury (MCAO model)	Male Sprague-Dawley rats (250–300 g; $n = 130$, 5 groups)	Puerarin 18 $mg \cdot kg^{-1}$ i.v. daily × 5 days; Nicotine 200 $\mu g \cdot kg^{-1}$ i.v. daily × 5 days; α-Bungarotoxin (α-BGT, 1 $\mu g \cdot kg^{-1}$ i.v.) injected at reperfusion	1. Neurological deficit score significantly decreased in puerarin ($P < 0.05$) and nicotine ($P < 0.05$) groups; significantly increased by α-BGT ($P < 0.05$).<br>2. Histology: inflammatory cell infiltration in hippocampal ischemic area significantly reduced by puerarin and nicotine; significantly increased by α-BGT.<br>3. Interleukin-1β (IL-1β), interleukin-6 (IL-6), and tumor necrosis factor-α (TNF-α) levels significantly increased after ischemia ($P < 0.01$); significantly decreased by puerarin ($P < 0.01$) and nicotine ($P < 0.05$); significantly increased by α-BGT versus puerarin ($P < 0.01$).<br>4. Anti-inflammatory interleukin-10 (IL-10) level significantly decreased after ischemia ($P < 0.05$); significantly increased by puerarin ($P < 0.05$) and nicotine ($P < 0.05$); significantly decreased by α-BGT versus puerarin ($P < 0.05$).<br>5. α-BGT blocked all neuroprotective and anti-inflammatory effects o	Puerarin protected against cerebral ischemia–reperfusion injury by activating the vagal cholinergic anti-inflammatory pathway, increasing acetylcholine (ACh)-mediated α7 nicotinic acetylcholine receptor (α7nAChR) signaling, and suppressing pro-inflammatory cytokines (IL-1β, IL-6, TNF-α).

				f puerarin.	
Kaboutari 2024[61]	Thioacetamide (TA)-induced hepatic encephalopathy	Male Wistar rats (200–220 g; n = 40, 10/group)	TA 200 mg·kg⁻¹ i.p. twice/week × 4 weeks ± Menthol 10 mg·kg⁻¹ i.p. daily × 4 weeks	1. Serum ammonia and liver enzyme (AST, ALT, ALP, GGT) levels significantly increased in TA group (*P < 0.001*); significantly decreased with menthol (*P < 0.001*).<br>2. Malondialdehyde (MDA) level significantly increased in liver and hippocampus (*P < 0.01–0.001*); significantly decreased with menthol (*P < 0.05–0.01*).<br>3. Glutathione (GSH), superoxide dismutase (SOD), and glutathione peroxidase (GPx) activities significantly decreased in TA group (*P < 0.05–0.001*); significantly increased with menthol (*P < 0.05–0.01*).<br>4. Hippocampal tumor necrosis factor-α (TNF-α), interleukin-1β (IL-1β), and interleukin-6 (IL-6) mRNA expression significantly increased after TA (*P < 0.001*); significantly decreased with menthol (*P < 0.05–0.001*).<br>5. Toll-like receptor 4 (TLR4), myeloid differentiation factor 88 (MyD88), and nuclear factor-kappa B (NF-κB) expression significantly increased after TA (*P < 0.01*); significantly decreased with menthol (*P < 0.01–0.001*).<br>6. Acetylcholinesterase (AChE) mRNA and activity significantly increased after TA (*P < 0.001*); significantly decreased with menthol (*P < 0.001*).<br>7. Brain-derived neurotrophic factor (BDNF) and α7 nicotinic acetylcholine receptor (α7nAChR) expression significantly decreased after TA (*P< 0.01*); significantly increased with menthol (*P < 0.05–0.01*).<br>8. Percentage of cresyl violet–positive neurons in hippocampal CA1 and dentate gyrus significantly decreased after TA (*P < 0.01–0.001*); significantly increased with menthol (*P < 0.05–0.01*).<br>9. Astrocytic swelling and neuronal degeneration significantly reduced with menthol (*P < 0.01*).<br>10. Spatial learning latency in Morris water maze significantly increased after TA (*P<0.05*); significantly decreased with menthol(*P<0.05*).	Menthol ameliorated hepatic encephalopathy by suppressing TLR4/MyD88/NF-κB signaling, reducing acetylcholinesterase (AChE) expression and activity, and enhancing brain-derived neurotrophic factor (BDNF) and α7 nicotinic acetylcholine receptor (α7nAChR) expression, thereby improving antioxidant defense, neuroinflammation, and spatial memory.
Dou 2019[62]	Collagen-induced arthritis (CIA) – model of rheumatoid arthritis	Female Wistar rats (130–150 g; n = 6/group)	Madecassoside 30 mg·kg⁻¹ orally × 14 days (day 14–27) ± Atropine 1 mg·kg⁻¹, Hexamethonium 4 mg·kg⁻¹, Phentolamine 1 mg·kg⁻¹, or Propranolol 20 mg·kg⁻¹ i.p.; unilateral cervical vagotomy (Vgx) in separate cohort	1. Paw swelling and arthritis index (AI) scores significantly decreased with madecassoside (*P < 0.01*), comparable to leflunomide.<br>2. Co-administration with Atropine or Hexamethonium significantly reduced madecassoside's anti-arthritis efficacy (*P < 0.05–0.01*).<br>3. Co-administration with Phentolamine or Propranolol had no significant effect (*P > 0.05*).<br>4. Unilateral cervical vagotomy (Vgx) significantly attenuated madecassoside's therapeutic effect on paw swelling and AI scores (*P < 0.05–0.01*).<br>5. Histology: synovial hyperplasia, pannus formation, and inflammatory infiltration significantly decreased with madecassoside (*P < 0.01*); reductions abolished after vagotomy.<br>6. Serum tumor necrosis factor-α (TNF-α), interleukin-1β (IL-1β), interleukin-6 (IL-6), interferon-γ (IFN-γ), and interleukin-17A (IL-17A) significantly decreased after madecassoside (*P < 0.05–0.01*).	Madecassoside exerted anti-arthritic effects by activating the peripheral cholinergic anti-inflammatory pathway through vagus nerve integrity and muscarinic and nicotinic acetylcholine receptor signaling, suppressing TNF-α, IL-1β, IL-6, IFN-γ, and IL-17A.

				7. Serum interleukin-10 (IL-10) and transforming growth factor-β (TGF-β) significantly increased (*P < 0.05–0.01*).<br>8. Vagotomy abolished madecassoside's suppression of pro-inflammatory cytokines but did not affect regulation of T cell–related cytokines (*P > 0.05*).	
Lu 2018[63]	Lipopolysaccharide (LPS)-induced acute lung injury (ALI)	Male BALB/c mice (18–22 g; n = 8/group) and RAW 264.7 macrophage cells	3-Dehydroandrographolide (3-DA) 5 or 10 mg·kg^{-1} i.p. daily × 3 days before LPS 3 mg·kg^{-1} intratracheal; in vitro: 3-DA 2.5–10 μM ± α7 nicotinic acetylcholine receptor (α7nAChR) inhibitor methyllycaconitine (MLA, 80 μM) or α7nAChR siRNA	1. TNF-α and IL-6 release significantly increased after LPS (*P < 0.01*); significantly decreased by 3-DA (2.5–10 μM, *P < 0.05–0.01*).<br>2. IκBα phosphorylation and degradation significantly increased after LPS (*P < 0.01*); significantly decreased by 3-DA (*P < 0.05–0.01*).<br>3. NF-κB p65 nuclear translocation significantly increased after LPS (*P < 0.01*); significantly inhibited by 3-DA (*P < 0.05–0.01*).<br>4. Phosphorylated Akt (Ser473) significantly increased after LPS (*P < 0.01*); significantly decreased by 3-DA (*P < 0.01*).<br>5. α7nAChR expression significantly increased by 3-DA (*P < 0.01*); effect blocked by MLA (*P < 0.01*).<br>6. In RAW 264.7 cells, 3-DA binding affinity to α7nAChR confirmed by surface plasmon resonance (K_D = 15.7 μM).<br>7. Inhibition of α7nAChR with MLA or siRNA significantly abolished 3-DA effects on TNF-α, IL-6, NF-κB, and p-Akt (*P < 0.05–0.01*).<br>8. In ALI mice, lung wet/dry ratio, bronchoalveolar lavage fluid (BALF) protein, TNF-α, IL-6, and immune cell infiltration significantly increased after LPS (*P < 0.01*); significantly decreased by 3-DA 5–10 mg·kg^{-1} (*P < 0.05–0.01*).<br>9. MLA abolished 3-DA-mediated reductions in cytokines, lung edema, and macrophage/neutrophil infiltration (*P < 0.05–0.01*).	3-Dehydroandrographolide (3-DA) alleviated LPS-induced lung inflammation by activating the cholinergic anti-inflammatory pathway via α7 nicotinic acetylcholine receptor (α7nAChR) activation and inhibiting NF-κB/Akt signaling, reducing TNF-α and IL-6 production.
Ai 2013[64]	Cerebral ischemia–reperfusion (CIR) injury	Male rabbits (n = 24; 6/group)	*Angelica sinensis* polysaccharides (ASP) 150 or 300 mg·kg^{-1} orally × 40 days before CIR induction	1. Relative cerebral blood flow (rCBF) and relative cerebral blood volume (rCBV) significantly increased in ASP-treated groups (*P < 0.01*); mean transit time (rMTT) and time to peak (rTTP) significantly decreased (*P < 0.01*).<br>2. Malondialdehyde (MDA) and nitric oxide (NO) levels in ischemic cerebral tissue significantly increased in CIR group (*P < 0.01*); significantly decreased after ASP treatment (*P < 0.01*).<br>3. Glutathione (GSH) level significantly decreased in CIR group (*P < 0.01*); significantly increased by ASP 150–300 mg·kg^{-1} (*P < 0.01*).<br>4. Activities of superoxide dismutase (SOD), catalase (CAT), glutathione peroxidase (GSH-Px), and glutathione reductase (GR) significantly decreased in CIR group (*P < 0.01*); significantly increased with ASP (*P < 0.01*).<br>5. Acetylcholine(ACh), Na^+,K^+-ATPase, Ca^{2+},Mg^{2+}-ATPase, and glucose levels significantly decreased after CIR (*P < 0.01*); significantly increased after ASP (*P < 0.01*).<br>6. Acetylcholinesterase (AChE) activity significantly increased after CIR (*P < 0.01*); significantly decreased with ASP treatment (*P <0.01*).<br>7. Histopathology: ischemic cortical damage and neuronal necrosis m	*Angelica sinensis* polysaccharides protected against cerebral ischemia–reperfusion injury by enhancing antioxidant defense and energy metabolism, increasing acetylcholine (ACh) and ATPase activities, and inhibiting acetylcholinesterase (AChE), indicating restoration of cholinergic and vascular function.

				arkedly reduced with ASP ($P < 0.01$).	
Ding 2018[65]	Myocardial ischemia–reperfusion (MIRI) injury	Adult Sprague-Dawley rats (220 ± 20 g; n = 40, 10/group)	Breviscapine (25 or 50 mg·kg⁻¹·day⁻¹, i.p.) for 7 days before ischemia–reperfusion; 30 min ligation of left anterior descending coronary artery followed by 2 h reperfusion	1. Myocardial infarct area significantly increased in ischemia–reperfusion (IR) group ($P < 0.01$); significantly decreased by breviscapine 25–50 mg·kg⁻¹ ($P < 0.05–0.01$).<br>2. Cardiomyocyte apoptosis index significantly increased in IR group ($P < 0.01$); significantly decreased by breviscapine 25–50 mg·kg⁻¹ ($P < 0.05–0.01$).<br>3. p65 protein expression significantly decreased after IR ($P < 0.01$); significantly increased with breviscapine 25–50mg·kg⁻¹($P<0.05–0.01$).<br>4. IκB-α protein expression significantly increased after IR ($P < 0.01$); significantly decreased with breviscapine ($P < 0.05–0.01$).<br>5. α7 nicotinic acetylcholine receptor (α7nAChR) protein expression significantly decreased in IR group ($P < 0.01$); significantly increased with breviscapine 25–50 mg·kg⁻¹ ($P < 0.05–0.01$).<br>6. Breviscapine effects were dose-dependent, with 50 mg·kg⁻¹ producing maximal infarct and apoptosis reduction.	Breviscapine pretreatment protected against myocardial ischemia–reperfusion injury by upregulating α7 nicotinic acetylcholine receptor (α7nAChR) and p65, and downregulating IκB-α via NF-κB–mediated cholinergic anti-inflammatory signaling, thereby inhibiting cardiomyocyte apoptosis.
Bai 2020[66]	Chronic obstructive pulmonary disease (COPD) induced by cigarette smoke and lipopolysaccharide (LPS)	Male Sprague-Dawley rats (n = 90; 15/group)	Trans-anethole (ANE) 100 mg·kg⁻¹ orally, daily × 4 weeks; α7 nicotinic acetylcholine receptor (α7nAChR) antagonist α-bungarotoxin (α-BGT) 1 μg·kg⁻¹ i.p., 30 min before ANE treatment	1. Forced vital capacity (FVC), forced expiratory volume at 0.1 and 0.3 s ($FEV_{0.1}$, $FEV_{0.3}$), and $FEV_{0.1}$/FVC and $FEV_{0.3}$/FVC ratios significantly decreased in COPD rats ($P < 0.05$); significantly increased by ANE ($P < 0.05$).<br>2. Lung histology: alveolar septal thickening and inflammatory infiltration significantly reduced by ANE ($P < 0.05$).<br>3. Acetylcholine (ACh) and acetylcholinesterase (AChE) levels in lung tissue significantly increased in COPD rats ($P < 0.05$); further increased by ANE ($P < 0.05$); significantly decreased by α-BGT ($P < 0.05$).<br>4. Interleukin-6 (IL-6) and tumor necrosis factor-α (TNF-α) levels significantly increased in COPD rats ($P < 0.05$); significantly decreased by ANE ($P< 0.05$); significantly increased again by α-BGT ($P<0.05$).<br>5. α7 nicotinic acetylcholine receptor (α7nAChR) mRNA and protein expression significantly increased by ANE ($P < 0.05$); significantly decreased by α-BGT ($P < 0.05$).<br>6. Janus kinase 2 (JAK2), signal transducer and activator of transcription 3 (STAT3), and nuclear factor kappa B (NF-κB) mRNA and protein expression significantly increased in COPD rats ($P < 0.05$); significantly decreased by ANE ($P < 0.05$); significantly increased again by α-BGT ($P < 0.05$).<br>7. Immunohistochemistry showed increased α7nAChR and decreased JAK2, STAT3, and NF-κB staining in ANE-treated lungs ($P < 0.05$).	Trans-anethole ameliorated COPD by enhancing α7 nicotinic acetylcholine receptor (α7nAChR) signaling and suppressing JAK2/STAT3/NF-κB-mediated inflammation through activation of the cholinergic anti-inflammatory pathway.

Supplementary Table 6. Summary of studies on vagal modulation by phytoconstituent derivatives and formulations

Study (Year)	Disease Model	Subjects	Intervention	Main Vagal Outcomes	Summary of findings
Khadrawy 2021[67]	Doxorubicin-induced neurotoxicity	Male Wistar rats (160–180 g; N=32)	Nanocurcumin (NC): 50 mg/kg p.o. for 14 days; Doxorubicin (DOX): single 20 mg/kg i.p. on day 10; NC + DOX: NC for 9 days before and 4 days after DOX	Cortex:<br>1. Acetylcholinesterase(AChE) activity significantly decreased by 94.82% (P < 0.05) after DOX.<br>2. Monoamine oxidase (MAO) activity significantly decreased by 64.40% (P < 0.05) after DOX.<br>3. Dopamine (DA) level significantly increased by 71.88% (P < 0.05) after DOX.<br>4. Malondialdehyde(MDA) significantly increased by 183.99%(P<0.05) after DOX.<br>5. Nitric oxide (NO) significantly increased by 36.54% (P<0.05) after DOX.<br>6. Reduced glutathione (GSH) significantly decreased by 13% (P<0.05) after DOX.<br>7. NC pretreatment significantly mitigated DOX-induced changes in MDA, NO, GSH, AChE, MAO, and DA (P < 0.05).<br>Hippocampus:<br>1. AChE activity significantly decreased by 62.75%(P<0.05) after DOX.<br>2. MAO activity significantly decreased by 68.84%(P<0.05) after DOX.<br>3. MDA significantly increased by 201.4% (P < 0.05) after DOX.<br>4. NO significantly increased by 55% (P < 0.05) after DOX.<br>5. GSH significantly decreased by 21.44% (P < 0.05) after DOX.<br>6. NC pretreatment significantly mitigated changes in MDA and NO (P < 0.05) but did not fully normalize GSH, AChE, or MAO.	Nanocurcumin exerted neuroprotective effects by significantly reducing oxidative stress and partially normalizing cholinergic and monoaminergic disruptions induced by doxorubicin.
Ma 2024[68]	D-galactose–induced brain aging	Male C57BL/6J mice, 7-months-old, ~ 30 g (n=8/group)	D-galactose 500 mg/kg/day i.p. × 10 weeks; COP-22 50, 100, 200 mg/kg/day p.o. × 10 weeks	1. Acetylcholine (Ach) level significantly decreased with D-gal (P < 0.001) and significantly increased by COP-22 (dose-dependent; P < 0.05–0.001).<br>2. 2. Acetylcholinesterase (AchE) activity significantly increased with D-gal (P < 0.001) and significantly decreased by COP-22 (50–200 mg/kg; P < 0.001).<br>3. Malondialdehyde (MDA) significantly increased with D-gal (P < 0.001) and significantly decreased by COP-22 (P < 0.05–0.001).<br>4. Catalase (CAT) significantly decreased with D-gal (P < 0.01) and significantly increased by COP-22 (P < 0.01–0.001).<br>5. Glutathione peroxidase (GPx) significantly decreased with D-gal (P < 0.01) and significantly increased by COP-22 (P < 0.05–0.01).<br>6. Superoxide dismutase (SOD) unchanged (ns).<br>7. Hippocampal CA1/CA3/DG structural disruption with D-gal improved by COP-22 (200 mg/kg; P < 0.05–0.001).<br>8. SIRT1 significantly decreased with D-gal (P < 0.01) and significantly increased by COP-22 (P < 0.05–0.01).<br>9. p53, p21, p16 significantly increased with D-gal (P < 0.001) and significantly decreased by COP-22 (P < 0.05–0.001).<br>10. IL-6 and TNF-α significantly increased with D-gal (P < 0.001) and significantly decreased by COP-22 (P < 0.05–0.001).<br>11. Bax and caspase-3 significantly increased with D-gal (P<0.001) and significantly decreased by COP-22 (P < 0.05–0.001).	COP-22 significantly improved cholinergic function and reduced oxidative stress, inflammation, and apoptosis in D-gal–induced brain aging via SIRT1/Nrf2-dependent pathways.
Noor 2022[69]	Intracerebroventricular streptozoto	Adult male Wistar rats (250–350 g; n	Curcumin nanoparticles (CNs) 50 mg/kg/d	1. Acetylcholinesterase (AChE) activity significantly increased by STZ (+149.04%) (P<0.05) and significantly decreased by CNs (P <0.05).	Curcumin nanoparticles significantly improve

	cin (STZ)-induced Alzheimer-like neurodegeneration	=10/group)	ay p.o. for 6 days before STZ + continued 8 days post-STZ; single i.c.v. STZ 3 mg/kg on day 7	2. Sodium–potassium ATPase (Na^+,K^+-ATPase) activity significantly decreased by STZ (P<0.05) and significantly attenuated by CNs (P<0.05).<br>3. AChE activity significantly increased by STZ (+120%) (P < 0.05) and significantly ameliorated by CNs (P < 0.05).<br>4. Na^+,K^+-ATPase activity significantly decreased by STZ (P < 0.05) and significantly improved by CNs (P < 0.05).<br>5. Malondialdehyde (MDA) significantly increased in cortex (+69.41%) and hippocampus (+85.27%) with STZ (P < 0.05) and significantly reduced by CNs (P < 0.05).<br>6. Nitric oxide (NO) significantly increased in cortex (+91.67%) and hippocampus (+96%) with STZ (P < 0.05); cortex restored to control by CNs.<br>7. Reduced glutathione (GSH) significantly decreased in cortex (−21.22%) and hippocampus (−12.11%) with STZ (P < 0.05) and significantly increased by CNs (P < 0.05).<br>8. Tumor necrosis factor-alpha (TNF-α) significantly increased in cortex (+140%) and hippocampus (+88.14%) (P < 0.05) and significantly decreased by CNs (P < 0.05).<br>9. Cortical γ-aminobutyric acid (GABA), glycine, taurine, glutamate, and aspartate significantly decreased with STZ (P < 0.05) and partially restored by CNs.<br>10. Hippocampal glutamate, aspartate, GABA, glutamine, glycine, and taurine significantly increased with STZ (P<0.05) and significantly reduced toward control by CNs.	d cholinergic and amino-acid neurotransmitter balance while reducing oxidative stress and inflammation in STZ-induced Alzheimer-like neurodegeneration.
Shabbir 2022 [70]	Aluminum chloride ($AlCl_3$)–induced Alzheimer-type neurodegeneration	Male albino Wistar rats (200–250 g; n = 6/group)	$AlCl_3$ 300 mg/kg/day p.o. × 21 days; treatments (days 1–21): rivastigmine 1 mg/kg; curcuminoids crude extract (CR-30), curcuminoid-rich extract (CRE-30), or ternary complex (TC-30), each 30 mg/kg/day p.o.	1. Acetylcholinesterase (AChE) activity significantly increased with $AlCl_3$ (P < 0.05) and significantly decreased by CRE-30 and TC-30 (P < 0.05).<br>2. AChE mRNA expression significantly increased (2.86-fold) with $AlCl_3$ (P < 0.05) and significantly decreased by CRE-30 (1.53-fold) and TC-30 (1.36-fold) (P < 0.05).<br>3. Malondialdehyde (MDA) significantly increased with $AlCl_3$ (P<0.05) and significantly decreased by CRE-30 and TC-30 (P < 0.05).<br>4. Catalase (CAT) significantly decreased with $AlCl_3$ (P < 0.001) and significantly increased by CRE-30 and TC-30 (P < 0.05).<br>5. Superoxide dismutase (SOD) significantly decreased with $AlCl_3$ (P < 0.05) and significantly increased by CRE-30 and TC-30 (P<0.05).<br>6. Glutathione peroxidase (GPx) significantly decreased with $AlCl_3$ (P < 0.05) and significantly increased by CRE-30 and TC-30 (P<0.05).<br>7. Interleukin-6 (IL-6) significantly increased with $AlCl_3$ (P<0.05) and significantly decreased by CRE-30 and TC-30 (P < 0.05).<br>8. Tumor necrosis factor-alpha (TNF-α) significantly increased with $AlCl_3$ (P < 0.05) and significantly decreased by CRE-30 and TC-30 (P < 0.05).<br>9. IL-1α mRNA expression significantly increased with $AlCl_3$ (2.76-fold; P < 0.05) and significantly decreased by CRE-30 and TC-30 (1.27–1.13-fold; P < 0.05).<br>10. IL-1β mRNA significantly increased with $AlCl_3$ (2.93-fold; P < 0.05) and significantly decreased by CRE-30 and TC-30 (1.53–1.16-fold; P < 0.05).<br>11. β-secretase (BACE-1) mRNA expression significantly increased with$AlCl_3$ (3.00-fold; P < 0.05) and significantly decreased by CRE-30 (1.23-fold) and TC-30 (1	CRE-30 and TC-30 significantly improved cholinergic status and reduced oxidative stress, inflammation, and AD-related gene expression, indicating strong neuroprotective activity against $AlCl_3$-induced neurodegeneration.

				.06-fold) (P < 0.05).<br>12. $AlCl_3$-induced neuronal loss, neurofibrillary tangles, pigmentation, and plaques were markedly reduced in CRE-30 and TC-30 groups (H&E; pgs. 12–13).	
Guo 2018[71]	Scopolamine-induced learning and memory impairment	Male KM mice (18 ± 2 g; n varies by assay)	Bisdemethoxycurcumin (BDMC), curcumin (Cur), demethoxycurcumin (DMC), or tetrahydrocurcumin (THC) solid dispersions 200 mg/kg/day p.o. × 35 days; additional THC dose-response (100–400 mg/kg/day)	1. Choline acetyltransferase (ChAT) significantly decreased with scopolamine (P < 0.01) and significantly increased by BDMC, Cur, DMC, and THC (P < 0.05–0.01).<br>2. Acetylcholinesterase (AChE) significantly increased with scopolamine (P < 0.05) and significantly decreased by BDMC, Cur, and DMC (P < 0.05–0.01); THC significantly increased ChAT (P < 0.01) and mildly decreased AChE.<br>3. Acetylcholine (ACh) level decreased with scopolamine and was modestly increased by BDMC, DMC, and Cur (non-uniform significance across compounds).<br>4. AChE significantly decreased at 200–400 mg/kg THC (P < 0.05).<br>5. ChAT significantly increased at 400 mg/kg THC (P < 0.05).<br>6. Malondialdehyde (MDA) significantly increased with scopolamine (P < 0.01) and significantly decreased by THC 100–400 mg/kg (P < 0.01).<br>7. Superoxide dismutase (SOD) significantly decreased with scopolamine (P<0.01) and significantly increased by THC400mg/kg (P<0.05).<br>8. Escape latency significantly increased with scopolamine (P < 0.01) and significantly decreased by all curcumin compounds (P < 0.05–0.01), with THC producing the greatest improvement.<br>9. Effective zone entries in probe test significantly decreased with scopolamine (P < 0.01) and significantly increased by all compounds (P < 0.01).	Curcumin-related solid dispersions, especially tetrahydrocurcumin, significantly improved cholinergic function and reduced oxidative stress, enhancing learning and memory in scopolamine-induced impairment.
Mohammed 2020[72]	Doxorubicin-induced cardiotoxicity	Male Wistar rats (160–180 g; n=9/group)	Curcumin nanoparticles (CUR-NPs) 50 mg/kg/day p.o. × 14 days; doxorubicin 20 mg/kg i.p. on day 10	1. Acetylcholinesterase (AChE) activity significantly increased by doxorubicin (+84.68%; P < 0.05) and significantly decreased toward control by CUR-NPs (P < 0.05).<br>2. Sodium–potassium ATPase (Na^+/K^+-ATPase) activity significantly decreased by doxorubicin (−33.33%; P < 0.05) and significantly increased by CUR-NPs (P < 0.05).<br>3. Serotonin (5-HT) significantly decreased by doxorubicin (−25.09%; P < 0.01) and significantly increased by CUR-NPs (P < 0.05).<br>4. Norepinephrine (NE) significantly decreased by doxorubicin (−25.06%; P < 0.001) and significantly increased by CUR-NPs (P< 0.05)<br>5. Dopamine (DA) significantly increased by doxorubicin (+199.45%; P<0.01) and significantly decreased by CUR-NPs (P<0.05).<br>6. Malondialdehyde (MDA) significantly increased with doxorubicin (+108.5%; P<0.001) and significantly decreased by CUR-NPs(P<0.05).<br>7. Nitric oxide (NO) significantly increased with doxorubicin (+200%; P < 0.001) and significantly decreased by CUR-NPs (P < 0.05).<br>8. Reduced glutathione (GSH) significantly decreased with doxorubicin (−23.31%; P < 0.01) and significantly increased by CUR-NPs (P < 0.05).<br>9. Serum lactate dehydrogenase (LDH) significantly increased by doxorubicin (+70.85%; P < 0.05) and significantly decreased by CUR-NPs (P < 0.05).<br>10. Doxorubicin significantly increased heart rate (+14%; P < 0.05), QRS duration (+27%; P < 0.05), QRS amplitude (+87.5%; P < 0.05), and ST duration (+22%; P < 0.05); CUR-NPs normalized all parameters toward control.	Curcumin nanoparticles significantly restored cholinergic activity, monoamines, and ionic pump function while reducing oxidative stress and ECG abnormalities, providing strong protection against doxorubicin-induced cardiotoxicity.

Supplementary Table 7. Summary of studies on vagal modulation by essential oils

Study (Year)	Disease Model	Subjects	Intervention	Main Vagal Outcomes	Summary of findings
Zhang 2008[73]	Restraint stress–induced delayed gastric emptying	Male Wistar rats (220–250 g)	Essential oil from Atractylodes lancea (30, 60, 120 mg/kg/day, oral, 7days)	1. Gastric emptying significantly increased in restraint-stressed rats ($P < 0.01$). Motilin (MTL) level significantly increased ($P < 0.01$).<br>2. Gastrin (GAS) level significantly increased ($P < 0.01$).<br>3. Somatostatin (SS) level significantly decreased ($P < 0.05$–0.01).<br>4. Corticotropin-releasing factor (CRF) significantly decreased in hypothalamus ($P < 0.05$–0.01).<br>5. Vagotomy abolished both stress-induced delay and EO effects, indicating vagal dependence.<br>6. CRF-induced inhibition of gastric emptying was significantly antagonized by EO ($P < 0.01$).	EO improves stress-induced delayed gastric emptying via inhibition of central CRF release and activation of vagal pathways.
Satou 2022[74]	Autonomic imbalance assessed during elevated platform exposure	Male ICR mice (≈30 g)	Inhalation of Pelargonium graveolens essential oil (5 µL/L air, 90 min)	1. Heart rate did not significantly change ($P > 0.05$).<br>2. Sympathetic nerve activity (LF/HF) significantly decreased ($P = 0.012$).<br>3. Parasympathetic nerve activity (HF%) significantly increased ($P = 0.022$).<br>4. Linalool detected in brain after intraperitoneal administration (≈339.9 ± 74.3 pg/g), confirming blood–brain barrier penetration.	Geranium essential oil shifts autonomic balance toward parasympathetic dominance via linalool-mediated central modulation.
Komiya 2009[75]	Autonomic imbalance assessed during repeated topical essential-oil exposure	5 healthy adults male Beagle dogs	Topical Lavandula angustifolia oil (0.18 mL/ear × 4 applications at 8:30, 12:00, 15:30, 19:00; single-day treatment)	1. Heart rate significantly decreased during 19:00–22:30 ($P < 0.05$).<br>2. High-frequency (HF) power significantly increased during 15:30–19:00 ($P < 0.05$).<br>3. Low-frequency to high-frequency ratio (LF/HF) did not significantly change ($P > 0.05$).<br>4. Circadian rhythm of heart rate variability remained intact across conditions.	Topical lavender oil transiently enhances vagal activity and reduces heart rate, indicating mild parasympathetic activation.

Supplementary Table 8. Characteristics, extraction methods, and phytochemical characterization of multi-herb prescriptions

Study (Year)	Intervention name	Botanical source	Plant part used	Extraction method	Extraction details	Major chemical constituents reported	Percentage/concentration reported	Chemical characterization performed	Analytical method	Botanical authentication method	Storage/stability reported?	Storage details
Wang 2011[1]	Banxia Baizhu Tianma Decoction	Pinellia ternata, Gastrodia elata, Poria cocos, Citrus reticulata, Atractylodes macrocephala, Glycyrrhiza uralensis	Rhizome (Pinellia), tuber (Gastrodia), sclerotium (Poria), pericarp (Citrus), rhizome (Atractylodes), root (Glycyrrhiza)	Water extraction	Concentrated aqueous decoction to 0.432 g crude drug/mL	NR	NR	NR	NR	Expert morphological identification; pharmacopoeial species confirmed by Prof. Ke Yan	NR	NR
Lu 2015[2]	Banxia Qinlian Decoction	Pinellia ternata, Scutellaria baicalensis, Coptis chinensis, Glehnia littoralis, Gypsum Fibrosum (Shigao), Paeonia lactiflora, Cornus officinalis	Rhizome (Pinellia), root (Scutellaria), rhizome (Coptis), root (Glehnia), mineral (Gypsum Fibrosum), root (Paeonia), fruit (Cornus)	Decoction (likely aqueous extraction)	Formula consisted of Banxia 9 g, Huangqin 9 g, Huanglian 9 g, Beishashen 15 g, Shigao 30 g, Baishao 9 g, Shanzhuyu 30 g; administered as 60 g crude drug/kg/day by gavage; concentration 3.03 g/mL	NR	NR	NR	NR	NR	NR	NR
Zhang 2005[3]	Banxia Xiexin Decoction	Pinellia ternata, Scutellaria baicalensis, Zingiber officinale, Panax ginseng, Glycyrrhiza uralensis, Coptis chinensis, Ziziphus jujuba	Rhizome (Pinellia), root (Scutellaria), rhizome (Zingiber), root (Panax), root (Glycyrrhiza), rhizome (Coptis), fruit (Ziziphus)	Water extraction (decoction)	Herbs were decocted twice for 30 min each, decoctions combined, filtered, concentrated to 90 mL, and stored in refrigerator; dosing based on conversion of human equivalent dose to rats	NR	NR	NR	NR	Authentication by the Department of Medicinal Plants, Beijing University of Chinese Medicine	Yes	Stored in refrigerator
Du 2007[4]	Baobaole oral liquid	Atractylodes lancea/Atractylodes chinensis, Crataegus pinnatifida, Astragalus membranaceus, Codonopsis pilosula, Citrus reticulata, Paeonia lactiflora, Cassia obtusifolia/C. tora, Dragon bone, Oyster shell	Rhizome, fruit, root, root, pericarp, root, seed, fossil bone, shell	NR	Oral liquid prepared by Xijing Hospital Pharmacy Department; administered by gavage for 3 weeks	NR	NR	NR	NR	NR	NR	NR
Xie 2015[5]	Dachengqi Decoction	NR in manuscript (classically contains Rheum palmatum, Magnolia officinalis, Citrus aurantium, Natrii Sulfas)	NR	NR	Supplied by Tianjin Nankai Hospital Pharmaceutical Factory; administered by gavage at 2 mL twice daily (1 g crude drug/mL) beginning 2 days before model induction	NR	1 g crude drug/mL	NR	NR	NR	NR	NR
Zhang	Er'bao Granule	Atractylodes lancea/	Rhizome, pericarp,	NR	Extract supplied by Fo	NR	1.5 g cru	NR	NR	NR	NR	NR

2005[6]		A. chinensis, Citrus reticulata, Crataegus pinnatifida, Paeonia lactiflora	fruit, root		urth Military Medical University Pharmaceutical Factory; each mL contained 1.5 g crude drug equivalent; administered by gavage for 3 weeks		de drug/mL					
Jiang 2016[7]	Guizhi Decoction	Cinnamomum cassia, Paeonia lactiflora, Glycyrrhiza uralensis, Zingiber officinale, Ziziphus jujuba	Twig, root, root, rhizome, fruit	Decoction	Herbs were decocted and concentrated to a final concentration of 1.5 g crude drug/mL; prepared by the preparation room of the affiliated hospital	NR	1.5 g crude drug/mL	No	NR	NR	No	NR
Li 2014[8]	Guizhi Decoction	Cinnamomum cassia, Paeonia lactiflora, Glycyrrhiza uralensis, Zingiber officinale, Ziziphus jujuba	Twig, root, root, rhizome, fruit	Decoction	Classical Guizhi decoction administered by gavage	NR	NR	No	NR	Herbs identified by Prof. Feng Li, College of Pharmacy, Shandong University of Traditional Chinese Medicine	No	NR
Li 2015[9]	Guizhi Decoction	Cinnamomum cassia, Paeonia lactiflora, Glycyrrhiza uralensis, Zingiber officinale, Ziziphus jujuba	Twig, root, root, rhizome, fruit	Decoction	Rats were pretreated for 1 week before STZ induction and treated for 4 additional weeks	NR	NR	No	NR	NR	No	NR
Sun 2022[10]	Houpo-Mahuang Decoction	Magnolia officinalis, Ephedra intermedia, Gypsum fibrosum, Prunus armeniaca, Zingiber officinale, Asarum heterotropoides, Schisandra chinensis, Pinellia ternata, Triticum aestivum	Bark, stem, mineral, seed, rhizome, root/rhizome, fruit, tuber, seed	Water extraction (decoction); additional ethanol extraction for fractionation	Herbs soaked in 10× water for 30 min, reflux extracted twice, concentrated to 1.2 g/mL; additional extraction using 75% ethanol followed by n-butanol and ethyl acetate partitioning	Ephedrine, pseudoephedrine, laetrile, amygdalin amide; 51 chromatographic peaks identified	NR	Yes	UPLC-QTOF-MS	Voucher specimen deposited; authenticated by Prof. Weidong Li, School of Pharmacy, NJUCM	NR	NR
Sun 2025[11]	Liangxue Tongyu Prescription	Rheum officinale, Bubali cornu, Rehmannia glutinosa, Paeonia veitchii, Paeonia suffruticosa, Acorus calamus, Pheretima, Panax notoginseng	Rhizome, horn, root, root, root bark, rhizome, whole animal, root	Water decoction	Herbs soaked in 10 volumes water for 30 min; Bubali cornu boiled first for 20 min; other herbs decocted at 100°C for 20 min; Rheum officinale added during final 10 min; extraction repeated twice and concentrated to 1.2 g/mL	Taurine, Paeonol, Ginsenoside Rb1, Geniposide, Ginsenoside Rg1 discussed in manuscript	Not reported	Yes	UHPLC-QE-MS	NR	NR	NR
Zhang 2011[12]	Liu Wei Dihuang	Rehmannia glutinosa, Cornus officinalis, Dioscorea opposita, Alisma orientalis, Pa	Root, fruit, rhizome, rhizome, root bark, sclerotium	Water decoction	Herbs mixed in ratio 8:4:4:3:3:3, filtered, concentrated and sterilized; final concentration	NR	0.5 g/mL final extract concentration on	No	NR	Pharmacognostic identification before preparation	Yes	Stored at 4°C

		eonia suffruticosa, Poria cocos			0.5 g/mL		ly					
Bai 2019[13]	Pei Yuan Tong Nao	Fallopia multiflora, Rehmannia glutinosa, Asparagus cochinchinensis, Cistanche deserticola, Cinnamomum cassia, Paeonia lactiflora, Paeonia veitchii, Crataegus pinnatifida, Poria cocos, Glycyrrhiza uralensis, plus animal-derived materials (Carapax Testudinis, Cornu cervi pantotrichum, Buthus martensii, Hirudo nipponia, Lumbricus spp.)	Root, root, root, stem, bark, root, root, fruit, sclerotium, root, animal materials	Manufacturer-prepared dry extract powder reconstituted in water	Dry extract powder supplied by Henan Lingrui Pharmaceutical Co., Ltd.; water added immediately before gavage; batch no. 1709192; administered at 0.486 g/kg	NR	NR	Yes	HPLC fingerprinting	NR	NR	NR
Nakyam 2023[14]	GCJ polyherbal	Zingiber officinale Roscoe, Ziziphus jujuba Mill., Auricularia auricula-judae	Rhizome, fruit, fruiting body	Aqueous extraction	Aqueous extracts mixed in a 1:1:1 ratio after authentication of raw materials	6-Gingerol, 6-Shogaol, Catechin, Chlorogenic acid, Caffeic acid	36.981 ± 0.62, 8.558 ± 0.19, 0.137 ± 0.04, 0.042 ± 0.007, and 0.029 ± 0.001 µg/50 mg extract, respectively	Yes	HPLC	Authentication process performed before preparation (method not specified)	NR	NR
Jiang 2013[15]	Qi'ao Decoction	Ephedra sinica (Herba Ephedrae), Prunus armeniaca (Semen Armeniacae Amarum), Glycyrrhiza uralensis (Radix et Rhizoma Glycyrrhizae), Pinellia ternata (Rhizoma Pinelliae), Schisandra chinensis (Fructus Schisandrae Chinensis), Gypsum Fibrosum, Green tea (Camellia sinensis)	Stem, seed, root/rhizome, rhizome, fruit, mineral, leaf	Water decoction	Herbs decocted and concentrated to 2.68 g crude drug/mL	NR	2.68 g crude drug/mL	No	NR	Botanical identification by Prof. Wu Dekang, Department of Medicinal Plants, Nanjing University of Chinese Medicine	No	NR
Gan 2023[16]	Shegan Zhike Capsule	Belamcanda chinensis (L.) DC. (Belamcandae Rhizoma, Shegan)	Rhizome	Modern extraction, separation and purification process	Extract prepared from Belamcandae Rhizoma using proprietary extraction, separation and purification methods; final preparation formulated as capsule; batch no. 20200402	NR	Doses reported as extract equivalent: 26–172 mg extract/kg	NR	NR	NR	NR	NR

Wu 2019[17]	Shenmayizhi D ecoction	Panax ginseng (Radi x Ginseng), Gastrodi a elata (Rhizoma G astrodiae), Euonymu s alatus (Ramuli Eu onymi), Ligusticum c huanxiong (Rhizoma Chuanxiong)	Root, rhizome, twi g, rhizome	Water extr action und er reflux	33 g crude herbs soak ed in distilled water (1:10, w/v) for 30 min and extracted twice u nder reflux at 100°C f or 2 h each; extracts combined and freeze-d ried; 5 g lyophilized p owder obtained from 33 g crude herbs	Gastrodin, Ferulic acid , Ginsenosi de Rg1, Gi nsenoside Rb1, Querc etin	Standards used: gast rodin 300 µg/mL; fe rulic acid 500 µg/m L; ginseno side Rg1 1 mg/mL; ginsenosid e Rb1 1 mg/mL; q uercetin 1 mg/mL	Yes	HPLC	NR	Yes	Lyophilize d powder stored at 4°C befor e use
Wu 2015[18]	Sini Decoction	Aconitum carmichael ii (processed lateral root, Fuzi), Glycyrrhi za uralensis (Zhigan cao), Zingiber officin ale (Ganjiang), Cinn amomum cassia (Gu izhi), Cornus officina lis (Shanzhuyu), Pori a cocos (Fuling)	Processed lateral r oot, root, rhizome , twig, fruit, scler otium	Water dec oction	Herbs soaked in distill ed water, decocted fo r 30 min, filtered thro ugh 100-mesh stainles s steel sieve and conc entrated to final conc entrations of 0.972 an d 1.944 g crude drug/ mL	NR	0.972 and 1.944 g crude dru g/mL	No	NR	NR	NR	NR
Qi 2014[19]	WenYangTongz hi	Contains processed Aconitum carmichael ii (Fuzi), Cinnamomu m cassia (Guizhi), A tractylodes macroce phala (Baizhu), Epim edium spp. (Yinyang huo), deer antler fr ost, Psoralea corylif olia (Buguzhi), Nard ostachys jatamansi (Gansong), Rehmanni a glutinosa (Shudihu ang), Salvia miltiorr hiza (Danshen), Pan ax notoginseng (San qi), Asarum spp. (Xi xin), Astragalus me mbranaceus (Huang qi)	Multiple	Not report ed	Capsule contents disso lved in normal saline to prepare 50 and 10 0 mg/mL solutions for administration	NR	50 mg/mL and 100 mg/mL fo rmulations	No	NR	NR	No	NR
Ma 2016[20]	Yiqihuoxue Co mpound	Includes Astragalus membranaceus (Hua ngqi), Panax ginseng (Renshen), Cartham us tinctorius (Hongh ua), Salvia miltiorrhi za (Danshen), Leonu rus japonicus (Yimuc	Multiple	Water dec oction	Herbs decocted and c oncentrated to a final relative density of 1.0 5–1.08	NR	Relative d ensity 1.0 5–1.08	No	NR	NR	No	NR

		ao), Panax notoginseng (Sanqi), Lepidium apetalum (Tinglizi) and others										
Du 2006[21]	Yunpi Prescription	Atractylodes lancea (Cangzhu), Citrus reticulata (Chenpi), Crataegus pinnatifida (Shanzha), Paeonia lactiflora (Baishao)	Rhizome, pericarp, fruit, root	Aqueous extract	Granule extract paste supplied by institutional pharmaceutical factory; contained 1.5 g crude drug per mL; administered at 37.6 g/kg (twice the clinical equivalent dose)	NR	1.5 g crude drug/mL extract; dose 37.6 g/kg	No	NR	NR	No	NR
Tan 2017[22]	Fructus Aurantii Decoction	Citrus aurantium L. immature fruit (Zhike)	Immature fruit	Water decoction	Crude material soaked for 30 min and decocted twice for 30 min each; concentrated to 0.416 g crude drug/mL stock solution	Naringin and Neohesperidin	Naringin 3.11%; Neohesperidin 3.68% in decoction	Yes	HPLC; structural identification by ^1H-NMR, ^13C-NMR and ESI-MS for standards	Authenticated by Associate Professor Lai Xuewen, Jiangxi University of TCM	Not reported	NR

Supplementary Table 9. Characteristics, extraction methods, and phytochemical characterization of single herb extracts

Study (Year)	Intervention name	Botanical source	Plant part used	Extraction method	Extraction details	Major chemical constituents reported	Percentage/ concentration reported	Chemical characterization performed	Analytical method	Botanical authentication method	Storage/ stability reported	Storage details
Niijima 1998[23]	*Pinellia*, *Zingiberis*	*Pinellia ternata*; *Zingiber officinale* Roscoe (*Zingiberis rhizoma*)	Tuber (*Pinellia*); rhizome (*Zingiber*)	Water extraction under reflux	Commercial tubers of *Pinellia ternata* and rhizomes of *Zingiberis* were pulverized, extracted with water under reflux, filtered, and lyophilized. Extracts were stored in a desiccator before use. For administration, 50 mg extract was dissolved in 1 mL distilled water and homogenized using an ultrasonic cleaner before oral application to the tongue for 10 min.	NR	50 mg/mL extract concentration; mixture ratio 5:1 (*Pinellia*:*Zingiberis*)	No	NR	NR	Yes	Extracts stored in desiccator prior to use
Lu 1984[24]	Raw or wine-stewed *Rheum palmatum*	*Rheum palmatum L. (Qinghai rhubarb)*	Rhizome and root (peeled)	Suspension in distilled water; rice wine processing for processed product	Raw rhubarb powder was sieved (60 mesh) and suspended in distilled water at 0.15 g/mL. For wine-processed rhubarb, the herb was stewed with Shaoxing rice wine, the wine solution evaporated to dryness, and the residue resuspended in distilled water to the same concentration. Dose: 0.075 g/100 g body weight per administration.	NR	Suspension concentration 0.15 g/mL; dose 0.075 g/100 g body weight	No	NR	NR	No	NR
Luo 1996[25]	*Medicago sativa* extract	*Medicago sativa L. (alfalfa)*	NR	Water extraction (presumed from "aqueous solution")	Animals received 0.05% alfalfa extract aqueous solution via lingual vein injection or intracerebroventricular injection. No extraction procedures or preparation details were reported.	NR	0.05% aqueous extract solution	No	NR	NR	No	NR
Fu 2013[26]	Aloe extract	*Aloe spp.*	Leaf (presumed gel extract; exact plant part not reported)	NR	Aloe extract containing crude drug equivalent of 0.165 g/mL was administered by gavage at 12 mL/kg twice daily for 7 days. No extraction procedures, solvent details, or yield information were reported.	NR	0.165 g crude drug/mL; 12 mL/kg twice daily	No	NR	NR	No	NR
Zhao 2010[27]	*Alpinia officinarum* extract	*Alpinia officinarum Hance*	Rhizome	Water extraction and ethanol extraction	Three doses of water extract and ethanol extract were administered orally: 6.66, 3.33, and 1.67 mg/kg (crude drug equivalent) once daily	NR	6.66, 3.33, and 1.67 mg/kg (crude drug equivalent)	No	NR	NR	No	NR

					for 10 days. Extraction procedures, solvent concentrations, extraction yields, and preparation protocols were not described.						
Li 2023[28]	Galangal + ginger extracts	*Alpinia officinarum Hance and Zingiber officinale Roscoe*	Rhizomes	Water decoction	Dried ginger decoction prepared using a 1:10 herb-to-water ratio, soaked for 30 min and decocted twice for 30 min each. The combined filtrates were concentrated to 0.5 g/mL crude drug concentration. Galangal decoction was prepared identically and concentrated to 0.27, 0.54, and 1.08 g/mL. Combination extracts were prepared by mixing ginger decoction with galangal decoctions of varying concentrations.	Ginger volatile oils, gingerols, and diarylheptanoids mentioned in introduction; no constituent analysis performed on study extracts	Dry ginger: 0.5 g/mL; galangal: 0.27, 0.54, and 1.08 g/mL	No	NR	Galangal authenticated by Professor Zhou Zhongliu, Lingnan Normal University; origin from Xuwen County, Guangdong	Expert botanical identification	Yes
El-Akabawy 2014[29]	Ginger extract	*Zingiber officinale Roscoe*	NR	NR	Rats received ginger by oral gavage at 500 mg/kg/day throughout the study period (4, 6, or 8 weeks). The source of ginger, extraction solvent, preparation method, and formulation details were not reported.	Gingerols, shogaols, and phenolic ketone derivatives mentioned as known ginger antioxidants, but not quantified in study material	500 mg/kg/day	No	NR	NR	No	NR
Zhong 2022[30]	Ginger extract	*Zingiber officinale Roscoe*	Rhizome	Ultrasonic ethanol extraction	Fresh ginger was washed, sliced, dried, and powdered. Ginger powder (20 g) was ultrasonically extracted with 300 mL of 80% ethanol. The extract was filtered, concentrated using a rotary evaporator at 40°C until ethanol removal, centrifuged (3000 g, 10 min), and the supernatant was freeze-dried.	Gingerol, ginger oil, and terpenoids identified as major active components	NR	Yes	LC-MS/MS	NR	No	NR
Kim 2023[31]	Ginger extract	*Zingiber officinale Roscoe*	Rhizome	Maceration extraction with ethanol	Ginger (600 g) was extracted with 1 L of 80% ethanol at room temperature for 72 h, filtered, concentrated using rotary evaporation at 60°C, and freeze-dried overnight. Final dried extract yield was 22.42 g (5.6%). Extract powder was dissolved in distilled water before oral administ	[6]-Gingerol and [6]-shogaol	[6]-Gingerol 4.12%; [6]-shogaol 2.15%; extraction yield 5.6%	Yes	HPLC	Voucher specimen deposited (KWJ-0001)	Voucher specimen at Kyung Hee University	No

					ration.							
Zhao 2024[32]	*Monochasma savatieri* extract	*Monochasma savatieri Franch.*	Whole plant	Water decoction	Whole plant material (500 g) was soaked in eight volumes of water for 1 h and decocted for 1.5 h. The residue was then extracted again with six volumes of water for 40 min. Combined extracts were filtered and concentrated under reduced pressure to obtain dry extract. Extraction yield was 14.4%. Final extract stored at −20°C.	Luteolin, isoacteoside, aucubin identified as major active ingredients	Extraction yield 14.4%; extract concentration for LC-MS analysis 30 mg/mL	Yes	UPLC-QTOF-MS/MS	Identified by Professor Shuyuan Li; voucher specimen no. 20180928 deposited in herbarium	Expert authentication with voucher deposition	Yes
Shih 2008[33]	*Rhodiola sacra* water fraction	*Rhodiola sacra S. H. Fu*	Radix (root)	Methanol extraction followed by solvent partitioning	Air-dried chopped radix (150 g) extracted with methanol (1 L) at 55°C for 7 h ×3. Crude methanol extract was suspended in water and sequentially partitioned with n-hexane, dichloromethane, ethyl acetate, and n-butanol to obtain fractions. Yields: hexane 1.28 g, dichloromethane 0.94 g, ethyl acetate 1.11 g, BtF 0.8 g, WtF 16.5 g.	Phenolic glycosides, cyanogenic glycosides, flavonoids, terpenoids, organic acids reported from the species	Fraction yields reported (WtF 16.5 g; BtF 0.8 g)	No	NR	Authenticated by Dr. Eric I-Chian Li; voucher specimen deposited (No. GiuDing 94001)	Yes	Fractions stored in capped containers at 4°C
Cui 2012[34]	*Scutellaria baicalensis* extract	*Scutellaria baicalensis Georgi*	Dried root	Traditional aqueous extraction	Dried roots were decocted twice with 10 volumes of distilled water for 1 h each. Combined decoctions were concentrated under reduced pressure to a crude drug concentration of 1 g/mL and diluted to required concentrations for administration.	Baicalin used as quality marker	Baicalin content ≥9% according to Chinese Pharmacopoeia 2010	Yes	HPLC	Authenticated by Prof. Zhang Yi, Chengdu University of Traditional Chinese Medicine	No	NR
Medeiros 2006[35]	*Sida cordifolia* fraction	*Sida cordifolia L.*	Leaves	Hydroalcoholic extraction and aqueous partitioning	Leaves were dried at 40°C and extracted with 70% ethanol for 72 h at room temperature. Extract was dried at 60°C and a portion dissolved in distilled water, filtered, and dried to obtain the water-soluble fraction (AFSC). Prior to use, AFSC was dissolved in saline containing cremophor (0.025% v/v).	Alkaloids, steroids, flavonoids, saponins; ephedrine, pseudoephedrine, vasicinone, vasicine, vasicinol reported from leaves	NR	No	Preliminary phytochemical screening only	Voucher specimen deposited (No. 30171) at University of Sergipe	No	NR
Wang 2021[36]	*Drynaria fortunei*	*Zingiber officinale Roscoe*	Rhizome	95% ethanol extraction followed by chromatographic fractionation	Fresh rhizomes were cleaned, sliced, air-dried, powdered, and extracted with 95% ethanol for 72 h. Extract concentrated under vacuum at 45°C using rotary evaporation. Approximately 50 g extra	14 bioactive compounds identified including 6-gingerol, sinapinic acid, p-coumaric acid,	6-Gingerol constituted 51.05% of the fraction	Yes	HPLC with diode-array detection; TLC screening	Authenticated by Plant Biology Department, University of Ilorin; voucher sp	No	NR

					ct dissolved in 100 mL ethyl acetate and subjected to si lica gel column chromatogra phy (70–230 mesh), defatted with n-hexane, and eluted with n-hexane:ethyl acetate (1:1, 500 mL). Ten fractions obtained and analyzed by TL C.	benzoic acid, α-farnesene , caffeic acid , zingiberene , gingerone A, cinnamic acid, apigeni n, syringic a cid, and fer ulic acid				ecimen UIL H/001/116 8/2022		
Asuku 20 24[37]	6-gingerol-rich *Zingiber officin ale*	*Drynaria fortunei (Kunze) J.Sm.*	Rhizome	Water extr action (refl ux decocti on)	100 g dried rhizome extract ed under reflux with 10 vol umes of water for 1.5 h, fil tered, then re-extracted with 8 volumes of water for 1 h. Combined extracts were concentrated to 1 g crude d rug/mL.	NR	Final concentr ation 1 g cru de drug/mL; administered at 0.975 g/kg /day	No	NR	Authenticat ed by Asso ciate Profe ssor Chen Xiaozhong, Jiamusi Col lege, Heilo ngjiang Uni versity of Chinese M edicine	No	NR

Supplementary Table 10. Characteristics, extraction methods, and phytochemical characterization of phytoconstituents

Study (Year)	Intervention name	Botanical source	Plant part used	Extraction method	Extraction details	Major chemical constituents reported	Percentage/ concentration reported	Chemical characterization performed	Analytical method	Botanical authentication method	Storage/ stability reported	Storage details
Wang 2022[38]	L-Borneolum, Natural Borneolum, Borneolum Syntheticum	*Blumea balsamifera* (L.) DC. (natural borneol source)	NR	NR	Oral administration for 3 days before LAD ligation and 3 days after modeling. Natural borneol doses: 0.15, 0.30, 0.60 g/kg. L-borneol and synthetic borneol doses: 0.05, 0.10, 0.20 g/kg.	NR	Dose ranges reported only	No	NR	NR	No	NR
Zaki 2020[39]	Curcumin and Nano-curcumin	*Curcuma longa* L.	Rhizome	Commercial curcumin preparation; nanoformulation prepared using sonication method	Curcumin purchased from Sigma-Aldrich and dissolved in 5% dextrose. Nano-curcumin prepared by dissolving 0.018 M curcumin in dichloromethane followed by dropwise addition into warm ultrapure water under ultrasonic sonication, centrifugation, autoclaving, freeze-drying, and lyophilization.	Curcumin	0.018 M for nanoparticle synthesis; treatment dose 2.5 mg/kg BW	No	NR	NR	Yes	Freeze-dried and lyophilized to obtain dry nano-curcumin in powder
Ren 2012[40]	Curcumin	*Curcuma longa* L.	Rhizome	NR	APP/PS1 mice received daily intragastric administration of curcumin for 3 months. Exact preparation procedures and formulation details were not reported.	Curcumin	NR	No	NR	NR	No	NR
Zhang 2012[41]	Curcumin	*Curcuma longa* L.	Rhizome	Extraction method not reported (commercial curcumin preparation)	Curcumin administered to AD rats at low, medium, and high doses; formulation details and preparation procedures were not described.	Curcumin	NR	No	NR	NR	No	NR
Wang 2009[42]	Curcumin plus indomethacin	*Curcuma* spp.	Rhizome (tuber state d in manuscript)	Commercial preparation dissolved in vehicle	Curcumin supplied by Hebei Hualong Co., Ltd. Curcumin was dissolved in distilled water containing 1% sodium carboxymethylcellulose and administered by gavage at 100 mg/kg/day for 14 days.	Curcumin	100 mg/kg/day	No	NR	NR	No	NR
Abu-Taweel 2016[43]	Curcumin	*Curcuma longa L.*	Rhizome	Commercial analytical-grade compound	Curcumin (Sigma Chemical Co., USA) dissolved in 50% DMSO and administered orally once daily for 2 weeks at doses of 150 or 300 mg/kg.	Curcumin (commercial product reported to contain curcuminoids)	150 and 300 mg/kg BW; commercial curcumin composition reported in introduction as 77% curcumin, 17% demethoxycurcumin, and 3%	No	NR	NR	No	NR

							bisdemethoxycurcumin.					
Xu 2018[44]	Curcumin + vagus nerve stimulation	*Curcuma longa L.*	Rhizome	Commercial preparation	Curcumin purchased from Sigma-Aldrich and dissolved in normal saline containing 1% DMSO. Rats received intraperitoneal injection of curcumin at 50 mg/kg daily for five days before MCAO surgery.	Curcumin	50 mg/kg IP for 5 consecutive days	No	NR	NR	No	NR
Mulla 2025[45]	Dietary curcumin	*Curcuma longa L.*	Rhizome	Incorporated directly into formulated diets	Three experimental diets were prepared containing 0%, 0.1%, and 0.2% curcumin and administered to fish under chromium and ammonia stress conditions.	Curcumin (diferuloylmethane)	0.1% and 0.2% dietary supplementation	No	NR	NR	No	NR
Dou 2018[46]	Curcumin	*Curcuma longa L.*	Rhizome	Commercial preparation suspended in vehicle	Curcumin (Nanjing Zelang Medical Technology, China) was suspended in 0.5% sodium carboxymethylcellulose and orally administered at 100 mg/kg daily for 2 weeks.	Curcumin	100 mg/kg/day orally	No	NR	NR	No	NR
Lim 2023[47]	Curcumin	*Curcuma longa L.*	Rhizome	Commercial curcumin preparation	Curcumin powder containing 65% curcumin was purchased from Sigma-Aldrich and dissolved in 10% Tween 80 before administration. Curcumin was administered orally at 100 or 200 mg/kg/day.	Curcumin	65% curcumin preparation; doses of 100 and 200 mg/kg/day	Partial	No analytical technique reported	NR	No	NR
Öz 2024[48]	Curcumin	*Curcuma longa L.*	Rhizome	Commercial curcumin preparation	Curcumin was administered orally as a positive control at 50 mg/kg during the final two days of the experiment in an LPS-induced neuroinflammation model.	Curcumin	50 mg/kg orally	No	NR	NR	No	NR
Yang 2023[49]	Curcumin (CUR)	*Curcuma longa L.*	Rhizome	Commercial preparation dissolved in DMSO	CUR stock solution (10 mg/mL) was prepared in DMSO and stored at 4°C prior to use in zebrafish embryo exposure experiments. CUR was purchased from Yuan Ye Biotechnology Co., Ltd., China.	Curcumin	Stock concentration 10 mg/mL	No	NR	NR	Yes	Stock solutions stored at 4°C
Wu 2017[50]	Curcumin	*Curcuma longa L.*	Rhizome	Commercial curcumin preparation	Curcumin was described as the active compound derived from Curcuma longa and administered to aged mice with surgery-induced cognitive dysfunction.	Curcumin	Dose not reported in available text snippet	No	NR	NR	No	NR
Fikry 2021[51]	Curcumin	*Curcuma longa L.*	Rhizome	Commercial reagent	Curcumin (Sigma-Aldrich; CAS 458-37-7) was dissolved in DMSO and administered intraperitoneally at 30 mg/kg/d	Curcumin (diferuloylmethane)	30 mg/kg/day i.p.	Partial	CAS number reported, but no chrom	NR	No	NR

					ay for 60 days in rotenone-treated rats.				atography			
Keshk 2020[52]	Curcumin	*Curcuma longa L.*	Rhizome	Commercial powder	Curcumin powder (purity ≥65%, CAS# C1386) purchased from Sigma and administered orally at 100 mg/kg/day in 1% Tween 80 for eight weeks.	Curcumin	Purity ≥65%; dose 100 mg/kg/day	Partial	No analytical technique reported	NR	No	NR
Lan 2018[53]	Curcumin	*Curcuma longa L.*	Rhizome	Commercial preparation	Curcumin was administered daily to stroke-prone spontaneously hypertensive rats at 100 mg/kg/day for stroke prevention experiments.	Curcumin	100 mg/kg/day	No	NR	NR	No	NR
Abdel-Diam 2018[54]	Curcumin (CUR)	*Curcuma longa L.*	Rhizome	Commercial preparation	Administered orally by gavage for 28 days in DZN-exposed rats. Dose not specified in available text snippet.	Curcumin	NR	No	NR	NR	No	NR
Song 2016[55]	Curcumin (CM)	*Zingiberaceae source reported; consistent with Curcuma longa L.*	Rhizome	Commercial preparation dissolved in DMSO and peanut oil	Curcumin purchased from Sigma-Aldrich; purity ≥94%; dissolved in DMSO then mixed with peanut oil containing final 0.05% DMSO concentration; oral administration 5, 10, or 20 mg/kg/day for 30 days.	Curcumin	Purity ≥94%	Partial	Purity reported only; no chromatography	NR	No	NR
Akintunde 2019[56]	Turmeric curcumin (CUR)	*Curcuma longa L.*	Rhizome	Commercial reagent	Curcumin procured from Sigma-Aldrich and administered orally at 50 or 100 mg/kg/day for 14 days in BPA-treated rats.	Curcumin	50 or 100 mg/kg/day	No	NR	NR	No	NR
Blackshaw 2000[57]	Capsaicin	*Capsicum spp.*	Fruit	Commercial capsaicin reagent	Topical or systemic capsaicin applied to gastrointestinal vagal afferents in ferrets to investigate acute vagal activation and desensitization.	Capsaicin	1–3.25 mM topical or 32–164 nmol systemic doses	No	NR	NR	No	NR
Jancsó 1983[58]	Capsaicin	*Capsicum spp.*	Fruit	Capsaicin dissolved in saline with ethanol and Tween 80	Capsaicin applied perineurally to cervical vagus nerves to study cardiovascular and respiratory reflexes in cats.	Capsaicin	NR	No	NR	NR	No	NR
Mei 2013[59]	Puerarin	*Pueraria lobata (Willd.) Ohwi*	Root	Injectable pharmaceutical preparation	Puerarin injection supplied by Ruiyang Pharmaceutical; intravenous administration 18 mg/kg for 5 days prior to MCAO induction.	Puerarin	18 mg/kg	No	NR	NR	No	NR
Wang 2012[60]	Puerarin	*Pueraria lobata (Willd.) Ohwi*	Root	Injectable pharmaceutical preparation	Puerarin administered intravenously for 5 days before cerebral ischemia-reperfusion induction.	Puerarin	NR	No	NR	NR	No	NR
Kaboutari 2024[61]	Menthol	*Mentha canadensis / Mentha × piperita source mentioned*	NR	Commercial compound	Menthol purchased from Sigma-Aldrich; dissolved in DMSO; administered IP at 10 mg/kg daily for 28 days	Menthol	10 mg/kg/day	No	NR	NR	No	NR

Dou 2019[62]	Madecassoside	*Centella asiatica*	NR	Commercial purified compound	Madecassoside purchased from Nanjing Zelang Medical Technology; orally administered at 30 mg/kg for 2 weeks	Madecassoside	Purity >98%; dose 30 mg/kg	Yes / partial	HPLC purity confirmation	NR	No	NR
Lu 2018[63]	3-Dehydroandrographolide	*Andrographis paniculata*	NR	Isolated compound	3-DA isolated previously; used in LPS-induced inflammation models	3-Dehydroandrographolide	Purity >99%	Yes	HPLC	NR	No	NR
Ai 2013[64]	Angelica sinensis polysaccharides (ASP)	*Angelica sinensis (Oliv.) Diels*	Root	Hot-water extraction	Dried roots cut into slices; optimal extraction: 180 min, water:solid ratio 6, 100°C, extracted 4 times	Polysaccharides	Extraction yield 5.6%; doses 150 or 300 mg/kg	Yes	Chemical characterization reported in title; exact method not fully visible in snippet	NR	No	NR
Ding 2018[65]	Breviscapine injection	*Erigeron breviscapus*	NR	Commercial injection	Breviscapine injection purchased from Jilin Longtai Pharmaceutical; administered IP at 25 or 50 mg/kg/day for 1 week before MIRI	Breviscapine	25 or 50 mg/kg/day	No	NR	NR	No	NR
Bai 2020[66]	Trans-anethole (ANE)	*Anise/fennel source mentioned generally*	NR	NR	ANE administered by gavage at 100 mg/kg/day for 4 weeks in COPD rats	Trans-anethole	100 mg/kg/day	No	NR	NR	No	NR

Supplementary Table 11. Characteristics, extraction methods, and phytochemical characterization of phytoconstituent derivatives and formulations

Study (Year)	Intervention name	Botanical source	Plant part used	Extraction method	Extraction details	Major chemical constituents reported	Percentage/ concentration reported	Chemical characterization performed	Analytical method	Botanical authentication method	Storage/ stability reported	Storage details
Khadrawy 2021[67]	Nanocurcumin	*Curcuma longa* L.	Rhizome	Commercial nanoformulation	Nanocurcumin administered orally at 50 mg/kg prior to doxorubicin administration	Curcumin	50 mg/kg	No	NR	NR	No	NR
Ma 2024[68]	COP-22	Synthetic curcumin derivative	NA	Chemical synthesis	COP-22 synthesized according to previous publication and dissolved in physiological saline for oral administration for 10 weeks	COP-22	50, 100, and 200 mg/kg	No	NR	NR	No	NR
Noor 2022[69]	Curcumin nanoparticles	*Curcuma longa* L.	Rhizome	Commercial nanoformulation	Nanocurcumin purchased from One Planet Nutrition Company and administered orally at 50 mg/kg before and after STZ injection	Curcumin nanoparticles	50 mg/kg	Partial	Chemical composition described only	No	NR	No
Shabbir 2022[70]	Curcuminoid-rich extract and ternary complex	*Curcuma longa* L.	Rhizome	Ethanol extraction using microwave-assisted extraction	Turmeric extracted with ethanol using MAE, semipurified using Diaion HP-20 resin and hydroethanol elution to produce CRE; TC prepared using hydroxypropyl-β-cyclodextrin and polyvinylpyrrolidone K30	Curcuminoids (curcumin, demethoxycurcumin, bisdemethoxycurcumin)	CRE: 88% w/w curcuminoids; TC: 14% w/w curcuminoids; crude extract: 27% w/w curcuminoids	Yes	HPLC	NR	No	NR
Guo 2018[71]	Solid dispersions of bisdemethoxycurcumin, curcumin, demethoxycurcumin, and tetrahydrocurcumin	*Curcuma longa* L.	Rhizome	Solid dispersion preparation	Solid dispersions prepared according to patented methods using polyethylene glycol-based carriers; administered by gavage for 35 days	Bisdemethoxycurcumin, Curcumin, Demethoxycurcumin, Tetrahydrocurcumin	1 g dispersion equivalent to 0.077 g active ingredient; dose 200 mg/kg	No	NR	NR	Yes	Stored protected from light in cool conditions
Mohammed 2020[72]	Curcumin nanoparticles	*Curcuma longa* L.	Rhizome	Commercial nanoformulation	CUR-NPs purchased from One Planet Nutrition Company and administered orally at 50 mg/kg for 14 days	Curcumin nanoparticles	50 mg/kg	Yes	TEM imaging and UV-Vis spectrophotometry	NR	No	NR

Supplementary Table 12. Characteristics, extraction methods, and phytochemical characterization of essential oils

Study (Year)	Intervention name	Botanical source	Plant part used	Extraction method	Extraction details	Major chemical constituents reported	Percentage/ concentration reported	Chemical characterization performed	Analytical method	Botanical authentication method	Storage/ stability reported?	Storage details
Zhang 2008[73]	Essential oil from *Atractylodes lancea*	*Atractylodes lancea* (Thunb.) De Candolle	Rhizome	Hydrodistillation	Dried rhizomes were cut into small segments. One kilogram of rhizomes was immersed in 8 L distilled water and subjected to hydrodistillation for 8 hours. Essential oil yield was 7.6% (v/w). The oil was dissolved in 3% Tween-80 before administration.	β-eudesmol, atractylodin, β-selinene, α-phellandrene, hinesol	β-eudesmol 34.15%; atractylodin 17.19%; β-selinene 7.59%; α-phellandrene 6.42%; hinesol 4.32%	Yes	Gas chromatography–mass spectrometry	Identified by Professor Han-Chen Zheng, School of Pharmacy, Second Military Medical University	Yes	Stored at 4°C until use
Satou 2022[74]	Essential oil from *Pelargonium graveolens* (Geranium essential oil)	*Pelargonium graveolens* L'Hér.	Aerial parts (not specifically reported)	Commercial essential oil preparation	Essential oil was purchased commercially and administered by inhalation at 5 µL/L air for 90 minutes. No extraction procedure was described because the study used a commercial product.	Citronellol, geraniol, citronellyl formate, linalool	Citronellol 21.9%; geraniol 13.3%; citronellyl formate 8.6%; linalool 6.3%	Yes	Gas chromatography–mass spectrometry	Not reported	No	NR
Komiya 2009[75]	Lavender oil	*Lavandula angustifolia* Miller	Flowering tops (not specifically reported in manuscript)	Commercial essential oil preparation	Undiluted lavender oil (0.18 mL) was topically applied to the inner surface of both ears four times daily. The oil was commercially obtained and analyzed independently before use.	Chemical composition determined but not fully reported in manuscript body; detailed composition provided in appendix	Not reported in main manuscript	Yes	Gas chromatography–mass spectrometry	Not reported	No	NR

References

1. Wang XZ, Jiang JY, Luo SS. [Effect of banxia baizhu tianma decoction on the vascular endothelial function of spontaneous hypertensive rats]. Zhongguo Zhong Xi Yi Jie He Za Zhi 2011;31(6):811–5.

2. Lu Y, Chen Y, Wang YN, Liu H, Zhang JS, Ma WG, et al. [Effect of Banxia Qinlian Decoction on Th17/IL-17 Immune Inflammatory Way of Sjögren's Syndrome NOD Model Mice]. Zhongguo Zhong Xi Yi Jie He Za Zhi 2015;35(5):612–7.

3. Zhang Z, Si Y, Wu H, Xu H, Bai L. Effects of Banxia Xiexin Decoction and its component prescriptions on cholinergic neurons in rats with stress-induced gastric ulcers. Chin J Basic Med Tradit Chin Med 2005(04):283–84+87.

4. Du YP, Zhang YP, Zhang GC. [Effects of Baobaole oral liquid on neuron excitability of feeding center in anorectic rats]. Zhong Xi Yi Jie He Xue Bao 2007;5(5):550–4.

5. Xie MZ, Qi QH, Zhang SL, Wei MM. Effects of Dachengqi Decoction on morphological changes in enteric nerve system of rats with multiple organ dysfunction syndrome. Chin J Integr Med 2015;21(8):624–9.

6. Zhang YP, Du YP, Zhang GC. [Effect of er'bao granule on integration of ingestion behavior-related information by neurons in lateral hypothalamic area of anorexia rats]. Zhongguo Zhong Xi Yi Jie He Za Zhi 2005;25(11):996–9.

7. Jiang P, Ma DF, Jiang YH, Yang JL, Xu XD, Wang X, et al. [Preventive Effect of Different Compatibilities of Ramulus Cinnamomi and Radix Paeomlae alba in Guizhi Decoction on Cardiac Sympathetic Denervation Induced by 6-OHDA]. Zhongguo Zhong Xi Yi Jie He Za Zhi 2016;36(5):608–13.

8. Li X, Jiang YH, Jiang P, Yang JL, Ma DF, Yang CH. Effect of Guizhi Decoction on heart rate variability and regulation of cardiac autonomic nervous imbalance in diabetes mellitus rats. Chin J Integr Med 2014;20(7):524–33.

9. Li X, Yang JL, Ma DF, Lin HQ, Xu XD, Jiang Yue H. [Effects of Different Compatibilities of Ramulus Cinnamomi and Peony in Guizhi Decoction on Diabetic Cardiac Autonomic Neuropathy]. Zhongguo Zhong Xi Yi Jie He Za Zhi 2015;35(6):741–5.

10. Sun YB, Ni Y, Fan XS, Zhou LP, Yue QF, Shang EX. Effect of Houpo-Mahuang Decoction on aggravated asthma induced by cigarette smoke and the expression of TRPA1 and tight junctions in mice. J Ethnopharmacol 2022;293:115217.

11. Sun Y, Zhou D, Liu A, Zhou Y, Zhao Y, Yuan Y, et al. Liangxue Tongyu Prescription exerts neuroprotection by regulating the microbiota-gut-brain axis of rats with acute intracerebral hemorrhage. Brain Res Bull 2025;220:111186.

12. Zhang WW, Sun QX, Liu YH, Gao W, Li YH, Lu K, et al. Chronic administration of Liu Wei Dihuang

protects rat's brain against D-galactose-induced impairment of cholinergic system. Sheng Li Xue Bao 2011;63(3):245–55.
13. Bai J, Gao Y, Gao Y-H. Effect of Pei Yuan Tong Nao capsules on neuronal function and metabolism in cerebral ischemic rats. Journal of Ethnopharmacology 2019;238:111837.
14. Nakyam T, Wattanathorn J, Thukham-Mee W, Muchimapura S. The Polyherbal Functional Ingredient Containing Ginger, Chinese Date, and Wood Ear Mushroom Protects against Dementia following Metabolic Syndrome. Biomed Res Int 2023;2023:9911397.
15. Jiang CX, Fan XS, Gu PC, Xu HQ. The effect of Qi'ao Decoction on ovalbumin induced and lipopolysaccharide enhanced severe asthma mice and its mechanism. Chin J Nat Med 2013;11(6):638–44.
16. Gan y, Ma Y-h, Bao Y-l, Zhang H, Qiao M, Meng L, et al. [Effects of Shegan Zhike capsult on relieving post-infectious cough and inhibiting arachidonic acid metabolim pathway]. Lishizhen Med Mater Med Res 2023;34(04):828–31.
17. Wu Q, Cao Y, Liu M, Liu F, Brantner AH, Yang Y, et al. Traditional Chinese Medicine Shenmayizhi Decoction Ameliorates Memory And Cognitive Impairment Induced By Scopolamine Via Preventing Hippocampal Cholinergic Dysfunction In Rats. Neuropsychiatr Dis Treat 2019;15:3167–76.
18. Wu Q, Liu Y, Gao J, Shao C, Shen Z, Chen T, et al. Influence of Sini Decoction on blood pressure and baroreflex sensitivity in rats with abdominal aortic coarctation. Chin J Integr Med Cardio Cerebrovasc Dis 2015;13(12):1381–84.
19. Qi J. Effect of Wenyang Tongzhi capsule on rats chronic-moving arrhythmia caused by CLO. Chin Med Mod Distance Educ China 2014;12(16):157–59.
20. Ma J, Zhang Y. Effect of Supplementing Qi and activating blood circulation combined with exercise training on ventricular autonomic nerve remodeling in heart failure rats. J Basic Chin Med 2016;22(05):617–20.
21. Du Y-P, Wang X, Zhang Y-P, Niu Y. Yunpi prescription reduces excitability of VMN neurons in anorexic rats. J Fourth Mil Med Univ 2006;27(6):540–43.
22. Tan S, Chen H, Song Y, Hu Y, Luo X, Yang W. Effects of Fructus Aurantii (Zhike) and its main active components on serum gastrin, plasma acetylcholine, motilin, substance P, and vasoactive intestinal peptide in spleen-deficiency model rats. Lishizhen Med Mater Medica Res 2017;28(05):1037–40.
23. Niijima A, Kubo M, Hashimoto K, Komatsu Y, Maruno M, Okada M. Effect of oral administration of Pinellia ternata, Zingiberis rhizoma and their mixture on the efferent activity of the gastric branch of the vagus nerve in the rat. Neuroscience Letters 1998;258(1):5–8.
24. Lu Z, Sun S, Dong X, Ma C, Jiang W, Yu M, et al. The effect of Rheum on the level of plasma cAMP and cGMP in rats with stress gastric ulcer. J Tradit Chin Med West Med Integr 1984(10):622–23+581.

25. Luo J, Wen C, Du H. Effects of Alfalfa extract on cervical vagus nerve discharge of rats. J Jilin Med Coll 1996(01):19–20.
26. Fu L-x, Tang D-m, Yan H-x. The effect of vagus on the protection of stress ulcer by Aloes. J Southwest Univ Natl (Nat Sci Ed) 2013;39(02):171–75.
27. Zhao Y, Liu X, Chen C, Bai J. Effects of extract of Alpinia officinarum on learning and memory deficits and the function of the central cholinergic system in mice. Pharmacol Clin Chin Mater Med 2010;26(02):49–52.
28. Li Z-sL, Cheng-long, Wen Z-r, Ou J-x, Lai J, Huang L-p. Mechanism of anti-gastric cold effect of galangal-dried ginger extract on gastric cold model mice. J Guangdong Med Univ 2023;41(06):606–12.
29. El-Akabawy G, El-Kholy W. Neuroprotective effect of ginger in the brain of streptozotocin-induced diabetic rats. Ann Anat 2014;196(2-3):119–28.
30. Zhong W, Zhu J, Yi J, Zhao C, Shi Y, Kang Q, et al. Biochemical analysis reveals the systematic response of motion sickness mice to ginger (Zingiber officinale) extract's amelioration effect. Journal of Ethnopharmacology 2022;290:115077.
31. Kim H, Park KT, Jo H, Shin Y, Chung G, Ko SG, et al. The effect of ginger extract on cisplatin-induced acute anorexia in rats. Front Pharmacol 2023;14:1267254.
32. Zhao M, Yang M, Du J, Cao X, Zhong L, Li W, et al. Monochasma savatieri Franch. protects against acute lung injury via α7nAChR-TLR4/NF-κB p65 signaling pathway based on integrated pharmacology analysis. Journal of Ethnopharmacology 2024;321:117487.
33. Shih C-D, Kuo D-H, Huang C-W, Gu Y-H, Chen F-A. Autonomic nervous system mediates the cardiovascular effects of Rhodiola sacra radix in rats. Journal of Ethnopharmacology 2008;119(2):284–90.
34. Cui R, Meng X-l, Wang P, Li H, Yu Y-p. Correlation between protection of Scutellaria baicalensis extract on rats' acute lung injury and cholinergic anti-inflammatory pathway. Chin Tradit Herb Drugs 2012;43(02):321–26.
35. Medeiros IA, Santos MRV, Nascimento NMS, Duarte JC. Cardiovascular effects of Sida cordifolia leaves extract in rats. Fitoterapia 2006;77(1):19–27.
36. Wang D, Chen X, Su T, Xing S-r, Zhang N, Ding L-x, et al. Effects of Drynaria fortunei on Learning and Memory Ability of Scopolamine Model Mice. Chin Med J Res Prac 2021;35(05):15–20.
37. Asuku AO, Ayinla MT, Ajibare AJ, Olajide TS. Mercury chloride causes cognitive impairment, oxidative stress and neuroinflammation in male Wistar rats: The potential protective effect of 6-gingerol-rich fraction of Zingiber officinale via regulation of antioxidant defence system and reversal of pro-inflammatory markers increase. Brain Res 2024;1826:148741.
38. Wang L, Wang J, Fan Y, Wang J, Fu Y, Yang X, et al. Anti-inflammation and Regulation of HIF-

1α/VEGF of 3 Kinds of Borneolum in Prevention and Treatment of AMI Model Rats. Chin J Exp Trad Med Formulae 2022;28(19):61–72.
39. Zaki SM, Algaleel WAA, Imam RA, Soliman GF, Ghoneim FM. Nano-curcumin versus curcumin in amelioration of deltamethrin-induced hippocampal damage. Histochem Cell Biol 2020;154(2):157–75.
40. Ren Y, Li R-s, Wang H, Sun H-y, Yang J-d, Wang P-w. Influence of curcumin on hippocampus neuron ChAT in APP /PS1 double transgenic mice. J Beijing Univ Tradit Chin Med 2012;35(10):679–82+722.
41. Zhang X, Zhao Z, Zhang C. Relationship between Amelioration of Curcumin on Learning and Memory Ability and the Contents of NO、SOD、MDA and ChAT in Brain of AD Rats. J Med Res 2012;41(09):82–86.
42. Wang C, Hu W, Yang B, Li G, Pei Y. Effects of combined curcumin and indomethacin on hippocampal ChAT and AChE activity in an Alzheimer's disease rat model. China Mod Doctor 2009;47(32):21–22+45.
43. Abu-Taweel GM. Effects of curcumin on the social behavior, blood composition, reproductive hormones in plasma and brain acetylcholinesterase in cadmium intoxicated mice. Saudi J Biol Sci 2016;23(2):219–28.
44. Xu J, Kong X, Xiu H, Dou Y, Wu Z, Sun P. Combination of curcumin and vagus nerve stimulation attenuates cerebral ischemia/reperfusion injury-induced behavioral deficits. Biomed Pharmacother 2018;103:614–20.
45. Mulla AN, Thorat ST, Chandramore K, Kumar P, Reddy KS, Kumar N. Curcumin as a protective agent against chromium and ammonia toxicity using molecular and biochemical approaches in fish. Sci Rep 2025;15(1):12023.
46. Dou Y, Luo J, Wu X, Wei Z, Tong B, Yu J, et al. Curcumin attenuates collagen-induced inflammatory response through the "gut-brain axis". J Neuroinflammation 2018;15(1):6.
47. Lim JY, Kim W, Ha AW. The effect of curcumin on blood pressure and cognitive impairment in spontaneously hypertensive rats. Nutr Res Pract 2023;17(2):192–205.
48. Öz M, Erdal H. A TNF-α inhibitor abolishes sepsis-induced cognitive impairment in mice by modulating acetylcholine and nitric oxide homeostasis, BDNF release, and neuroinflammation. Behav Brain Res 2024;466:114995.
49. Yang Q, Liu J, Ding J, Liu J. Neurodevelopmental toxicity of bisphenol AF in zebrafish larvae and the protective effects of curcumin. J Appl Toxicol 2023;43(12):1806–18.
50. Wu X, Chen H, Huang C, Gu X, Wang J, Xu D, et al. Curcumin attenuates surgery-induced cognitive dysfunction in aged mice. Metab Brain Dis 2017;32(3):789–98.
51. Fikry H, Saleh LA, Abdel Gawad S. Neuroprotective effects of curcumin on the cerebellum in a rotenone-induced Parkinson's Disease Model. CNS Neurosci Ther 2022;28(5):732–48.

52. Keshk WA, Elseady WS, Sarhan NI, Zineldeen DH. Curcumin attenuates cytoplasmic/endoplasmic reticulum stress, apoptosis and cholinergic dysfunction in diabetic rat hippocampus. Metab Brain Dis 2020;35(4):637–47.
53. Lan C, Chen X, Zhang Y, Wang W, Wang WE, Liu Y, et al. Curcumin prevents strokes in stroke-prone spontaneously hypertensive rats by improving vascular endothelial function. BMC Cardiovasc Disord 2018;18(1):43.
54. Abdel-Diam MM, Samak DH, El-Sayed YS, Aleya L, Alarifi S, Alkahtani S. Curcumin and quercetin synergistically attenuate subacute diazinon-induced inflammation and oxidative neurohepatic damage, and acetylcholinesterase inhibition in albino rats. Environ Sci Pollut Res Int 2018;26(4):3659–65.
55. Song S, Nie Q, Li Z, Du G. Curcumin improves neurofunctions of 6-OHDA-induced parkinsonian rats. Pathol Res Pract 2016;212(4):247–51.
56. Akintunde JK, Farouk AA, Mogbojuri O. Metabolic treatment of syndrome linked with Parkinson's disease and hypothalamus pituitary gonadal hormones by turmeric curcumin in Bisphenol-A induced neuro-testicular dysfunction of wistar rat. Biochem Biophys Rep 2019;17:97–107.
57. Blackshaw LA, Page AJ, Partosoedarso ER. Acute effects of capsaicin on gastrointestinal vagal afferents. Neuroscience 2000;96(2):407–16.
58. Jancsó G, Such G. Effects of capsaicin applied perineurally to the vagus nerve on cardiovascular and respiratory functions in the cat. J Physiol 1983;341:359–70.
59. Mei Z-g, Wang M-z, Liu X-j, Zeng Y-b. Effect of puerarin on mRNA expression of α7nAChR, NF-κBp65 and STAT3 in brain after cerebral ischemia-reperfusion in rats. China J Trad Chin Med Pharm 2013;28(01):113–17.
60. Wang M, Mei Z, Zeng Y, Liu X. Puerarin inhibits cerebral ischemia–reperfusion injury in rats by activating the cholinergic anti-inflammatory pathway. Chin J Gerontol 2012;32(12):2563–66.
61. Kaboutari M, Asle-Rousta M, Mahmazi S. Protective effect of menthol against thioacetamide-induced hepatic encephalopathy by suppressing oxidative stress and inflammation, augmenting expression of BDNF and α7-nACh receptor, and improving spatial memory. Eur J Pharmacol 2024;981:176916.
62. Dou Y, Luo J, Yu J, Xia Y, Dai Y. Cholinergic system is involved in the therapeutic effect of madecassoside on collagen-induced arthritis in rats. International Immunopharmacology 2019;75:105813.
63. Lu Z, Xie P, Zhang D, Sun P, Yang H, Ye J, et al. 3-Dehydroandrographolide protects against lipopolysaccharide-induced inflammation through the cholinergic anti-inflammatory pathway. Biochemical Pharmacology 2018;158:305–17.

64. Ai S, Fan X, Fan L, Sun Q, Liu Y, Tao X, et al. Extraction and chemical characterization of Angelica sinensis polysaccharides and its antioxidant activity. Carbohydrate Polymers 2013;94(2):731–36.
65. Ding D, Jiao L, Wang X, Liu Z, Fan L, Li Q. Influence of breviscapine on myocardial apoptosis and NF-kB pathway signaling molecules (α7nAChR, p65 and IkB-α) in rats with myocardial ischemia-reperfusion injury. Chin J Evid Based Cardiovasc Med 2018;10(12):1480–83+87.
66. Bai J, Yuan W, Zhang L, Zeng S, Xia C, Wang H. The Regulatory Effect of Trans-Anethole on Cholinergic Anti-inflammatory Pathway in Rat Model ofChronic Obstructive Pulmonary Disease. Genomics Appl Biol 2020;39(07):3256–63.
67. Khadrawy YA, Hosny EN, Mohammed HS. Protective effect of nanocurcumin against neurotoxicity induced by doxorubicin in rat's brain. Neurotoxicology 2021;85:1–9.
68. Ma Y, Wang X, Li X, Chen X, Teng Z, Wang X, et al. COP-22 Alleviates D-Galactose-Induced Brain Aging by Attenuating Oxidative Stress, Inflammation, and Apoptosis in Mice. Mol Neurobiol 2024;61(9):6708–20.
69. Noor NA, Hosny EN, Khadrawy YA, Mourad IM, Othman AI, Aboul Ezz HS, et al. Effect of curcumin nanoparticles on streptozotocin-induced male Wistar rat model of Alzheimer's disease. Metab Brain Dis 2022;37(2):343–57.
70. Shabbir A, Rehman K, Akash MSH, Akbar M, Chaudhary Z, Panichayupakaranant P, et al. Differential neuroprotective effect of curcuminoid formulations in aluminum chloride-induced Alzheimer's disease. Environ Sci Pollut Res Int 2022;29(45):67981–96.
71. Guo Z, Luo Y, Dai Y, Zhao J. [Effect and mechanism of Curcumin and its related compounds on learning and memory disorder in mice]. Pharmacol Clin Chin Mater Med 2018;34(06):64–69.
72. Mohammed HS, Hosny EN, Khadrawy YA, Magdy M, Attia YS, Sayed OA, et al. Protective effect of curcumin nanoparticles against cardiotoxicity induced by doxorubicin in rat. Biochim Biophys Acta Mol Basis Dis 2020;1866(5):165665.
73. Zhang H, Han T, Sun LN, Huang BK, Chen YF, Zheng HC, et al. Regulative effects of essential oil from Atractylodes lancea on delayed gastric emptying in stress-induced rats. Phytomedicine 2008;15(8):602–11.
74. Satou T, Kawata A, Hasegawa A, Matsushita N, Inoue T. Effects of Inhalation of Essential Oil From Pelargonium graveolens on the Autonomic Nervous System of Awake Mice. Natural Product Communications 2022;17(6):1934578X221109419.
75. Komiya M, Sugiyama A, Tanabe K, Uchino T, Takeuchi T. Evaluation of the effect of topical application of lavender oil on autonomic nerve activity in dogs. Am J Vet Res 2009;70(6):764–9.
